# Bio-guided isolation of alpha-glucosidase inhibitory compounds from Vietnamese *Garcinia schomburgkiana* fruits: *in vitro* and *in silico* studies†

**DOI:** 10.1039/d3ra06760b

**Published:** 2023-12-04

**Authors:** Ngoc-Hong Nguyen, Y. Thien Vu, Tuan-Dat Nguyen, Truong-Tam Cao, Huy Truong Nguyen, Thi-Kim-Dung Le, Jirapast Sichaem, Dinh-Tri Mai, Tran Nguyen Minh An, Thuc-Huy Duong

**Affiliations:** a CirTech Institute, HUTECH University 475 A Dien Bien Phu Street Binh Thanh District Ho Chi Minh City 700000 Vietnam; b Faculty of Pharmacy, Ton Duc Thang University Ho Chi Minh City 700000 Vietnam nguyentruonghuy@tdtu.edu.vn; c Department of Chemistry, Ho Chi Minh City University of Education Ho Chi Minh City 700000 Vietnam huydt@hcmue.edu.vn; d Laboratory of Biophysics, Institute for Advanced Study in Technology, Ton Duc Thang University Ho Chi Minh City 700000 Vietnam; e Research Unit in Natural Products Chemistry and Bioactivities, Faculty of Science and Technology, Thammasat University Lampang Campus Lampang 52190 Thailand; f Graduate University of Science and Technology, Vietnam Academy of Science and Technology 18 Hoang Quoc Viet, Cau Giay Hanoi Vietnam; g Institute of Chemical Technology, Vietnam Academy of Science and Technology 1A TL29 Street, Thanh Loc ward, District 12 Ho Chi Minh City 700000 Vietnam; h Faculty of Chemical Engineering, Industrial University of Ho Chi Minh City 12 Nguyen Van Bao street, Ward 4, Go Vap District Ho Chi Minh City 700000 Vietnam trannguyenminhan@iuh.edu.vn

## Abstract

*Garcinia schomburgkiana* is an edible tree widely distributed in the southern region of Vietnam. Little is known about the alpha-glucosidase inhibition of the Vietnamese *Garcinia schomburgkiana*. The aim of the current study was to explore the anti-diabetic potential of *G. schomburgkiana* fruits. All the fractions of *G. schomburgkiana* were evaluated for alpha-glucosidase inhibition, followed by bioassay-guided isolation. A new compound, *epi*-guttiferone Q (1), together with ten known compounds, guttiferones I–K (2–3), hypersampsone I (4), sampsonione D (5), sampsonione H (6), β-mangostin (7), α-mangostin (8), 9-hydroxycalabaxanthone (9), and fuscaxanthone (10), were isolated and structurally elucidated. The structure of the new metabolite 1 was confirmed through 1D and 2D NMR spectroscopy, and MS analysis. To the best of our knowledge, the metabolites (except 3) have not been isolated from this plant previously. All isolated compounds were evaluated for their alpha-glucosidase inhibition. Compounds 1–6 showed potent activity with IC_50_ values ranging from 16.2 to 130.6 μM. Compound 2 was further selected for a kinetic study. The result indicated that it was a competitive type. Additionally, *in silico* docking was employed to predict the binding mechanism of 1–2 and 4–6 in the active site of alpha-glucosidase, suggesting their potential as promising anti-diabetic compounds. Molecular dynamic simulation was also applied to 1 to better understand its inhibitory mechanism.

## Abstract 2

*Garcinia schomburgkiana* is an edible tree widely distributed in the southern region of Vietnam.

## Graphical abstract

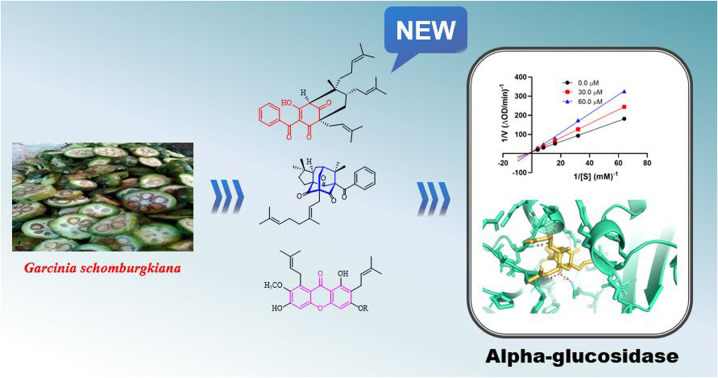

## 1. Introduction

The molecular docking model has garnered significant interest among chemists as a tool to comprehend the binding mechanisms of bioactive compounds with target proteins or enzymes.^1,2^ Validating the molecular docking data through RMSD values between ligands and the target enzyme or protein is essential.^3^ Various software programs in the Linux environment, including Gromacs and Desmond, are commonly utilized to elucidate the stable complex formed by the best docking pose and enzyme, particularly in molecular dynamics (MD) simulations.^4–6^

*Garcinia schomburgkiana*, known as Bua-dong in Vietnam, is well-known in the southern region of the country.^7^ This plant was traditionally used for the treatment of coughs, menstrual disorders, and diabetes in Vietnam.^8^ In Thailand, the ethnomedicinal uses of *G. schomburgkiana* leaves, roots, and fruit are for the improvement of menstrual blood quality, treatment of diabetes, and as a laxative.^9^ Comprehensive chemical investigations of *G. schomburgkiana* have revealed over 100 compounds in various parts of the plant, including the stem bark, roots, leaves, and fruits. To date, there have been two reports on the phytochemical constituents of *G. schomburgkiana* fruits, uncovering 14 phloroglucinols and xanthones.^8,10^ In 2016, Le and co-workers reported the presence of 12 new phloroglucinols, showing strong cytotoxicity against several cancer cell lines.^8^ In 2021, Nguyen and co-workers reported two potent alpha-glucosidase inhibitors, namely schomburgkianone I and guttiferone K, isolated from Thai fruits.^10^ Only a report regarding alpha-glucosidase inhibition of *G. schomburgkiana* fruits was published by Jaisamut and Vongsak from the Thai bio-source, indicating the strong activity with an IC_50_ value of 8.31 μg mL^−1^.^11^ In this paper, preliminary screening of alpha-glucosidase inhibition was conducted to find the most active fractions. Then, these fractions were selected for further analysis. A new compound, *epi*-guttiferone Q (1), together with ten known compounds, guttiferones I–K (2–3), hypersampsone I (4), sampsonione D (5), sampsonione H (6), β-mangostin (7), α-mangostin (8), 9-hydroxycalabaxanthone (9), and fuscaxanthone (10), were isolated and structurally elucidated. The structures of the compounds were confirmed using 1D and 2D NMR spectroscopy, as well as MS analysis. All isolated compounds were evaluated for their alpha-glucosidase inhibition. A kinetic study, molecular docking, and molecular dynamic simulation were also performed to gain a deeper understanding of the inhibitory mechanism.

## 2. Results and discussion

**Scheme 1:**
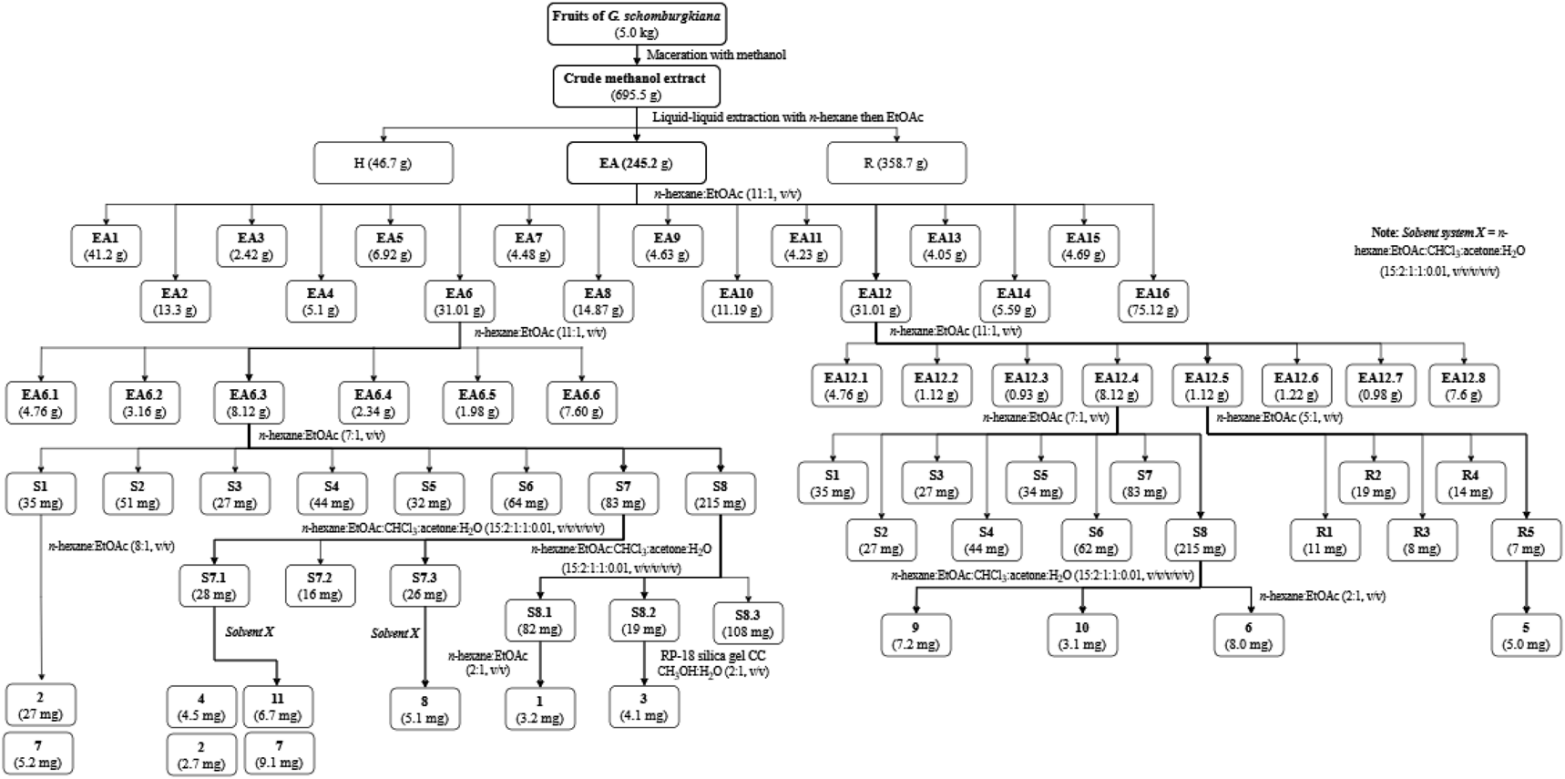
Procedures for the isolation of 1–11.

Bioactive-guided isolation was performed on the fruits of *Garcinia schomburgkiana* based on alpha-glucosidase inhibition. All extracts and fractions were screened to identify the bioactive fractions, ultimately selecting the fraction EA6 as the most active (Scheme 1) for further analysis. From this fraction, 11 compounds were isolated and structurally elucidated, including a new compound, *epi*-guttiferone Q (1), together with ten known compounds, guttiferones I–K (2–3), hypersampsone I (4), sampsonione D (5), sampsonione H (6), β-mangostin (7), α-mangostin (8), 9-hydroxycalabaxanthone (9), and fuscaxanthone (10). To the best of our knowledge, these metabolites (except 3) have been reported for the first time in this plant.

Compound 1 was obtained as a colorless gum, and its molecular formula was determined to be C_33_H_42_O_4_ by HRESIMS and from the NMR spectra (Table S1†). The ^1^H NMR data showed signals of a 1,2,4-trisubstituted benzene ring [*δ*_H_ 7.57 (1H, t, H-14, *J* = 7.5), 7.51 (2H, d, H-12, H-16, *J* = 7.5), and 7.43 (2H, d, H-13, H-15, *J* = 7.5)], three olefinic protons [*δ*_H_ 5.23, (1H brs), 5.14 (1H, brs), and 4.99 (1H, brs)], a downfiled methine at *δ*_H_ 3.41 (1H, s), and six downfield methyls [*δ*_H_ 1.74 (3H, s), 1.68 (3H, s), 1.67 (3H, s), 1.64 (3H, s), 1.62 (3H, s), and 1.56 (3H, s)] characteristic of three isoprenyl groups, one methyl at *δ*_H_ 1.00 and other methylene and methine protons in the range *δ*_H_ 1.45–1.70 ppm and *δ*_H_ 2.02–2.49 ppm. The ^13^C NMR in accordance with the HSQC spectrum indicated the presence of 33 carbon signals, including three ketone carbons (*δ*_C_ 208.2, 198.7, and 196.7), five aromatic methines (*δ*_C_ 133.1, 129.5 × 2, and 128.6 × 2), three olefinic methines (*δ*_C_ 125.2, 123.4, and 121.0), and six sp^2^ substituted carbons (*δ*_C_ 188.8, 138.5, 134.7, 133.7, 132.0, and 118.5, the first oxygenated and conjugated with a ketone group), two quaternary carbons (*δ*_C_ 65.1 and 48.8), two carbon methines (*δ*_C_ 65.6, and 41.8), five carbon methylenes (*δ*_C_ 43.1, 39.3, 31.1, 28.8, and 22.7), and seven carbon methyls (*δ*_C_ 26.2, 25.9 × 2, 18.2, 18.1, 18.0, and 17.8). The full analysis of both 1D (^1^H and ^13^C) and 2D (COSY, HSQC, and HMBC) NMR spectra suggested that 1 contained a benzoylphloroglucinol skeleton, and its NMR spectra were also close to those of guttiferones I–K (2–3), major constituents of this plant material,^10,12^ except for the absence of the isoprenyl group at C-4. Comparison of NMR data of 1 and 2 (recorded in the same acetone-*d*_6_, Table S1†) gave high similarities, indicating that they shared the same benzoylphloroglucinol skeleton. The only difference is in the presence of H-4. The methyl at *δ*_H_ 1.00 gave HMBC correlations with C-4 (*δ*_C_ 65.6), C-5 (*δ*_C_ 48.8), C-6 (*δ*_C_ 41.8), and C-18 (*δ*_C_ 39.3), supporting the position of H-4.

**Fig. 1:**
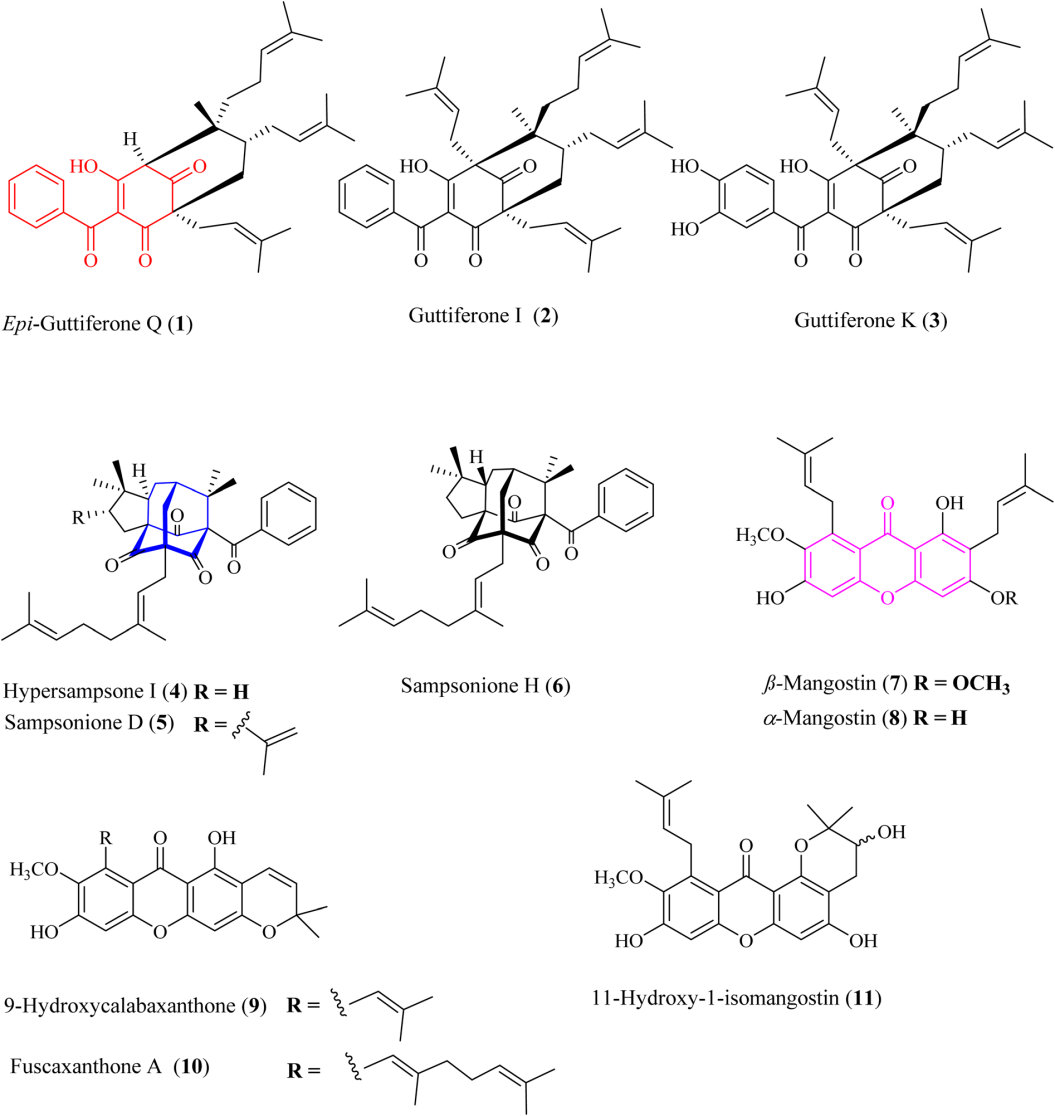
Chemical structures of 1–11. Red-highlighted fragment: benzoylphloroglucinol core, blue-highlighted fragment: homo-adamantane core, purple-highlighted fragment: xanthone core.

**Fig. 2:**
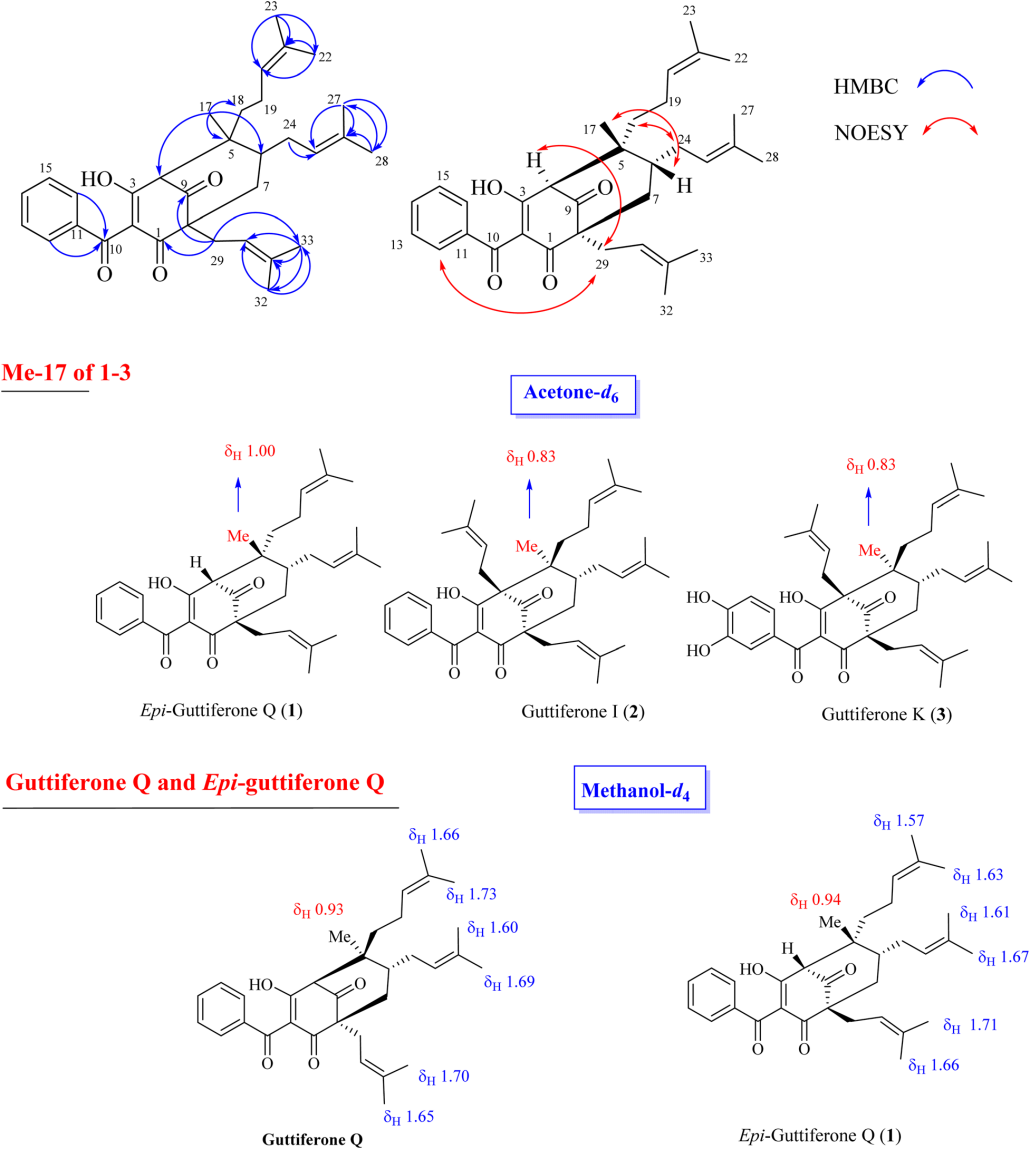
Selected HMBC and NOESY correlations of 1 and main differences of 1H NMR signals of key methyls between 1–3 and guttiferone Q.

The relative stereochemistry of 1 was determined through NOESY correlations. Initially, H_3_-17 gave a key NOE correlation with H-6, indicating their *syn*-facial while the NOE correlations of H_2_-18/H_2_-24, H_2_-29/H-4, and H_2_-29/H_2_-24 indicated their same orientation. The downfield chemical shift of H_3_-17 in 1 compared to that of 2 [*δ*_H_ 1.00 in 1*vs.* 0.83 in 2–3] indicated the reversion of the configuration of C-5. The characteristic methyl group at *δ*_H_ 0.80–0.83 that could be found in 2–3 and previously reported benzoylphloroglucinols^8^ in the same bio-source (see Fig. 2, S12 and S13†) indicated its α-orientation. The chemical structure of 1 was similar to that of guttiferone Q.^13^ To confirm the difference between 1 and guttiferone Q, ^1^H NMR spectrum of 1 was re-recorded in methanol-*d*_4_. The obvious differences in the chemical shifts of the methyl groups H_3_-22/H_3_-23/H_3_-27/H_3_-28 supported the reversion of the C-5 configuration of 1 (see Table S1† and Fig. 2). The main differences between 1 and guttiferone Q are illustrated in Fig. 2. Combined, the chemical structure of 1 was defined as shown in Fig. 1, namely *epi*-guttiferone Q.

The metabolites guttiferone I (2),^12^ hypersampsone I (4),^14^ sampsonione D (5),^14,15^ sampsonione H (6),^16^ β-mangostin (7),^17^ α-mangostin (8),^17^ 9-hydroxycalabaxanthone (9),^18^ and fuscaxanthone (10)^19^ were identified in this plant for the first time. They were classified into three types of skeletons: benzoylphloroglucinols (1–3), homo-adamantanes (4–6), and xanthones (7–11). Guttiferone I (2) was reported in *G. cowa* by Phakhatmuen and co-workers, showing a consistent result regarding alpha-glucosidase inhibition (IC_50_ 13.1 μM).^12^ The inhibition of guttiferone K (3) was isolated from Thai *G. schomburgkiana* sources in our previous reports.^10,20^ Molecular docking of 3 was reported previously.^10^ This compound was also reported their strong cytotoxicity against several cancer cell lines: HT-29, SW620, KATO-III, HepG2, CHAGO, and HCT11.^21–23^

Xanthones 7–11 are well-known in the *Garcinia* plants, *i.e.*, *G. mangostana*,^24–26^*G. fusca*,^27^*G. cylindrocarpa*,^28^*G. planchonii*.^29^ These compounds showed strong cytotoxicity against various cancer cell lines and potent alpha-glucosidase inhibition.^24–29^

Homo-adamantanes have been discovered in several *Hypericum* plants: *H. sampsonii*, *H. cohaerens*, and *H. hookerianum*.^14–16,30^ Interestingly, this is the first report of these compounds in the genus *Garcinia*. Ye and co-workers provided a comprehensive review on NMR assignments and the stereochemistry for over 100 homo-adamantanes.^16^ Based on this review, the chemical shifts of the methyl groups H_3_-22 and H_3_-23 were reassigned (Tables S4–S6†). Liu and co-workers reported the presence of 4–6 in *H. cohaerens* and assessed their cytotoxicity.^30^ Sampsonione H, isolated from *H. forrestii*, exhibited a potent inhibitory effect on protein tyrosine phosphatase 1B with an IC_50_ value of 6.63 ± 2.40 μM.^31^

### 2.1 Alpha-glucosidase inhibition and kinetic study

**Fig. 3:**
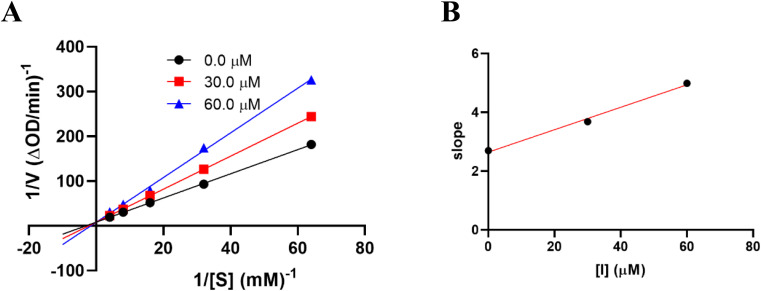
Kinetic study of 2 against alpha-glucosidase. (A) Lineweaver–Burk plot in the absence and presence of different concentrations of 2. (B) Plots of slope *versus* concentration of 2 for the determination of the inhibition constant *K*_i_.

The results of alpha-glucosidase inhibition isolated compounds obtained from *G. schomburgkiana* are presented in Table 1. Compounds 1–6 displayed strong inhibition, with IC_50_ values ranging from 16.2 to 130.6 μM, while the remaining compounds showed no activity. Compound 2 was selected for the enzyme inhibitory kinetic analysis based on the greater amount occurred in the bio-source. To better understand the kinetic parameters of alpha-glucosidase inhibition by 2, the enzyme activity was assessed by varying substrate concentrations in the absence or presence of 2. The inhibition type was identified through Lineweaver–Burk plots (Fig. 3A), where the reciprocal of the reaction rate (1/*V*) was graphed against the reciprocal of substrate concentrations (1/[S]). This approach allowed monitoring the effect of the inhibitor on both *K*_m_ and *V*_max_. As shown in Fig. 3A, the plots of 1/*V vs.* 1/[S] of 2 gave a group of straight lines that all intersected at the *y*-axis. The data revealed that with an increasing concentration of 2, *V*_max_ remained constant, while *K*_m_ increased, indicating that 2 is a competitive inhibitor of alpha-glucosidase. The results suggested that 2 bound to the active site of the enzyme, competed with the substrate and retarded the conversion of substrate into product. The inhibition constant for the inhibitor binding with free enzyme (*K*_i_) was determined from a plot of the slope (*K*_m_/*V*_m_) *versus* the inhibitor concentration (Fig. 3B). The inhibition constant (*K*_i_) of 2 was determined as 69.25 μM. In previous work, the kinetic analysis of the positive control, acarbose, showed that it acts as a competitive inhibitor with a *K*_i_ of 162.49 μM. The smaller *K*_i_ of 2 means the greater binding affinity.^32^

**Table 1:** Alpha-glucosidase inhibition (IC_50_) by compounds

Compound	IC_50_ (μM)
1	60.7 ± 3.6
2	29.4 ± 1.2
3	16.2 ± 0.7
4	130.6 ± 4.6
5	116.0 ± 2.7
6	56.9 ± 1.9
7	>200
8	>200
9	>200
10	>200
11	>200
Positive control (acarbose)	330.9 ± 4.2

Up to now, very few polyprenyl benzoylphloroglucinols have been reported their alpha-glucosidase. Guttiferone I (2) was reported in *G. cowa* by Phakhatmuen and co-workers, showing a consistent result regarding alpha-glucosidase inhibition (IC_50_ 13.1 μM).^12^ The inhibition of guttiferone K (3) and schomburgkianone I isolated from Thai *G. schomburgkiana* sources showed potent alpha-glucosidase inhibition with IC_50_ values of 34.8 and 21.2 μM, respectively.^10,20^ Guttiferone Q was thought to be a moderate inhibitor with an IC_50_ value of 117.5 μM.^29^ To date, there have been no reports on the alpha-glucosidase inhibition of homo-adamantanes 4–6. The activity of xanthones 7–11 was reported by Trinh and co-workers^29^ with IC_50_ values ranging from 31.3 to 128.9 μM. However, these compounds showed weak activity in our assay. The skeleton might play an important role in the inhibition. The inhibitory order is as follows: polyprenyl benzoylphloroglucinols (1–3) > homo-adamantanes (4–6) > xanthones (7–11).

### 2.2 *In silico* docking study

**Fig. 4:**
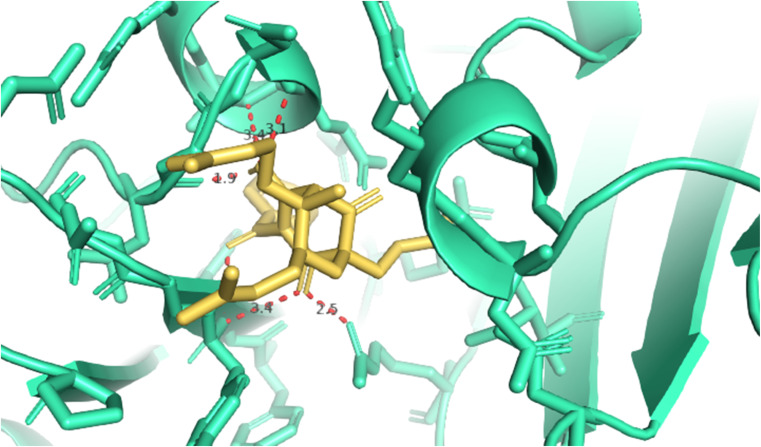
The hydrogen bondings formed 5 hydrogen bonding from 1.5, 1.9, 2.9, 3.1, and 3.4 from active atoms on the best docking pose 304 of compound 1 and residual amino acids on pocket enzyme of 4J5T enzyme *via* PYMOL software, version 2.5.4, Copyright (C) Schrodinger, LLC.

**Fig. 5:**
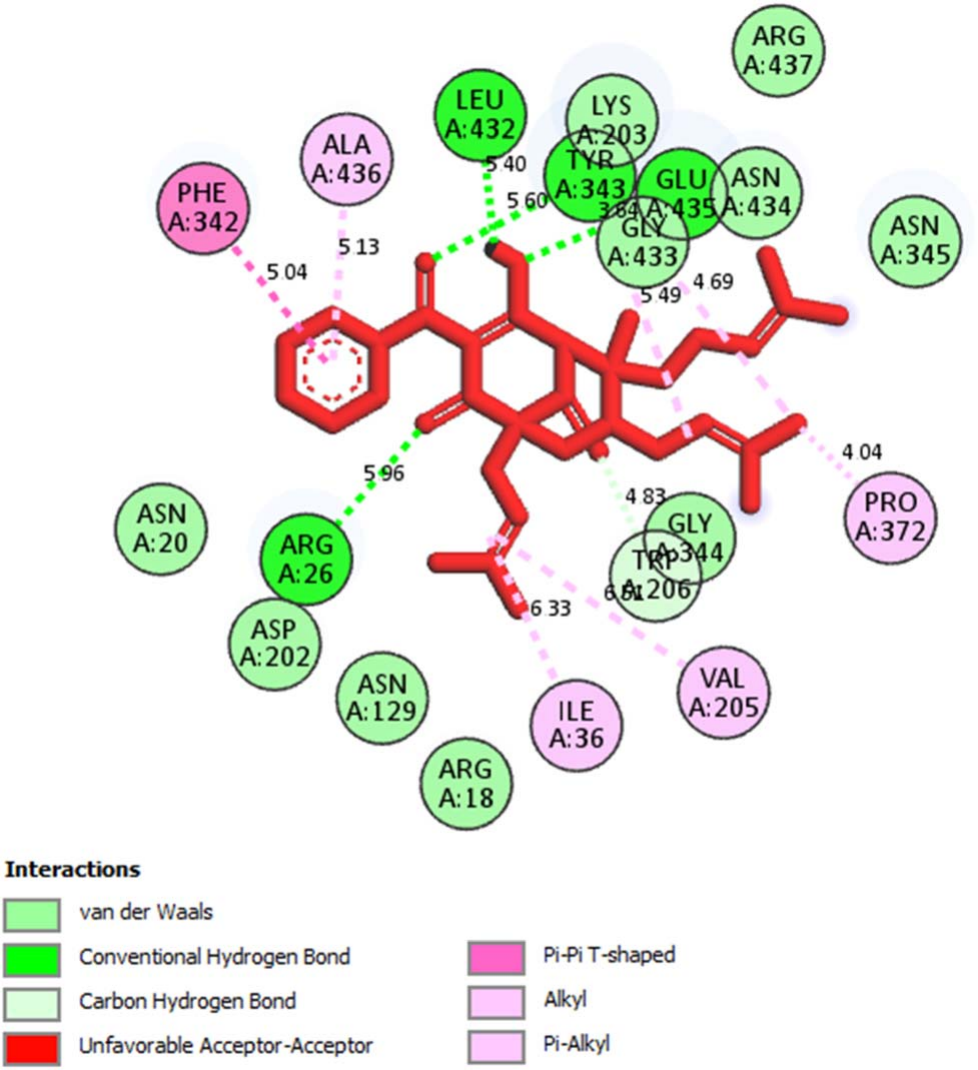
One 2D diagram showed the significant ligand interactions between pose 304, compound 1 and residual amino acid on 4J5T enzyme.

**Fig. 6:**
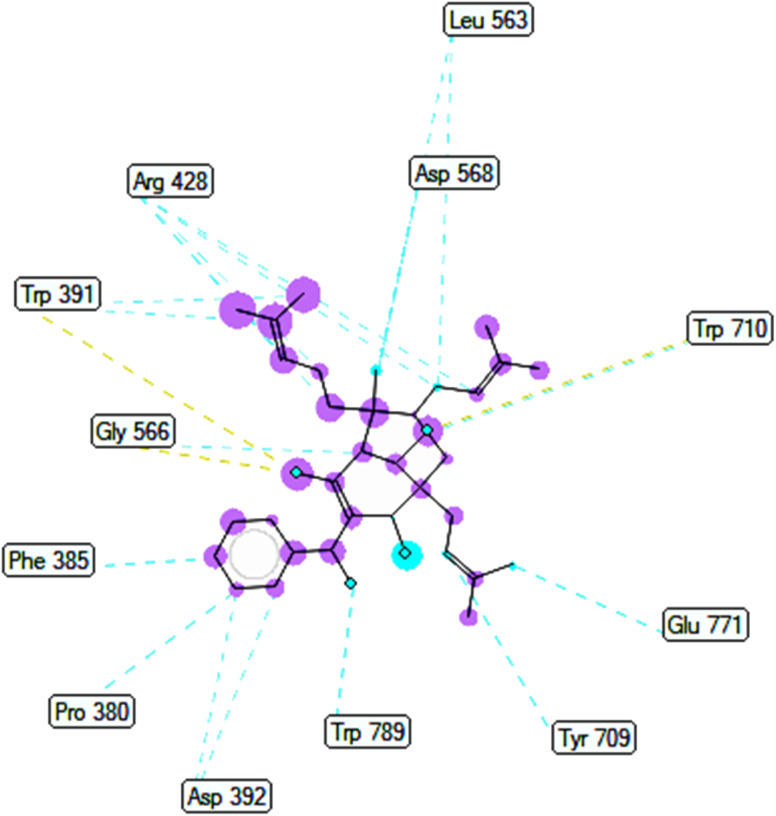
Ligand map indicated secondary interactions between pose 304, compound 1 and residual amino acid on 4J5T enzyme.

**Fig. 7:**
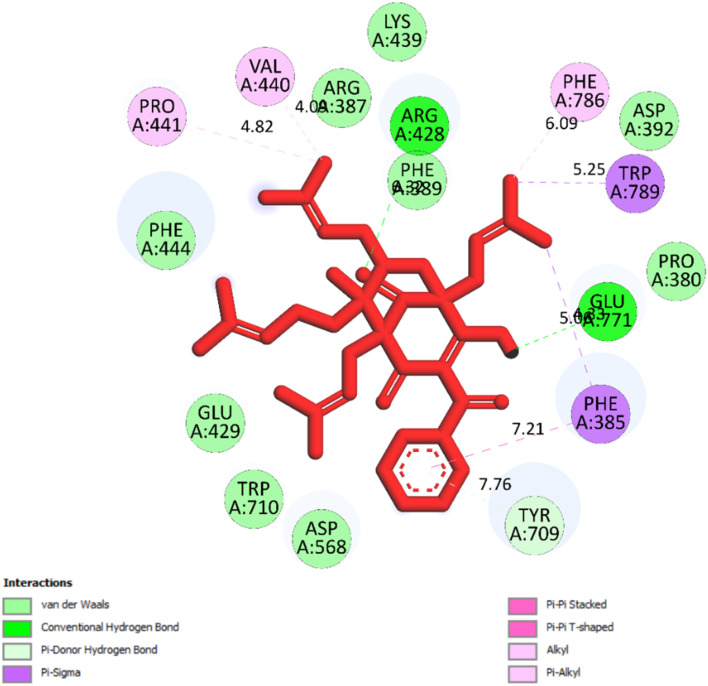
One 2D diagram showed the significant ligand interactions between pose 149/compound 2 and residual amino acid on 4J5T enzyme.

**Fig. 8:**
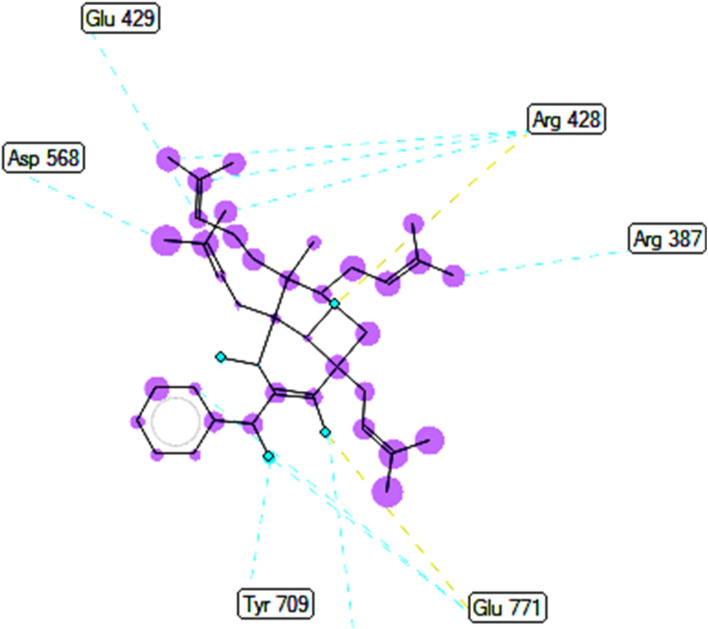
Ligand map indicated secondary interactions between pose 149, compound 2 and residual amino acid on 4J5T enzyme.

The significant docking results for 1–7, obtained using AutoDock software, are presented in Table 2 and illustrated in Fig. 4–8, as well as Fig. S14–S21.† According to Table 2, the compounds 1–7 were ranked based on their values of free Gibbs energy and inhibition constant, considering both the thermodynamic site and the ligand interaction model. The ranking, in terms of the best docking pose, is as follows: pose 304 (compound 1) > pose 332 (compound 4) > pose 82 (compound 3) > pose 149 (compound 2) > pose 490 (compound 6) > pose 489 (compound 7) > pose 160 (compound 5). This ranking is consistent with the ligand interaction model, which places these compounds in the same order as the thermodynamic site.

**Table 2:** The significant docking study results of compounds with enzyme 4J5T: PDB

Compound	Free Gibbs energy	Inhibition constant, *K*_i_	Hydrogen number	The character and bond length
1 Pose 304	−12.43	0.077	4	A: Arg 26:N-pose 304:O (2.53)
				A: Tyr 343:N-pose 304:O (2.93 Å)
				A: Glu 435:N-pose 304:O (3.05)
				Pose 304: H – A: Leu 432:O (1.89 Å)
2 Pose 149	−10.53	0.027	2	A: Arg 428:N – pose 149:O (3.08 Å)
				Pose 149:H – A: Glu 771:O (1.95 Å)
3 Pose 82	−10.22	0.032	6	A: TRP 391:N – pose 82:O (2.77 Å)
				A: Arg 428:N – pose 82:O (2.79 Å)
				A: Arg 428:N – pose 82:O (2.86 Å)
				Pose 82:H – A:Asp 392:O (1.83 Å)
				Pose 82:H – A:Asp 392:O (2.48 Å)
				Pose 82:H – A:Glu 429:O (1.82 Å)
4 Pose 332	−11.05	0.007	2	A: Arg 428:N-pose 332:O (2.65 Å)
				Pose 332:H – A:Asp 392:O (1.80 Å)
5 Pose 160	−11.77	0.0024	1	A: Arg 428:N – pose 160:O (2.45 Å)
6 Pose 490	−12.04	0.002	1	A: ARG 428:N – pose 490:O (2.67 Å)
7 Pose 489	−11.97	0.0017	1	A: Arg 428:N – pose 489:O (2.59 Å)

#### 2.2.1. Pose 304 or compound 1

The best docking pose of 1 or 304 among 500 confirmations (the number of models of 500). As indicated in Table 2 and Fig. 4–6, pose 304 among 500 models has docked to 4J5T enzyme: PDB with the values of free Gibbs energy and inhibition constant of −12.43 Kcal mol^−1^ and 0.08 μM, respectively, and it formed four hydrogen bondings with residual amino acids on 4J5T enzyme. Among them, hydrogen bonding, pose 304:H-A:Leu 432:O was the strongest bonding due to the shortest bonding length, as seen in Table 2 and Fig. 3. As in Fig. 4, one 2D diagram presented the significant ligand interactions between active atoms in this pose and residual amino acids on 4J5T. Pose 304 has been considered well in ligand interaction model due to three parts of pose 304 has been identified *via* interactions such as capping group (aromatic ring or heterocyclic ring on pose), linker (aliphatic chain or aromatic), and functional group (hydrogen bonding).^33^ The capping group of pose 304 has been discovered by pi–pi T shaped and pi–alkyl interactions from Phe 342 and Ala 436 to pi electrons of aromatic ring-hydrophobic interactions. The linker of this pose has been detected by pi-alkyl and alkyl interactions–hydrophobic interactions from Ile 36 Val 205 to alkenyl group, from Pro 372 and Gly 433 to methyl group, and from Gly 433 to carbon of alkenyl group on pose. The functional groups of this pose have been detected by hydrogen bondings from Leu 432 to oxygen atom of pose, from Tyr 343 to carbon atom of carbonyl group, Glu 435 to carbon atom of alcohol group, Leu 432 to oxygen atom of hydroxyl atom of alcohol group, and Arg 26 to oxygen atom of ketone group on this pose. Pose 304 has been considered good ligand interactions with 4J5T and proved its great inhibition on 4J5T in *in silico* docking model. As seen in Fig. 5, one ligand map has shown the secondary interactions from residual amino acid of pose 304 to active atoms on this pose such as hydrogen bondings that formed from Trp 391, Gly 566, and Tyr 710 to active atoms on this pose. Other interactions are determined by steric interactions for instance Trp 391, Arg 428, Asp 568, Trp 710, Glu 771, Tyr 709, Trp 789, Asp 392, Pro 380, Phe 385, and Gly 566 to active atoms on pose 304. There are many steric interactions have been made around this whole processing conformation of pose and consequently proved that pose 304 has been interacted strongly with 4J5T. The overlap interactions between atoms on pose and 4J5T enzyme have been depicted by violet circles on atom of pose 304. The bigger the diameters of violet circles, the stronger interactions are between pose 304 and 4J5T.

#### 2.2.2. Pose 149 or compound 2

The best docking pose 149 among 500 conformation has anchored to active center of 4J5T with the values of the free Gibbs energy and inhibition constant of −10.53 Kcal mol^−1^ and 0.027, respectively as seen in Tables 2 and it has formed two hydrogen bondings from Arg 428 and Glu 771 to oxygen atoms of pose 149. The hydrogen bonding, pose 149:H – A: Glu-771:O has shorter bond length and been identified as stronger bonding. Fig. 7 illustrated the significant interactions between this pose and active residual amino acids on 4J5T and pose 149 has interacted well to 4J5T enzyme because 3 parts of pose 149 have shown full interactions. The capping group of pose 149 has detected *via* one pi–sigma interaction forming from Phe 385 to pi electrons of phenyl group. Linker part or connecting unit (CU) has identified by hydrophobic interactions *via* pi–sima interactions from Phe 395 and Thr 789 to methyl groups on pose, and pi-alkyl or alkyl from Phe 786 to methyl group on pose. The functional groups of this pose have been discovered by hydrogen bondings from Arg 428 to oxygen atom of ketone group, and Glu 771 to oxygen atom of hydroxyl alcohol on pose. In Fig. 8, one ligand map showed the secondary interactions between this pose and active residual amino acids on 4J5T. There are many hydrogen bondings: Arg 428, and Glu 771 and steric interactions: Arg 387, Arg 428, Glu 429, Asp 568, Tyr 709, and Glu 771. The overlap interactions between atoms on pose 304 and residual amino acids are manifested by violet circles. As the size of violet circles get bigger, the interactions between this pose and 4J5T get stronger.

### 2.3 Molecular dynamic simulations

**Fig. 9:**
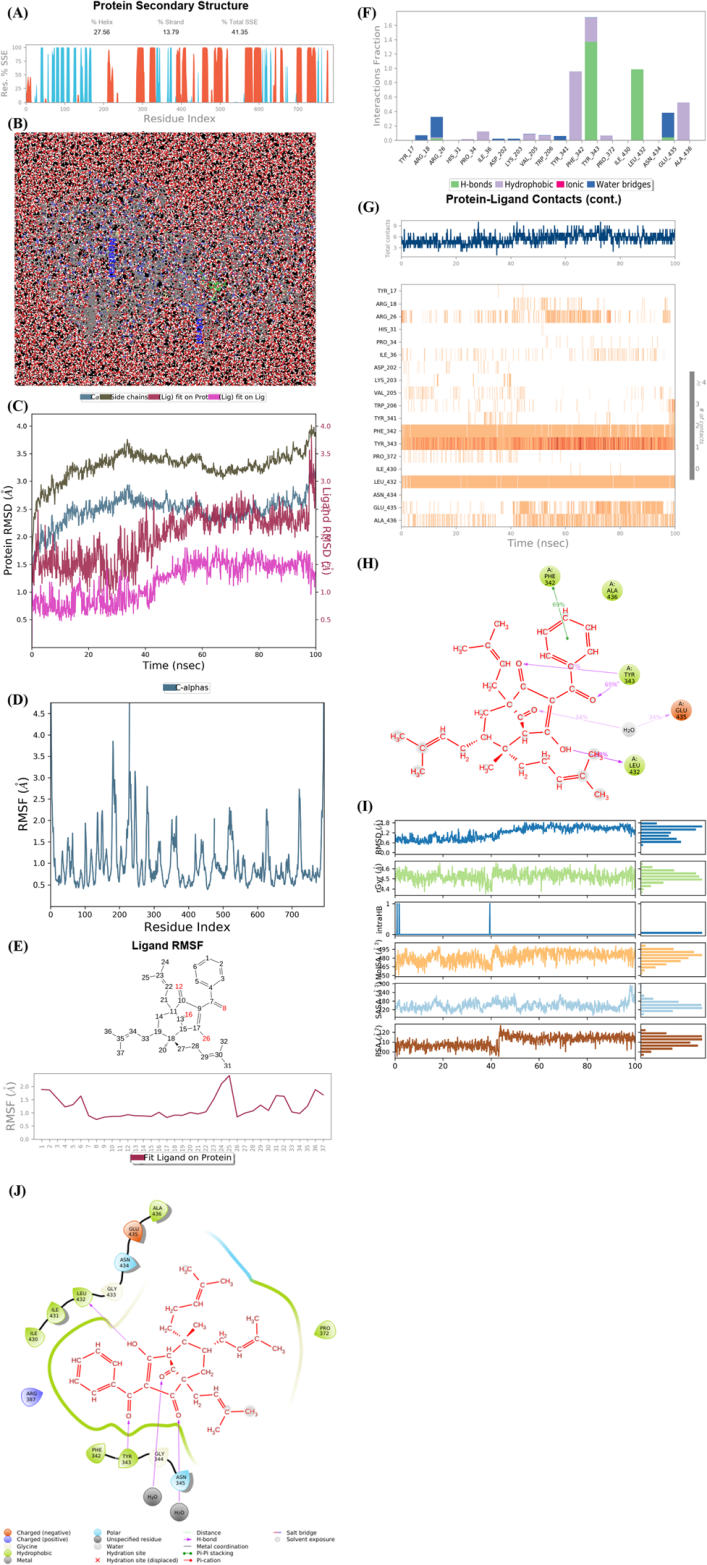
(A) Protein secondary structure are presented as 27.56% helix, 13.79% strand, and 41.35% total SSE (secondary structure elements), light blue: beta-strands and brown color: alpha-helices. (B) Simulation of complex of pose 304 and 4J5T over time period of 100 ns with simulations of 24 CPU, MDsim of job type, NPT ensemble, 300 K, 100 ns of simulation time, 83 681 atoms, 23 630 water (red color), and 0 charge. Pose 304 are green color molecule and 4J5T enzyme are indicated in grey and blue colors. (C) The RMSD curves of Cα of protein (lig blue color), side chains of protein (dark green color), ‘Lig fit on Prot’ (ligand fit on protein), dark red color, and ‘Lig fit on Lig’ (ligand fit on ligand), pink color are indicated over 0 to 100 ns of simulation course. (D) Residual peaks indicated areas of the protein that fluctuate the most during the simulation time of 100 ns such as peaks in range of 200 to 300 that is the strongest vibrations. (E) The Fit Ligand on Protein (ligand fit on enzyme 4J5T) indicated the ligand fluctuations, with respect to the protein *via* RMSF (Ligand Root Mean Square Fluctuation). In this case, the number of 25 atoms in pose 304 is strongest vibration. (F) The 4J5T-pose 304 contacts are showed and Leu 432 amino acid (H-bonds) is maintained at 100% simulation time. Tyr 343 amino acid formed the multiple contacts of same subtype with pose 304. (G) The 4J5T enzyme – pose 304 contacts appeared in whole trajectory course of 100 ns with more than one specific contact with pose 304. Such as Phe 342, Tyr 343, Leu 432, Ala 436, and Arg 26. (H) The pose 304 -4J5T contacts indicated hydrogen bonding from Glu 435 (charged negative) to oxy atom of carbonyl group *via* water bridge, from Tyr 343 (hydrophobic) to two oxygen atoms of carbonyl groups, Leu 432 (hydrophobic) to hydrogen atom of hydroxyl, and one pi–pi stacked interaction from Phe 342 (hydrophobic) to one system of pi electron of phenyl group on pose 304. (I) The properties of pose 304 are indicated such as RMSD, Radius of Gyration (rGyr), Intramolecular Hydrogen Bonds (intraHB), Molecular Surface Area (MolSA), Solvent Accessible Surface Area (SASA), and Polar Surface Area (PSA). (J) Full system interactions between pose 304 and 4J5T enzyme are exposed such as H-bonds, polar, hydrophobic, charged positive, charged negative, salt bridge.

As seen in Fig. 9A–J, the significant simulation calculation results of complex of the best docking pose 304 and 4J5T in dynamic environment with parameters such as temperature, simulation, atoms, water, and charge of 300 K, 100 ns, 83 681 atoms, 23 630 water molecules, and 0 charge are presented in profile results, simulation interactions diagram report. The information of pose 304 has shown by 37 heavy atom/79 atoms, 0 charge, 10 rotation bonds, and counter ion/salt information is ion Na of 31.547 mM and 41 positives. As seen in Fig. 9A, the secondary structure of 4J5T enzyme is 27.56% helix, 13.79% strand, and 41.35% SSE total (secondary structure elements) that were observed in whole simulation time. The protein secondary structure elements (SSE) contained the alpha-helices (red color) and beta-strands (light blue color). The plot above reported SSE distribution by residue index throughout the enzyme structure. As indicated in Fig. 9B, the complex of 4J5T and pose 304 are made around by water solvent. As seen in Fig. 9C, the values of RMSD of pose and 4J5T enzyme have been depicted, and the values of RMSDs of the enzyme such as alpha-carbon and side chain that are varied the range of 1 to 3 Å in permission ranges. ‘The lig fit lig’ means that the values of RMSD of atoms of pose 304 are changed from 0.5 to 1.5 Å (permission values <2 Å) whole simulation course from 0 to 100 ns. ‘Lig fit Prot’ exposed the RMSD of a pose 304 when the pose 304-4J5T complex is first aligned on the enzyme backbone of the reference, and then the RMSD of the ligand heavy atoms (37 atoms) is calculated. The values observed are lower than that of the RMSD of the 4J5T-alpha-carbon or side chain curves, then it is likely that the ligand has formed one equivalent from its initial binding site. As shown in Fig. 9D, the peaks indicate areas of the structure protein that fluctuate the most during the simulation. The tails (N- and C-terminal) have fluctuated more than any other part of the protein. The secondary structure elements like alpha helices and beta strands are usually more rigid than the unstructured part of the protein, and thus fluctuated less than the loop regions. As shown in Fig. 9E, the ligand root mean square fluctuation (L-RMSF) has been determined to be useful for characterizing changes in the ligand atom positions. The RMSF of pose 304 indicated the ligand's fluctuations broken down by atom, corresponding to the 2D structure above. The RMSF of pose 304 assessed insights on how ligand fragments interacted with the 4J5T and their entropic role in the binding event. The ‘Fit Ligand on Protein’ curve demonstrated the fluctuations of pose 304 with respect to the 4J5T enzyme. The pose 304-4J5T complex has been first aligned on the backbone of 4J5T and then the RMSF of this pose has been measured on the ligand heavy atoms. As seen in Fig. 9F, the 4J5T and pose 304 interactions have been monitored throughout the simulation. They have been categorized by type and summarized as hydrogen bonds, hydrophobic, ionic, and water bridges. The stacked bar charts are normalized over the course of the trajectory; for example, Leu 432 interacted hydrogen with active atom on pose 304 in 100% simulation time (100 ns). The Tyr 343 forming hydrogen with active atom on pose 304 is possible, as some residue amino acids may make multiple contacts of same hydrogen bonding with the pose 304. The Glu 435 has made hydrogen bonding with active atom on this pose *via* water bridge interaction that maintained at 40% simulation time. The Arg 26 has linked hydrogen bonding *via* water bridge that is maintained at 30% simulation time. The Phe 432 and Ala 436 have formed hydrophobic interactions with active atom on pose 304 and it keep at 70 and 50% simulation time, respectively. Other hydrophobic interactions appeared and keep less than 20% simulation time such as Ile 36, Val 205, Trp 206, and Pro 372. As shown in Fig. 9G, the interactions and contacts such as hydrogen bonding, hydrophobic, and water bridges were summarized in whole trajectory course of 100 ns with more than one specific contact with pose 304: Phe 342, Tyr 343, Leu 432, Ala 436, and Arg 26 and the blue upper line shows the total number of contacts the enzyme makes with pose 304 over the course of the orbit, while the bottom has indicated which residues interact with the pose 304 in each trajectory frame. Some residual amino acids have made more than one specific contact with pose 304 as inidcated above. As seen in Fig. 9H, the pose 304 – 4J5T contacts are presented in a detailed Fig. 9G such as Tyr 343: A chain having formed H-bonds with oxygen atom of one ketone group and another oxygen atom of ketone group that maintained at 67 and 69% simulation time. Glu 435 has linked one hydrogen bonding with one oxygen atom of ketone group *via* water bridge that are stable at 68% simulation time. Leu 432 has formed one hydrogen bonding and has kept at 98% simulation time. Finally, Phe 342 has linked one pi–pi stacking interaction to pi electron system of phenyl group and made stable at 70% simulation time. As seen in Fig. 9I, the properties of pose 304 have been indicated as the values of the RMSD, radius of gyration (rGyr), intramolecular hydrogen bonds (intra HB), molecular surface area (molSA), solvent accessible surface area (SASA) that are in ranges of 0.6 to 1.5 Å (less than 2 Å), 4.2 to 4.5 Å, 465 to 495 Å^2^, 120 to 180 Å^2^, and 100 to 120 Å^2^, respectively. As demonstrated in Fig. 9J, one full interaction system between pose 304 and 4J5T has been demonstrated by 2D diagram. The pharmacokinetics of pose 304 has exposed 2 areas including hydrophobic (green light) and polar (light blue). The hydrogen bondings have formed from the hydrogen atom of hydroxyl alcohol to Leu 432 (hydrophobic), Tyr 343 (hydrophobic amino acid) to oxygen atom of the ketone group, and water bridge interactions from water molecules to 2 atom oxygen of two ketone groups on pose 304. Pharmacophore is relative to hydrophobic interactions: Ile 430, Ile 431, Leu 432, Ala 436, Pro 372, Tyr 343, and Phe 342, polar interactions: Asn 434 and Asn 345, negative charged interaction: Glu 435, and positive charged interaction: Arg 387.

## 3. Material and methods

### 3.1 General experimental procedures

The NMR spectra were recorded on a Bruker Avance III spectrometer (500 MHz for ^1^H-NMR and 125 MHz for ^13^C-NMR). The HR-ESI-MS was recorded using an HR-ESI-MS MicrOTOF-Q mass spectrometer on an LC-Agilent 1100 LC-MSD Trap spectrometer. Thin layer chromatography (TLC) was carried out on precoated silica gel 60 F_254_ or silica gel 60 RP-18 F_254S_ (Merck). Spots were visualized by spraying with 10% H_2_SO_4_ solution followed by heating. Gravity-column chromatography was performed on silica gel 60 (0.040–0.063 mm, Himedia).

### 3.2 Plant material

Fruits of *Garcinia schomburgkiana* were collected in Tay Ninh Province (Vietnam) from July to August 2022. The scientific name of the material was identified as *Garcinia schomburgkiana* by Dr Dang Van Son, Institute of Tropical Biology, Vietnam Academy of Science and Technology. The voucher specimen (No UE-P010) was deposited in Ho Chi Minh University of Education.

### 3.3 Extraction and isolation of compounds

Dried and ground fruits of *Garcinia schomburgkiana* (5 kg) were extracted with methanol (10 L × 3) at room temperature then the filtrated solution was evaporated to give a crude extract (695.5 g). This extract was partitioned into *n*-hexane and EtOAc to afford the corresponding extracts *H* (46.7 g) and EA (245.2 g), respectively. The water-soluble solution was evaporated to obtain the extract *R* (358.7 g). The EA extract was applied to silica gel column chromatography (CC) using the mobile phase of *n*-hexane : EtOAc (11 : 1, v/v) to afford 16 fractions (EA1–EA16): EA1 (41.20 g), EA2 (13.30 g), EA3 (2.42 g), EA4 (5.1 g), EA5 (6.92 g), EA6 (31.01 g), EA7 (4.48 g), EA8 (14.87 g), EA9 (4.63 g), EA10 (11.19 g), EA11 (4.23 g), EA12 (31.01 g), EA13 (4.05 g), EA14 (5.59 g), EA15 (4.69 g), and EA16 (75.12 g). Extracts and fractions EA1–EA16 were evaluated for the alpha-glucosidase inhibition to choose the fraction for further analysis. The isolation of 1–11 was described in Scheme 1 using silica gel CC with various solvent systems.

#### 3.3.1. *Epi*-guttiferone Q (1)

Colorless oil C_38_H_50_O_5_: [α]_D_^25^ + 283 (c 0.1, CHCl_3_). IR *ν*_max_ (neat): 1706, 1522, 1368, 1229, 1045 cm^−1^. ^1^H-NMR (500 MHz, acetone-*d*_6_, *δ*, ppm, *J*/Hz): 7.57 (1H, t, *J* = 7.5 Hz, H-14), 7.51 (1H, d, *J* = 7.5 Hz, H-12), 7.51 (1H, d, *J* = 7.5 Hz, H-16), 7.43 (1H, t, *J* = 7.5 Hz, H-13), 7.43 (1H, t, *J* = 7.5 Hz, H-15), 5.23 (1H, brs, H-30), 5.14 (1H, brs, H-20), 4.99 (1H, brs, H-25), 3.41 (1H, s, H-4), 2.49 (1H, m, H-29), 2.42 (1H, m, H-19), 2.34 (1H, m, H-29), 2.32 (1H, t, *J* = 7.5 Hz, H-7), 2.20 (1H, m, H-24), 2.02 (1H, m, H-19), 2.01 (1H, m, H-7), 1.74 (3H, s, H-33), 1.70 (1H, m, H-18), 1.68 (3H, s, H-23), 1.67 (3H, s, H-22), 1.65 (1H, m, H-24), 1.63 (3H, s, H-28), 1.63 (1H, m, H-6), 1.62 (3H, s, H-32), 1.56 (3H, s, H-27), 1.47 (1H, m, H-18), 1.00 (3H, s, H-17). ^13^C-NMR (125 MHz, acetone-*d*_6_, *δ*, ppm): 208.2 (C-9), 198.7 (C-10), 196.7 (C-1), 188.8 (C-3), 138.5 (C-11), 134.7 (C-31), 133.7 (C-26), 133.1 (C-14), 132.0 (C-21), 129.5 (C-12), 129.5 (C-16), 128.6 (C-13), 128.6 (C-15), 125.2 (C-20), 123.4 (C-25), 121.0 (C-30), 118.5 (C-2), 65.6 (C-4), 65.1 (C-8), 48.8 (C-5), 43.1 (C-7), 41.8 (C-6), 39.3 (C-18), 31.1 (C-29), 28.8 (C-24), 26.2 (C-33), 25.9 (C-23), 25.9 (C-28), 22.7 (C-19), 18.2 (C-17), 18.1 (C-32), 18.0 (C-27), 17.8 (C-22). HR-ESI-MS *m*/*z*: 543.3059 [M + H_2_O + Na]^+^ (calcd for C_33_H_44_NaO_5_, 543.30864), *m*/*z*: 503.3166 [M + H]^+^ (calcd for C_33_H_43_O_4_, 503.3161).

#### 3.3.2. Hypersampsone I (4)

Colorless oil C_35_H_44_O_4_: ^1^H-NMR (500 MHz, acetone-*d*_6_, *δ*, ppm, *J*/Hz): 7.47 (1H, t, *J* = 8.0 Hz, H-19), 7.36 (1H, dd, *J* = 16.0, 8.0 Hz, H-18), 7.36 (1H, dd, *J* = 16.0, 8.0 Hz, H-20), 7.18 (1H, d, *J* = 8.0 Hz, H-17), 7.18 (1H, d, *J* = 8.0 Hz, H-21), 5.16 (1H, m, H-25), 5.08 (1H, d, *J* = 7.5 Hz, H-30), 2.63 (1H, m, H-10a), 2.63 (1H, m, H-24a), 2.53 (1H, m, H-24b), 2.52 (1H, m, H-4α), 2.40 (1H, m, H-9), 2.32 (1H, m, H-4β), 2.32 (1H, m, H-8α), 2.09 (1H, m, H-7), 2.07 (2H, m, H-27), 2.07 (2H, m, H-29), 1.99 (1H, m, H-10b), 1.82 (1H, m, H-5α), 1.80 (1H, m, H-8β), 1.68 (3H, s, H-28), 1.64 (3H, s, H-32), 1.58 (3H, s, H-33), 1.48 (3H, s, H-35), 1.44 (1H, s, H-34), 1.03 (3H, s, H-23), 0.92 (1H, s, H-22). ^13^C-NMR (125 MHz, acetone-*d*_6_, *δ*, ppm): 206.4 (C-12), 206.1 (C-2), 206.1 (C-14), 193.4 (C-15), 138.2 (C-26), 134.7 (C-16), 132.7 (C-19), 132.0 (C-31), 129.8 (C-17), 129.8 (C-21), 128.7 (C-18), 128.7 (C-20), 124.9 (C-30), 120.5 (C-25), 81.3 (C-1), 70.8 (C-3), 69.3 (C-11), 58.3 (C-7), 51.2 (C-13), 45.2 (C-10), 44.6 (C-6), 43.9 (C-9), 42.2 (C-5), 40.6 (C-27), 30.4 (C-24), 30.3 (C-23), 29.3 (C-8), 29.2 (C-4), 27.2 (C-29), 25.4 (C-34), 25.4 (C-32), 22.8 (C-35), 22.0 (C-22), 17.7 (C-33), 16.5 (C-28).

#### 3.3.3. Sampsonione D (5)

Colorless oil C_38_H_48_O_4_: ^1^H-NMR (400 MHz, CDCl_3_, *δ*, ppm, *J*/Hz): 7.39 (1H, t, *J* = 7.4 Hz, H-19), 7.26 (1H, t, *J* = 7.8 Hz, H-18), 7.26 (1H, t, *J* = 7.8 Hz, H-20), 7.12 (1H, d, *J* = 8.0 Hz, H-17), 7.12 (1H, d, *J* = 8.0 Hz, H-21), 5.11 (1H, t, *J* = 7.0 Hz, H-28), 5.05 (1H, t, *J* = 6.8 Hz, H-33), 4.91 (1H, s, H-23), 4.84 (1H, s, H-23), 3.15 (1H, m, H-5β), 2.64 (1H, m, H-4α), 2.52 (1H, m, H-10a), 2.29 (1H, m, H-8α), 2.10–2.12 (1H, m, H-9α), 2.06 (1H, m, H-4β), 2.03 (1H, m, H-7α), 2.03 (2H, m, H-32), 1.95 (2H, m, H-30), 1.89 (1H, m, H-10b), 1.80 (3H, s, H-24), 1.71 (1H, m, H-8β), 1.66 (3H, s, H-31), 1.65 (3H, s, H-35), 1.57 (3H, s, H-36), 1.46 (3H, s, H-38), 1.41 (3H, s, H-37), 0.93 (3H, s, H-26), 0.87 (3H, s, H-25). ^13^C-NMR (100 MHz, CDCl_3_, *δ*, ppm): 206.2 (C-14), 204.7 (C-12), 203.8 (C-2), 192.4 (C-15), 145.2 (C-22), 138.2 (C-29), 134.8 (C-16), 131.9 (C-19), 131.2 (C-34), 128.8 (C-17), 128.8 (C-21), 127.9 (C-18), 127.9 (C-20), 124.0 (C-33), 118.9 (C-28), 111.7 (C-23), 80.8 (C-1), 73.9 (C-3), 68.8 (C-11), 57.1 (C-7), 54.9 (C-5), 50.6 (C-13), 44.2 (C-6), 43.8 (C-9), 42.5 (C-10), 39.8 (C-30), 34.3 (C-4), 29.3 (C-27), 28.8 (C-8), 27.0 (C-26), 26.7 (C-25), 26.5 (C-32), 25.6 (C-35), 25.1 (C-38), 23.7 (C-24), 22.7 (C-37), 17.5 (C-36), 16.3 (C-31).

#### 3.3.4. Sampsonione H (6)

Colorless oil C_35_H_44_O_4_: ^1^H-NMR (400 MHz, CDCl_3_, *δ*, ppm, J/Hz): 7.38 (1H, t, *J* = 7.6 Hz, H-19), 7.26 (1H, dd, *J* = 12.4, 8.8 Hz, H-18), 7.26 (1H, dd, *J* = 12.4, 8.8 Hz, H-20), 7.06 (1H, d, *J* = 7.2 Hz, H-17), 7.06 (1H, d, *J* = 7.2 Hz, H-21), 5.30 (1H, t, *J* = 8.0 Hz, H-25), 5.06 (1H, brs, H-30), 2.61 (1H, m, H-24a), 2.61 (1H, m, H-24b), 2.50 (1H, dd, *J* = 10.8, 7.2 Hz, H-10a), 2.43 (1H, m, H-4β), 2.37 (1H, d, *J* = 11.4, 7.6 Hz, H-4α), 2.21 (1H, d, *J* = 16.4 Hz, H-10b), 2.10 (1H, m, H-9), 2.07 (2H, m, H-27), 2.07 (2H, m, H-29), 1.95 (1H, m, H-7), 1.92 (1H, m, H-8α), 1.75 (1H, m, H-5α), 1.75 (1H, m, H-5β), 1.75 (1H, m, H-8β), 1.66 (3H, s, H-28), 1.66 (3H, s, H-32), 1.59 (3H, s, H-33), 1.42 (3H, s, H-34), 1.39 (3H, s, H-35), 1.01 (3H, s, H-22), 0.94 (3H, s, H-23). ^13^C-NMR (100 MHz, CDCl_3_, *δ*, ppm): 207.5 (C-12), 203.8 (C-2), 203.8 (C-14), 193.4 (C-15), 139.1 (C-26), 135.2 (C-16), 132.3 (C-19), 131.8 (C-31), 128.7 (C-17), 128.7 (C-21), 128.4 (C-18), 128.4 (C-20), 124.4 (C-30), 119.2 (C-25), 79.7 (C-1), 75.1 (C-3), 67.7 (C-11), 55.6 (C-7), 47.8 (C-13), 44.5 (C-6), 42.7 (C-5), 42.5 (C-9), 40.2 (C-27), 35.3 (C-10), 29.1 (C-24), 28.6 (C-22), 28.2 (C-4), 26.7 (C-29), 25.9 (C-32), 25.2 (C-34), 23.8 (C-8), 22.6 (C-35), 20.7 (C-23), 17.8 (C-33), 16.5 (C-28).

### 3.4 Alpha-glucosidase inhibition assay

Evaluation of the inhibitory activity of 1–11 against yeast alpha-glucosidase followed a previous procedure.^34^

### 3.5 Kinetic study of α-glucosidase inhibition of 2

**Fig. 10:**
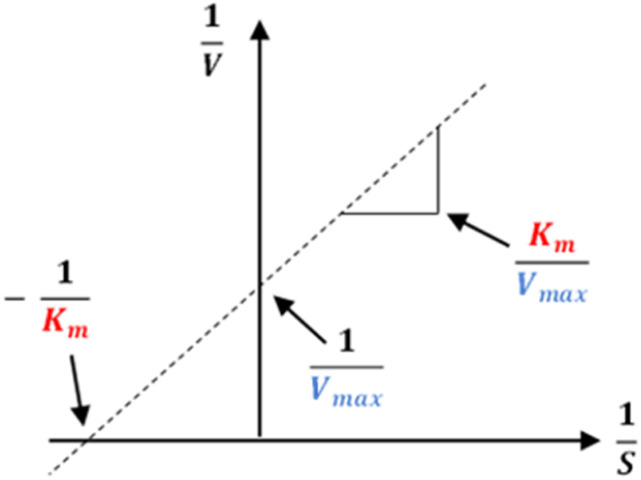
Lineweaver–Burk plot.

The kinetics of 2 against alpha-glucosidase was conducted by varying the concentration of *p*-NPG substrate in the absence or presence of tested compound at different concentrations (0, 30.0, and 60.0 μM). The Lineweaver–Burk plots were used to determine the inhibition type. In addition, the inhibition constant *K*_i_ is an indication of the potency of an inhibitor and was determined from secondary plots of 1/*V vs.* [I].^35^ The Lineweaver–Burk double-reciprocal plot (1/*V vs.* 1/[S]) was employed to discern the *V*_max_, *K*_max_, and mode of inhibition (Fig. 10).
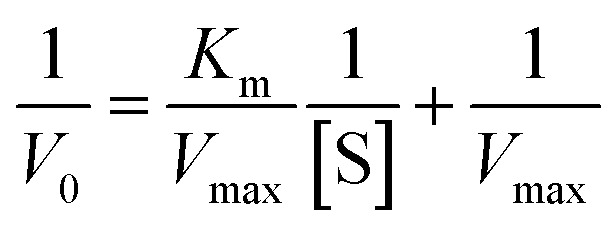


### 3.6 *In silico* molecular docking model

**Scheme 2:**
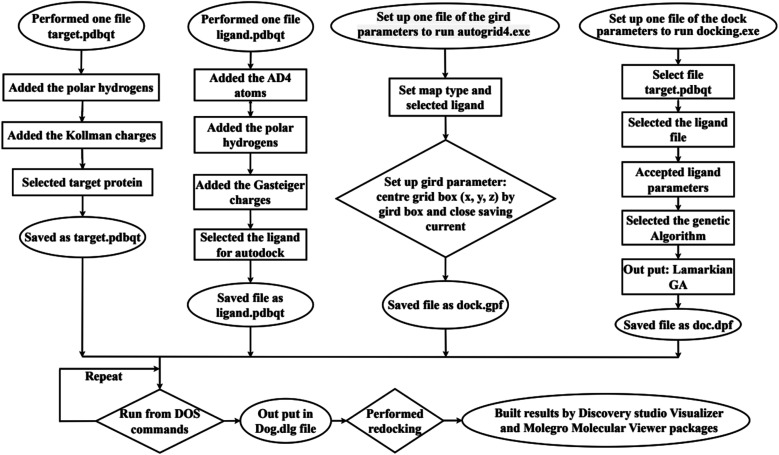
General procedure for docking of pose 304 to 4J5T enzyme: PDB.

**Scheme 3:**
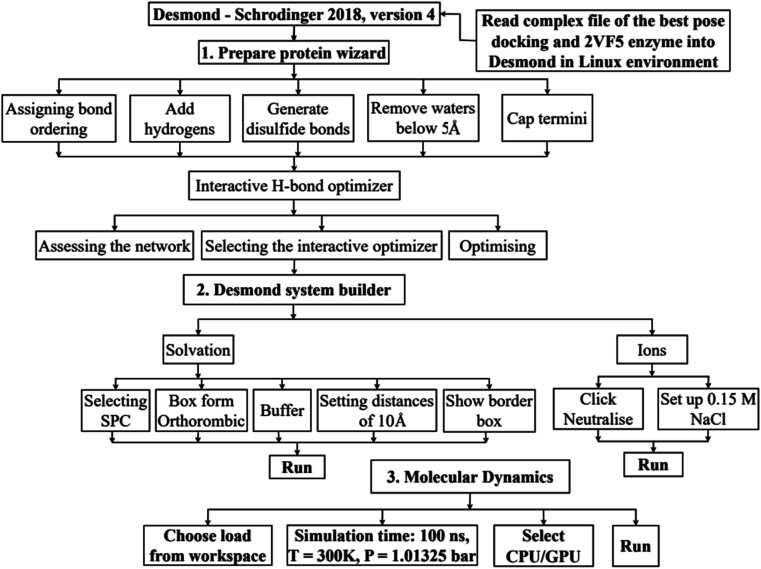
Procedure for molecular dynamic simulation of complex of the best pose 304 and 4J5T enzyme by Desmond software in Linux environment or Ubuntu.

*In silico*, molecular docking model of compounds to 4J5T enzyme: PDB have implemented based on Scheme 2 and 3. The active center of enzyme was determined based on article The docking parameters are set up in dock.glg file including 0.5 of space, the numbers of elements on (*X*, *Y*, *Z*) of (80, 90, 80), coordinators of active center of (−18.418–20.917 8.099). MD simulation has been performed on Scheme 2.^4–6,36,37^

## 4. Conclusions

From *G. schomburgkiana* fruits, a bioassay-guided isolation was applied, resulting in the presence of 11 compounds. They were structurally elucidated *epi*-guttiferone Q (1), guttiferones I–K (2–3), hypersampsone I (4), sampsonione D (5), sampsonione H (6), β-mangostin (7), α-mangostin (8), 9-hydroxycalabaxanthone (9), and fuscaxanthone (10). To the best of our knowledge, compounds 1–2 and 4–11 were reported for the first time from this plant. Notably, compounds 1–6 displayed potent activity. Compound 2 was further selected for a kinetic study, indicating that it was a competitive type. *In silico* docking and molecular dynamic simulation were employed to predict well the most stable binding mechanism of a new compound 1 or pose 304 to 4J5T enzyme.

## Conflicts of interest

No potential conflict of interest was reported by the authors.

## Supplementary Material

RA-013-D3RA06760B-s001
