# Supplementary material for: Bio-guided isolation of alpha-glucosidase inhibitory compounds from Vietnamese Garcinia schomburgkiana fruits: in vitro and in silico studies
Source: RSC Adv. 2023 Dec 4;13(50):35408–21. doi: 10.1039/d3ra06760b (PMC10694853; doi:10.1039/d3ra06760b)
Supplement: RA-013-D3RA06760B-s001 [file RA-013-D3RA06760B-s001.pdf]

Electronic Supplementary Material (ESI) for RSC Advances.
This journal is © The Royal Society of Chemistry 2023

Table S1. ^{1}H NMR (500 MHz, δ_H, multi, (*J* in Hz) and ^{13}C NMR (125 MHz) spectral data of **1** and guttiferone Q

No.	***Epi*-guttiferone Q (1)** (Acetone-d_6)			**Guttiferone Q** (Methanol-d_4)	
	$\delta_H{}^a$, *J* (Hz)	$\delta_C{}^a$	$\delta_H{}^b$, *J* (Hz)	$\delta_H{}^b$, *J* (Hz)	$\delta_C{}^b$
1		196.7			196.6
2		118.5			118.4
3		188.8			188.4
4	3.41 (*s*)	65.6		3.35 (*s*)	67.7
5		48.8			48.3
6	1.63 (*m*)	41.8		1.78 (*m*)	42.1
7	2.32 (*t*, *J* = 7.5)<br>2.01 (*m*)	43.1		2.06 (*dd*, *J* = 13.2, 4.0)<br>1.60 (*t*, *J* = 13.2)	42.9
8		65.1			64.9
9		208.2			208.0
10		198.7			198.3
11		138.5			139.5
12	7.51 (*d*, *J* = 7.5)	129.5	7.57 (*d*, *J* = 7.5)	7.56 (*m*)	129.7
13	7.43 (*t*, *J* = 7.5)	128.6	7.38 (*t*, *J* = 7.5)	7.40 (*t*, *J* = 7.7)	128.9
14	7.57 (*t*, *J* = 7.5)	133.1	7.53 (*t*, *J* = 7.5)	7.54 *m*	133.3
15	7.43 (*t*, *J* = 7.5)	128.6	7.38 (*t*, *J* = 7.5)	7.40 (*t*, *J* = 7.7)	128.9
16	7.51 (*d*, *J* = 7.5)	129.5	7.57 (*d*, *J* = 7.5)	7.56 (*m*)	129.7
17	1.00 (*s*)	18.2	0.94 (*s*)	0.93 (*s*)	18.4
18	1.70 (*m*)<br>1.47 (*m*)	39.3		1.67 (*m*)<br>1.46 (*td*, *J* = 12.8, 4.1)	39.8
19	2.42 (*m*)<br>2.02 (*m*)	22.7		2.36 (*m*)<br>2.06 (*m*)	23.0
20	5.14 (*m*)	125.2	5.09 (*m*)	5.12 (*brt*, *J* = 7.0)	125.4
21		132.0		–	132.5
22	1.67 (*s*)	17.8	1.57 (*s*)	1.66 (*s*)	18.2
23	1.68 (*s*)	25.9	1.63 (*s*)	1.73 (*s*)	26.0
24	2.20 (*m*)<br>1.65 (*m*)	28.8		2.20 (*m*)<br>1.70 (*m*)	29.1
25	4.99 (*m*)	123.4	5.01 (*m*)	5.05 (*brt*, *J* = 6.5)	123.6
26		133.7		–	134.4
27	1.56 (*s*)	18.0	1.61 (*s*)	1.60 (*s*)	17.8
28	1.63 (*s*)	25.9	1.67 (*s*)	1.69 (*s*)	25.9
29	2.49 (*m*)<br>2.34 (*m*)	31.1		2.42 (*m*)	31.1
30	5.23 (*m*)	121.0	5.16 (*m*)	5.18 (*br t*, *J* = 7.3)	121.1

31		134.7		–	135.2
32	1.62 (*s*)	18.1	1.66 (*s*)	1.65 (*s*)	18.0
33	1.74 (*s*)	26.2	1.71 (*s*)	1.70 (*s*)	26.2

[a]acetone-d_6
[b]methanol-d_4

Table S2. ^{1}H NMR (500 MHz, δ_H, multi, (*J* in Hz) and ^{13}C NMR (125 MHz) data of compounds **1** and **2** in acetone-d_6.

No.	**1** (acetone-d_6)		**2** (acetone-d_6)	
	δ_H **(ppm),** ***J*** **(Hz)**	δ_C **(ppm)**	δ_H **(ppm),** ***J*** **(Hz)**	δ_C **(ppm)**
1	–	196.7	–	206.3
2	–	118.5	–	69.9
3	–	188.8	–	43.4
4	3.41 (*s*)	65.6	–	43.3
5	–	48.8	–	43.1
6	1.63 (*m*)	41.8	–	62.8
7	2.32 (*t*, *J* = 7.5)<br>2.01 (*m*)	43.1	–	195.2
8	–	65.1	–	121.0
9	–	208.2	–	191.8
10	–	198.7	2.76 (*m*, 1H)<br>2.57 (*m*, 1H)	26.2
11	–	138.5	4.92 (*m*, 1H)	121.0
12	7.51 (*d*, *J* = 7.5)	129.5	–	134.9
13	7.43 (*t*, *J* = 7.5)	128.6	1.64 (*s*, 3H)	18.2
14	7.57 (*t*, *J* = 7.5)	133.1	1.64 (*s*, 3H)	26.2
15	7.43 (*t*, *J* = 7.5)	128.6	0.86 (*s*, 3H)	14.3
16	7.51 (*d*, *J* = 7.5)	129.5	–	37.1
17	1.00 (*s*)	18.2	–	24.5
18	1.70 (*m*)<br>1.47 (*m*)	39.3	4.99 (*s*, 1H)	125.2
19	2.42 (*m*)<br>2.02 (*m*)	22.7	–	133.0
20	5.14 (*br s*)	125.2	1.70 (*s*, 3H)	17.8
21	–	132.0	1.56 (*s*, 3H)	25.6
22	1.67 (*s*)	17.8	–	30.4
23	1.68 (*s*)	25.9	5.08 (*m*, 1H)	123.5
24	2.20 (*m*)<br>1.65 (*m*)	28.8	–	132.1

25	4.99 (*br s*)	123.4	1.69 (*s*, 3H)	25.8
26	–	133.7	1.59 (*s*, 3H)	18.1
27	1.56 (*s*)	18.0	–	198.6
28	1.63 (*s*)	25.9	–	138.3
29	2.49 (*m*)<br>2.34 (*m*)	31.1	7.51 (*d*, *J* = 7.5, 1H)	130.5
30	5.23 (*br s*)	121.0	7.42 (*t*, *J* = 7.5, 1H)	129.6
31	–	134.7	7.57 (*t*, *J* = 7.5, 1H)	133.3
32	1.62 (*s*)	18.1	7.42 (*t*, *J* = 7.5, 1H)	129.6
33	1.74 (*s*)	26.2	7.51 (*d*, *J* = 7.5, 1H)	130.5

Table S3. ^{1}H NMR (500 MHz, δ_H, multi, (*J* in Hz) and ^{13}C NMR (125 MHz) spectral data comparison of compound **3** in acetone-d_6.

**No.**	**3** (acetone-d_6)		**Guttiferone K** (methanol-d_4)	
	δ_H **(ppm),** ***J*** **(Hz)**	δ_C **(ppm)**	δ_H **(ppm),** ***J*** **(Hz)**	δ_C **(ppm)**
1	–	196.9	–	196.7
2	–	117.8	–	119.9
3	–	191.9	–	191.0
4	–	68.0	–	69.4
5	–	51.3	–	51.6
6	1.60 (*m*)	40.3	1.75 (*m*)	42.0
7	2.07 (*m*)<br>1.44 (*m*)	42.8	2.03 (*dd*, *J* = 13.3, 3.3)<br>1.44 (*dd*, *J* = 13.3, 3.3)	43.2
8	–	65.1	–	64.1
9	–	207.9	–	209.2
10	–	196.5	–	196.7
11	–	129.8	–	130.3
12	7.19 (*d*, *J* = 2.0)	117.4	7.20 (*d*, *J* = 2.1)	117.4
13	–	151.3	–	146.5
14	–	145.4	–	152.6
15	6.80 (*d*, *J* = 8.0)	114.9	6.69 (*d*, *J* = 8.4)	115.3
16	7.01 (*dd*, *J* = 8.5, 2.0)	124.6	6.95 (*dd*, *J* = 8.4, 2.1)	125.1
17	2.79 (*dd*, *J* = 14.5, 8.5)<br>2.68 (*m*)	26.5	2.73 (*dd*, *J* = 13.0, 7.8)<br>2.65 (*dd*, *J* = 13.0, 4.3)	26.7
18	4.92 (*m*)	121.4	4.88 (*m*)	121.5
19	–	134.2	–	135.2
20	1.70 (*s*)	18.3	1.69 (*s*)	18.5
21	1.66 (*s*)	25.9	1.62 (*s*)	26.4
22	0.86 (*s*)	17.8	0.81 (*s*)	16.4

23	1.81 (*m*)<br>1.77 (*m*)	36.9	1.68 (*m*)	37.6
24	2.12 (*m*)<br>1.99 (*m*)	25.0	2.07 (*m*)<br>1.77 (*m*)	30.2
25	5.01 (*br t*, *J* = 6.8)	125.4	5.00 (*br t, J* = 7.0)	123.7
26	–	131.9	–	134.7
27	1.66 (*s*)	18.2	1.67 (*s*)	18.3
28	1.60 (*s*)	25.9	1.57 (*s*)	26.0
29	2.55 (*m*)<br>2.45 (*m*)	31.4	2.51 (*dd*, *J* = 14.5, 8.8)<br>2.44, (*dd*, *J* = 14.5, 8.8)	31.8
30	5.09 (*br t*, *J* = 6.0)	120.9	5.10 (*br t, J* = 7.1)	121.1
31	–	134.7	–	135.7
32	1.67 (*s*)	18.1	1.67 (*s*)	18.4
33	1.70 (*s*)	26.2	1.71 (*s*)	26.4
34	1.99 (*m*)	26.4	1.97 (*m*)	25.3
35	5.18 (*br t*, *J* = 7.0)	123.5	5.04 (*br t*, *J* = 6.9)	125.6
36	–	133.8	–	132.7
37	1.66 (*s*)	25.9	1.66 (*s*)	26.1
38	1.57 (*s*)	18.1	1.59 (*s*)	18.0

Table S4. ^{1}H NMR (500 MHz, δ_H, multi, (*J* in Hz) and ^{13}C NMR (125 MHz) data of compound **4** in acetone-d_6.

**No.**	**4**<br>(acetone-d_6)		**Hypersampsone I**<br>(pyridine-d_5)	
	δ_H **(ppm),** ***J*** **(Hz)**	δ_C **(ppm)**	δ_H **(ppm),** ***J*** **(Hz)**	δ_C **(ppm)**
1	–	81.3	–	81.9
2	–	206.1	–	205.8
3	–	70.8	–	76.6
4α	2.52 (*m*)	29.2	2.72 (*m*)	29.0
4β	2.32 (*m*)		2.56 (*ddd*, *J* = 14.1, 9.4, 3.9)	
5α	1.82 (*m*)	42.2	1.78<br>(over load)	41.8
5β			2.00 (*m*)	
6	–	44.6	–	44.3
7	2.09 (*m*)	58.3	2.20 (*m*)	58.0
8α	2.32 (*m*)	29.3	2.25 (*m*)	28.9
8β	1.80 (*m*)		1.55<br>(over load)	
9	2.40 (*m*)	43.9	2.14 (m)	43.5
10a	2.63 (*m*)	45.2	2.65 (*dd*, *J* = 13.9, 6.1)	45.1

10b	1.99 (*m*)		1.98 (*m*)	
11	–	69.3	–	69.1
12	–	206.4	–	206.1
13	–	51.2	–	51.9
14	–	206.1	–	205.9
15	–	193.4	–	193.7
16	–	134.7	–	135.6
17	7.18 (*d*, J = 8.0)	129.8	7.54 (*br d*, *J* = 7.8)	129.8
18	7.36 (*dd*, *J* = 16.0, 8.0)	128.7	7.37 (*m*)	128.5
19	7.47 (*t*, *J* = 7.0)	132.7	7.40 (*m*)	132.5
20	7.36 (*dd*, *J* = 16.0, 8.0)	128.7	7.37 (*m*)	128.5
21	7.18 (*d*, *J* = 8.0)	129.8	7.54 (*br d*, *J* = 7.8)	129.8
22	0.92 (*s*)	22.0	0.95 (*s*)	21.8
23	1.03 (*s*)	30.3	0.92 (*s*)	29.9
24a	2.63 (*m*)	30.4	2.86 (*dd*, *J* = 14.5, 7.8)	30.4
24b	2.53 (*m*)		2.74 (*m*)	
25	5.16 (*m*)	120.5	5.42 (*t*, J = 6.5)	120.2
26	–	138.2	–	138.1
27	2.07 (*m*)	40.6	2.07 (*m*)	40.3
28	1.68 (*s*)	16.5	1.77 (*s*)	16.6
29	2.07 (*m*)	27.2	2.12 (*m*)	26.9
30	5.08 (*d*, *J* = 7.5)	124.9	5.17 (*br t*, *J* = 7.4)	124.6
31	–	132.0	–	131.4
32	1.64 (*s*)	25.4	1.66 (*s*)	25.8
33	1.58 (*s*)	17.7	1.56 (*s*)	17.7
34	1.44 (*s*)	25.4	1.58 (*s*)	25.7
35	1.48 (*s*)	22.8	1.62 (*s*)	22.8

Table S5. ^{1}H NMR (400 MHz, δ_H, multi, (*J* in Hz) and ^{13}C NMR (100 MHz) spectral data comparison of compound **5** in $CDCl_3$.

**No.**	**5** ($CDCl_3$)		**Sampsonione D** ($CDCl_3$)	
	δ_H **(ppm),** J **(Hz)**	δ_C **(ppm)**	δ_H **(ppm),** J **(Hz)**	δ_C **(ppm)**
1	–	80.8	–	80.8
2	–	203.8	–	203.9
3	–	73.9	–	73.9
4α	2.64 (1H, *m*)	34.3	2.63 (1H, *t*, *J* = 12.6)	34.4
4β	2.06 (1H, *m*)		2.08 (1H, *dd*, *J* = 12.4, 8.8)	
5β	3.15 (1H, *m*)	54.9	3.12 (1H, *dd*, *J* = 12.0, 8.9)	54.9
6	–	44.2	–	44.3
7α	2.03 (1H, *m*)	57.1	2.03 (1H, *m*)	57.2

8α	2.29 (1H, *m*)	28.8	2.28 (1H, *m*)	28.9
8β	1.71 (1H, *m*)		1.71 (1H, *m*)	
9α	2.10-2.12 (1H, *m*)	43.8	2.13 (1H, *m*)	43.9
10a	2.52 (1H, *m*)	42.5	2.52 (1H, *dd*, *J* = 14.0, 8.6)	42.6
10b	1.89 (1H, *m*)		1.88 (1H, *d*, *J* = 14.1)	
11	–	68.8	–	68.9
12	–	204.7	–	204.7
13	–	50.6	–	50.7
14	–	206.2	–	206.2
15	–	192.4	–	192.4
16	–	134.8	–	134.8
17	7.12 (1H, *d*, *J* = 8.0)	128.8	7.11 (1H, *d*, *J* = 7.7)	128.8
18	7.26 (1H, *t*, *J* = 7.8)	127.9	7.26 (1H, *t*, *J* = 7.9)	127.9
19	7.39 (1H, *t*, *J* = 7.4)	131.9	7.39 (1H, *t*, *J* = 7.7)	131.3
20	7.26 (1H, *t*, *J* = 7.8)	127.9	7.26 (1H, *t*, *J* = 7.9)	127.9
21	7.12 (1H, *d*, *J* = 8.0)	128.8	7.11 (1H, *d*, *J* = 7.7)	128.8
22	–	145.2	–	145.2
23	4.91 (1H, *s*)	111.7	4.91 (1H, *s*)	111.8
	4.84 (1H, *s*)		4.84 (1H, *s*)	
24	1.80 (3H, *s*)	23.7	1.80 (3H, *s*)	23.8
25	0.87 (3H, *s*)	26.7	0.86 (3H, *s*)	26.7
26	0.93 (3H, *s*)	27.0	0.93 (3H, *s*)	27.0
27	2.16 (2H, *m*)	29.3	nd	29.3
28	5.11 (1H, *t*, *J* = 7.0)	118.9	5.11 (1H, *t*, *J* = 5.7)	118.9
29	–	138.2	–	138.3
30	1.95 (2H, *m*)	39.8	1.98 (2H, *m*)	39.5
31	1.66 (3H, *s*)	16.3	1.66 (3H, *s*)	16.3
32	2.03 (2H, *m*)	26.5	2.03 (2H, *m*)	26.5
33	5.05 (1H, *t*, *J* = 6.8)	124.0	5.05 (1H, *t*, *J* = 4.3)	124.0
34	–	131.2	–	131.3
35	1.65 (3H, *s*)	25.6	1.65 (3H, *s*)	25.6
36	1.57 (3H, *s*)	17.5	1.57 (3H, *s*)	17.6
37	1.41 (3H, *s*)	22.7	1.40 (3H, *s*)	22.7
38	1.46 (3H, *s*)	25.1	1.46 (3H, *s*)	25.2

Table S6. ^{1}H NMR (400 MHz, δ_H, multi, (*J* in Hz) and ^{13}C NMR (100 MHz) data of compound **6** in $CDCl_3$.

No.	6 (CDCl$_3$)		Sampsonione H (CDCl$_3$)	
	**δ_H (ppm), *J* (Hz)**	**δ_C (ppm)**	**δ_H (ppm), *J* (Hz)**	**δ_C (ppm)**
1	–	79.7	–	81.1
2	–	203.8	–	203.3
3	–	75.1	–	74.8
4α	2.37 (*dd*, *J* = 11.4, 7.6)	28.2	2.37 (*dd*, *J* = 13.7, 6.7)	27.9
4β	2.43 (*m*)		2.43 (*dd*, *J* = 13.7, 7.0)	
5α	1.75 (*m*)	42.7	1.72 (*m*)	42.4
5β	1.75 (*m*)		1.72 (*m*)	
6	–	44.5	–	44.2
7	1.95 (*m*)	55.6	1.92 (*m*)	55.3
8α	1.92 (*m*)	23.8	1.92 (*m*)	23.5
8β	1.75 (*m*)		1.72 (*m*)	
9	2.10 (*m*)	42.5	2.07 (*m*)	42.2
10a	2.50 (*dd*, *J* = 10.8, 7.2)	35.3	2.50 (*dd*, *J* = 14.4, 5.3)	35.0
10b	2.21 (*d*, *J* = 16.4)		2.21 (*d*, *J* = 14.8)	
11	–	67.7	–	67.5
12	–	207.5	–	204.8
13	–	47.8	–	47.5
14	–	203.8	–	203.3
15	–	193.4	–	192.9
16	–	135.2	–	134.8
17	7.06 (*d*, *J* = 7.2)	128.7	7.10 (*d*, *J* = 8.0)	128.4
18	7.26 (*dd*, *J* = 12.4, 8.8)	128.4	7.27 (*dd*, *J* = 7.9, 7.0)	128.1
19	7.38 (*t*, *J* = 7.6)	132.3	7.39 (*t*, *J* = 7.0)	132.1
20	7.26 (*dd*, *J* = 12.4, 8.8)	128.4	7.27 (*dd*, *J* = 7.9, 7.0)	128.1
21	7.06 (*d*, *J* = 7.2)	128.7	7.10 (*d*, *J* = 8.0)	128.4
22	1.01 (*s*)	28.6	1.03 (*s*)	28.3
23	0.94 (*s*)	20.7	0.95 (*s*)	20.5
24a	2.61 (*m*)	29.1	2.62 (*m*)	28.9
24b	2.61 (*m*)		2.62 (*m*)	
25	5.30 (*t*, *J* = 8.0)	119.2	5.30 (*t*, *J* = 7.4)	118.9
26	–	139.1	–	138.8
27	2.07 (*m*)	40.2	2.06 (*m*)	39.9
28	1.66 (*s*)	16.5	1.67 (*s*)	16.2
29	2.07 (*m*)	26.7	2.07 (*m*)	26.5
30	5.06 (*br s*)	124.4	5.07 (*br s*)	124.1
31	–	131.8	–	131.3
32	1.66 (*s*)	25.9	1.67 (*s*)	25.7
33	1.59 (*s*)	17.8	1.58 (*s*)	17.6
34	1.42 (*s*)	25.2	1.42 (*s*)	25.2
35	1.39 (*s*)	22.6	1.39 (*s*)	22.5

Table S7. ^{1}H NMR (500 MHz, δ_H, multi, (J in Hz) and ^{13}C NMR (125 MHz) data of compound **7** in acetone-d_6.

No.	**7** (acetone-d_6)		***β*-Mangostin** (acetone-d_6)	
	δ_H **(ppm)** ***J*** **(Hz)**	δ_C **(ppm)**	δ_H **(ppm)** ***J*** **(Hz)**	δ_C **(ppm)**
1	–	160.7	–	159.7
2	–	111.9	–	111.4
3	–	164.6	–	163.5
4	6.52 (1H, *s*)	89.9	6.33 (1H, *s*)	88.8
4a	–	155.6	–	155.6
5	6.87 (1H, *s*)	102.7	6.82 (1H, *s*)	101.5
6	–	156.3	–	154.4
7	–	144.6	–	142.5
8	–	138.1	–	137.0
8a	–	112.8	–	112.3
9	–	182.9	–	181.9
9a	–	103.7	–	103.7
10a	–	157.8	–	155.2
11	3.32 (2H, *d*, J = 6.6)	21.9	3.36 (2H, *d*, J = 6.8)	21.3
12	5.21 (1H, *t*, J = 6.6)	123.3	5.29 (1H, *t*, J = 6.8)	122.3
13	–	132.0	–	132.0
14	1.63 (3H, *s*)	25.9	1.85 (3H, *s*)	25.8
15	1.77 (3H, *s*)	17.8	1.82 (3H, *s*)	25.8
16	4.13 (2H, *d*, J = 6.6)	26.9	4.11 (2H, *d*, J = 6.8)	26.5
17	5.27 (1H, *t*, J = 6.6)	124.7	5.25 (1H, *t*, J = 6.8)	123.2
18	–	131.5	–	131.7
19	1.65 (3H, *s*)	25.9	1.72 (3H, *s*)	18.2
20	1.83 (3H, *s*)	18.3	1.71 (3H, *s*)	17.7
1-OH	13.67 (1H, *s*)	–	13.44 (1H, *s*)	–
6-OH	–	–	6.43 (1H, *s*)	–
3-OMe	3.97 (3H, *s*)	56.6	3.82 (3H, *s*)	55.8
7-OMe	3.80 (3H, *s*)	61.3	3.92 (3H, *s*)	62.0

Table S8. ^{1}H NMR (500 MHz, δ_H, multi, (J in Hz) and ^{13}C NMR (125 MHz) data of compound **8** in $CDCl_3$.

No.	**8** ($CDCl_3$)		***α*-Mangostin** ($CDCl_3$)	
	δ_H **(ppm)** ***J*** **(Hz)**	δ_C **(ppm)**	δ_H **(ppm)** ***J*** **(Hz)**	δ_C **(ppm)**
1	–	160.7	–	160.6
2	–	108.7	–	108.5

No.	8 (CDCl$_3$)		α-Mangostin (CDCl$_3$)	
	**δ_H (ppm) J (Hz)**	**δ_C (ppm)**	**δ_H (ppm) J (Hz)**	**δ_C (ppm)**
3	–	161.8	–	161.6
4	6.30 (1H, *s*)	93.5	6.36 (1H, *s*)	93.3
4a	–	154.7	–	154.5
5	6.83 (1H, *s*)	103.7	6.82 (1H, *s*)	102.8
6	–	155.9	–	155.8
7	–	142.7	–	142.5
8	–	137.2	–	137.0
8a	–	112.3	–	112.2
9	–	182.1	–	182.0
9a	–	101.7	–	101.6
10a	–	155.2	–	155.3
11	3.44 (2H, *d*, J = 7)	21.6	3.45 (2H, *d*, J = 6.6)	21.4
12	5.28 (1H, *t*, J = 7)	121.6	5.29 (1H, *t*, J = 6.6)	121.5
13	–	132.3	–	131.1
14	1.84 (3H, *s*)	18.4	1.84 (3H, *s*)	18.2
15	1.83 (3H, *s*)	26.0	1.83 (3H, *s*)	25.8
16	4.08 (2H, *d*, J = 6.5)	26.7	4.09 (2H, *d*, J = 6.6)	26.6
17	5.26 (1H, *t*, J = 6.5)	123.3	5.26 (1H, *t*, J = 6.6)	123.1
18	–	135.8	–	135.6
19	1.77 (3H, *s*)	18.1	1.77 (3H, *s*)	17.9
20	1.69 (3H, *s*)	26.0	1.69 (3H, *s*)	25.8
1-OH	–	–	13.78 (1H, *s*)	–
7-OCH$_3$	3.80 (3H, *s*)	62.2	3.79 (3H, *s*)	62.0

Table S9. ^{1}H NMR (500 MHz, δ_H, multi, (J in Hz) and ^{13}C NMR (125 MHz) data of compound **9** in acetone-d_6.

No.	9 (acetone-d_6)		9-Hydroxycalabaxanthone (CDCl$_3$)	
	**δ_H (ppm) J (Hz)**	**δ_C (ppm)**	**δ_H (ppm) J (Hz)**	**δ_C (ppm)**
1	–	158.8	–	157.8
2	–	105.2	–	104.4
3	–	160.7	–	nd
4	6.26 (1H, *s*)	94.6	6.24 (1H, *s*)	94.0
4a	–	157.2	-	156.1
5	6.87 (1H, *s*)	102.9	6.83 (1H, *s*)	101.6
6	–	158.8	–	154.5

No.	9 (acetone-d_6)		9-Hydroxycalabaxanthone ($CDCl_3$)	
	δ_H (ppm) J (Hz)	δ_C (ppm)	δ_H (ppm) J (Hz)	δ_C (ppm)
7	–	144.8	–	142.7
8	–	138.2	–	136.9
8a	–	111.8	–	112.1
9	–	183.1	–	181.8
9a	–	104.5	–	103.6
10	–	–	–	–
10a	–	157.2	–	155.6
11	6.68 (1H, *d*, J = 10.0)	116.0	6.73 (1H, *d*, J = 9.9)	115.6
12	5.72 (1H, *d*, J = 10.0)	125.5	5.57 (1H, *d*, J = 9.9)	126.9
13	–	78.8	–	77.8
14	1.46 (3H, *s*)	28.5	1.46 (3H, *s*)	28.3
15	1.46 (3H, *s*)	28.5	1.46 (3H, *s*)	28.3
16	4.12 (2H, *d*, J = 6.5)	26.8	4.09 (2H, *d*, J = 6.6)	26.5
17	5.26 (1H, *t*, J = 6.5)	124.6	5.27 (1H, *t*, J = 6.6)	123.1
18	–	131.6	–	131.8
19	1.82 (3H, *s*)	18.3	1.83 (3H, *s*)	18.1
20	1.65 (3H, *s*)	25.9	1.70 (3H, *s*)	25.6
1-OH	13.93 (1H, *s*)	–	13.70 (1H, *s*)	–
6-OH	–	–	–	–
7-OCH_3	3.80 (3H, *s*)	61.3	3.81 (3H, *s*)	61.8

Table S10. ^{1}H NMR (500 MHz, δ_H, multi, (J in Hz) and ^{13}C NMR (125 MHz) data of compound **10** in acetone-d_6.

No.	10 (acetone-d_6)		Fuscaxanthone A (acetone-d_6)	
	δ_H (ppm) J (Hz)	δ_C (ppm)	δ_H (ppm) J (Hz)	δ_C (ppm)
1	–	158.9	–	157.9
2	–	104.7	–	104.5
3	–	160.7	–	159.8
4	6.26 (1H, *s*)	94.7	6.24 (1H, *s*)	94.1
4a	–	157.2	–	156.2
5	6.87 (1H, *s*)	102.9	6.83 (1H, *s*)	101.6
6	–	156.3	–	154.5
7	–	142.1	–	142.7
8	–	138.3	–	137.0
8a	–	112.0	–	112.2

No.	10 (acetone-d_6)		Fuscaxanthone A (acetone-d_6)	
	δ_H (ppm) J (Hz)	δ_C (ppm)	δ_H (ppm) J (Hz)	δ_C (ppm)
9	–	182.5	–	181.9
9a	–	103.6	–	103.7
10	–	–	–	–
10a	–	157.8	–	155.7
11	6.68 (1H, *d*, J = 10.0)	116.0	6.72 (1H, *d*, J = 10.0)	115.7
12	5.72 (1H, *d*, J = 10.0)	128.5	5.56 (1H, *d*, J = 10.0)	127.1
13	–	78.3	–	77.9
14	1.46 (3H, *s*)	28.5	1.46 (3H, *s*)	28.3
15	1.46 (3H, *s*)	28.5	1.46 (3H, *s*)	28.3
16	4.13 (2H, *d*, J = 6.5)	26.8	4.09 (2H, *d*, J = 6.5)	26.5
17	5.28 (1H, *m*)	124.7	5.26 (1H, *m*)	123.2
18	–	135.3	–	135.6
19	1.98 (2H, *m*)	40.5	2.01 (2H, *m*)	39.7
20	2.05 (2H, *m*)	27.3	2.04 (2H, *m*)	26.4
21	5.04 (1H, *m*)	125.2	5.02 (1H, *m*)	124.2
22	–	131.6	–	131.2
23	1.52 (3H, *s*)	17.8	1.54 (3H, *s*)	17.6
24	1.84 (3H, *s*)	16.5	1.82 (3H, *s*)	16.5
25	1.56 (3H, *s*)	25.7	1.60 (3H, *s*)	25.6
1-OH	13.93 (1H, *s*)	–	13.55 (1H, *s*)	–
6-OH	9.71 (1H, *s*)	–	6.15 (1H, *s*)	–
7-OCH_3	3.80 (3H, *s*)	61.4	3.73 (3H, *s*)	62.0

Table S11. ^{1}H NMR (500 MHz, δ_H, multi, (J in Hz) and ^{13}C NMR (125 MHz) spectral data comparison of compound **11** in acetone-d_6.

No.	11 (acetone-d_6)		11-hydroxy-1-isomangostin (acetone-d_6)	
	δ_H (ppm) J (Hz)	δ_C (ppm)	δ_H (ppm) J (Hz)	δ_C (ppm)
1	–	156.0	–	155.9
2	–	105.5	–	105.0
3	–	161.2	–	161.0
4	6.39 (1H, *s*)	94.1	6.40 (1H, *s*)	94.2
4a	–	157.6	–	157.6
4b	–	154.9	–	154.9
5	6.71 (1H, *s*)	102.1	6.72 (1H, *s*)	102.1
6	–	155.6	–	155.8

No.	11 (acetone-d_6)		11-hydroxy-1-isomangostin (acetone-d_6)	
	δ_H (ppm) J (Hz)	δ_C (ppm)	δ_H (ppm) J (Hz)	δ_C (ppm)
7	–	144.4	–	144.3
8	–	137.7	–	137.6
8a	–	115.4	–	115.1
9	–	176.1	–	176.3
9a	–	107.9	–	107.7
10	2.57 (1H, *dd*, J = 16.8, 7.8)<br>2.92 (1H, *dd*, J = 16.8, 5.8)	27.1	2.56 (1H, *dd*, J = 16.8, 7.8)<br>2.94 (1H, *dd*, J = 16.8, 5.7)	27.1
11	3.81 (1H, *dd*, J = 8, 5.5)	69.1	3.82 (1H, *dd*, J = 7.8, 5.7)	69.0
12	–	78.6	–	78.5
13	1.30 (3H, *s*)	20.6	1.30 (3H, *s*)	20.6
14	1.43 (3H, *s*)	26.0	1.43 (3H, *s*)	26.0
15	4.09 (2H, *t*, J = 6)	26.6	4.09 (2H, *d*, J = 6.8)	26.6
16	5.31 (1H, *t*, J = 6.8)	125.6	5.31 (1H, *br t* , J = 6.8)	125.8
17	–	130.2	–	130.5
18	1.82 (3H, *s*)	26.1	1.81 (3H, *br s*)	26.1
19	1.65 (3H, *s*)	18.3	1.64 (3H, *br s*)	18.3
7-OMe	3.77 (3H, *s*)	61.2	3.77 (3H, *s*)	61.0

Table S12. Alpha-glucosidase inhibition (IC_{50}) by extracts of studied plant

**Bio-source**		**IC_{50}**
**Extract**	Crude MeOH	27.7 ± 0.5 µg/mL
	Extract **H**	>200 µg/mL
	Extract **EA**	66.0 ± 2.6 µg/mL
**Fraction**	Fractions **EA1-EA5**	>200 µg/mL
	Fraction **EA6**	12.0 ± 0.4 µg/mL
	Fractions **EA7-EA11**	>200 µg/mL
	Fraction **EA12**	60.9 ± 1.2 µg/mL
	Fraction **EA13**	>200 µg/mL
	Fraction **EA14**	>200 µg/mL
	Fraction **EA15**	66.9 ± 3.7 µg/mL
	Fraction **EA16**	155.9 ± 4.4 µg/mL

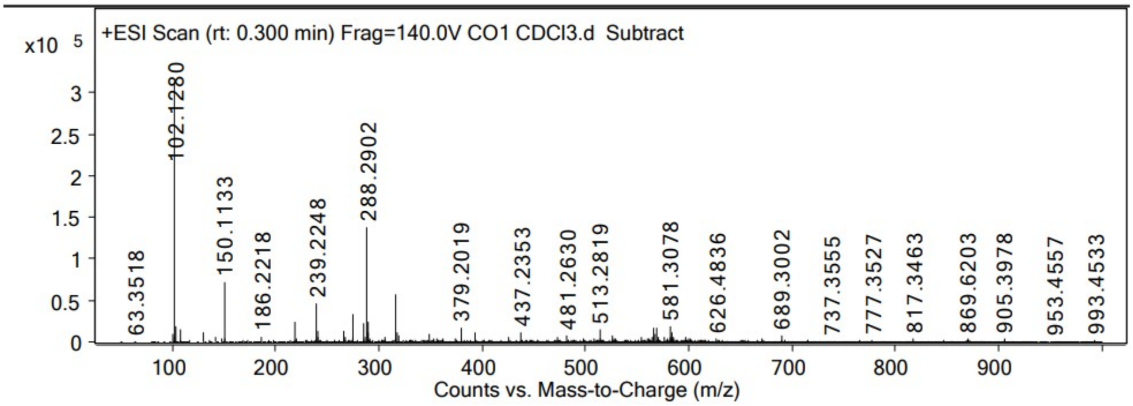

Qualitative Analysis Report

535.34		505.45
535.4401	1	464
535.4724	1	285.22
536.2408		316.57
536.3064		482.45
536.355		367.71
537.2214		389.98
537.2855		258.96
537.3175	1	1644.98
537.4289		211.36
538.226		408.76
538.3198	1	753.27
538.3705		501.25
538.4315	1	2609.87
538.61		324.96
538.9374		213.65
539.1318		204.8
539.2621		864.14
539.2991	1	1120.32
539.3286		724.5
539.3704		451.54
539.4331	1	662.92
540.2837		838.01
540.334		1008.27
540.4314	1	805.96
540.5389		589.08
540.9295		249.86
541.1746		310.72
541.2671	1	1580.1
541.3271		1031.17
541.443	1	374.14
542.2172		233.78
542.2752	1	796.59
542.3474		494.92
542.3849		428.57
543.1245		324.05
543.3059		1136.04
543.4426		218.7
543.5453		244.94
544.2202		207.88
544.2698		375.34
544.3406	1	614.06
544.4136	1	556.6
545.2855	1	875
545.3467	1	270.47
545.4182	1	279.34

Agilent Technologies | Page 26 of 54 | Printed at 2:48 PM on 25-Feb-2022

User Spectrum Plot Report

Agilent Trusted Answers

Name	GA A9	Rack Pos.		Instrument	6545B	Operator	
Inj. Vol. (ul)	3	Plate Pos.		IRM Status	All ions missed		
Data File	GA A9.d	Method (Acq)	CO TUYEN 16112023 GAA 09.m	Comment		Acq. Time (Local)	17/11/2023 6:37:36 PM (UTC+07:00)

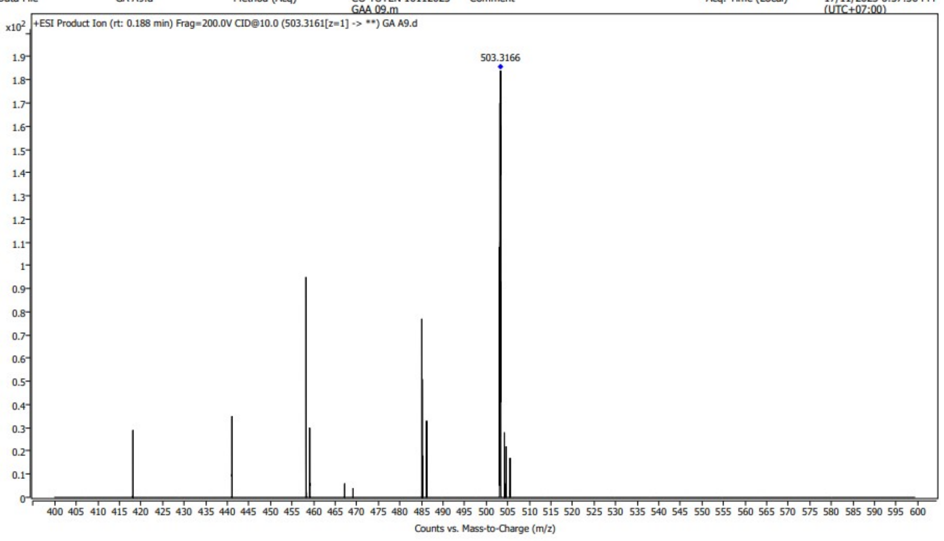

Page 1 of 1

Generated at 10:05 AM on 18/11/2023

Figure S1A. The HRESIMS spectrum of **1.**

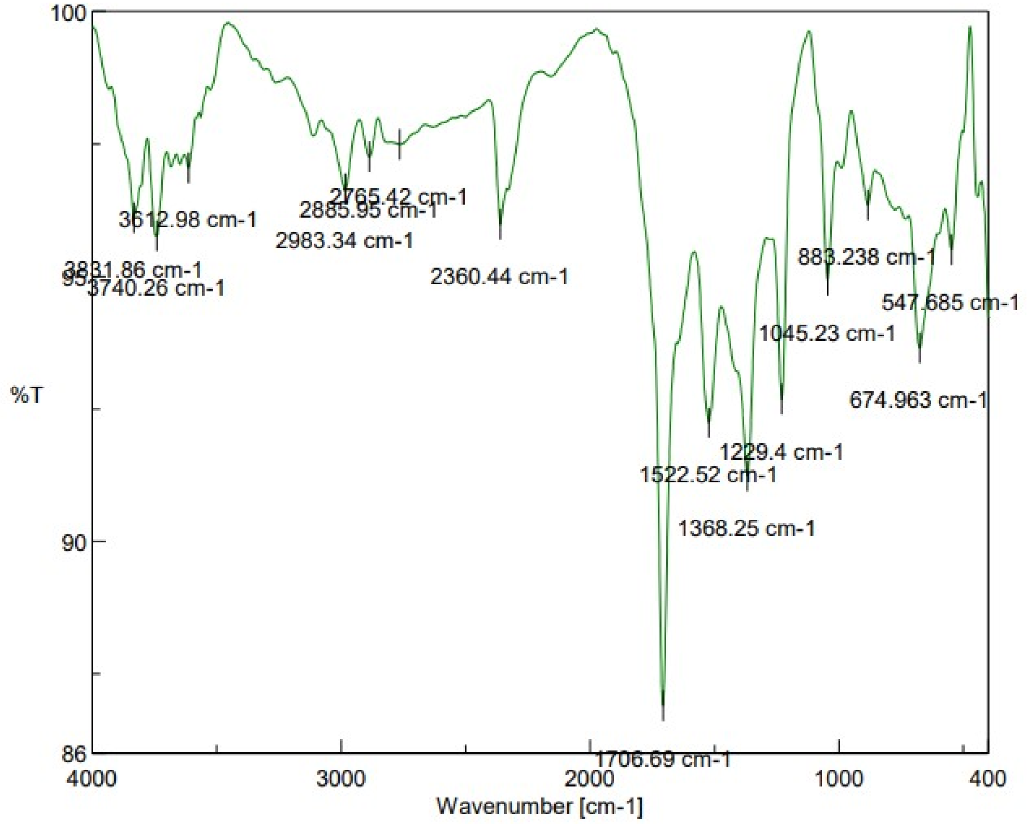

Figure S1B. The IR spectrum of **1.**

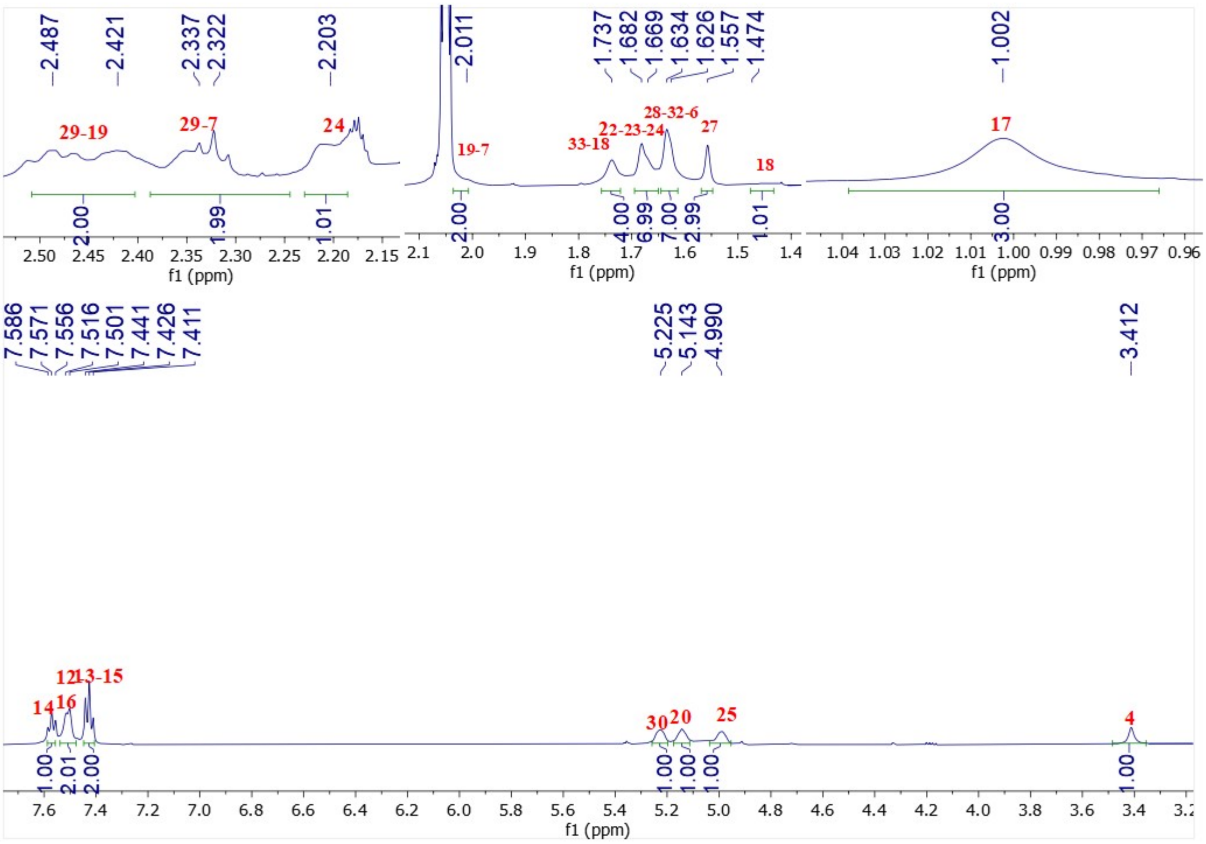

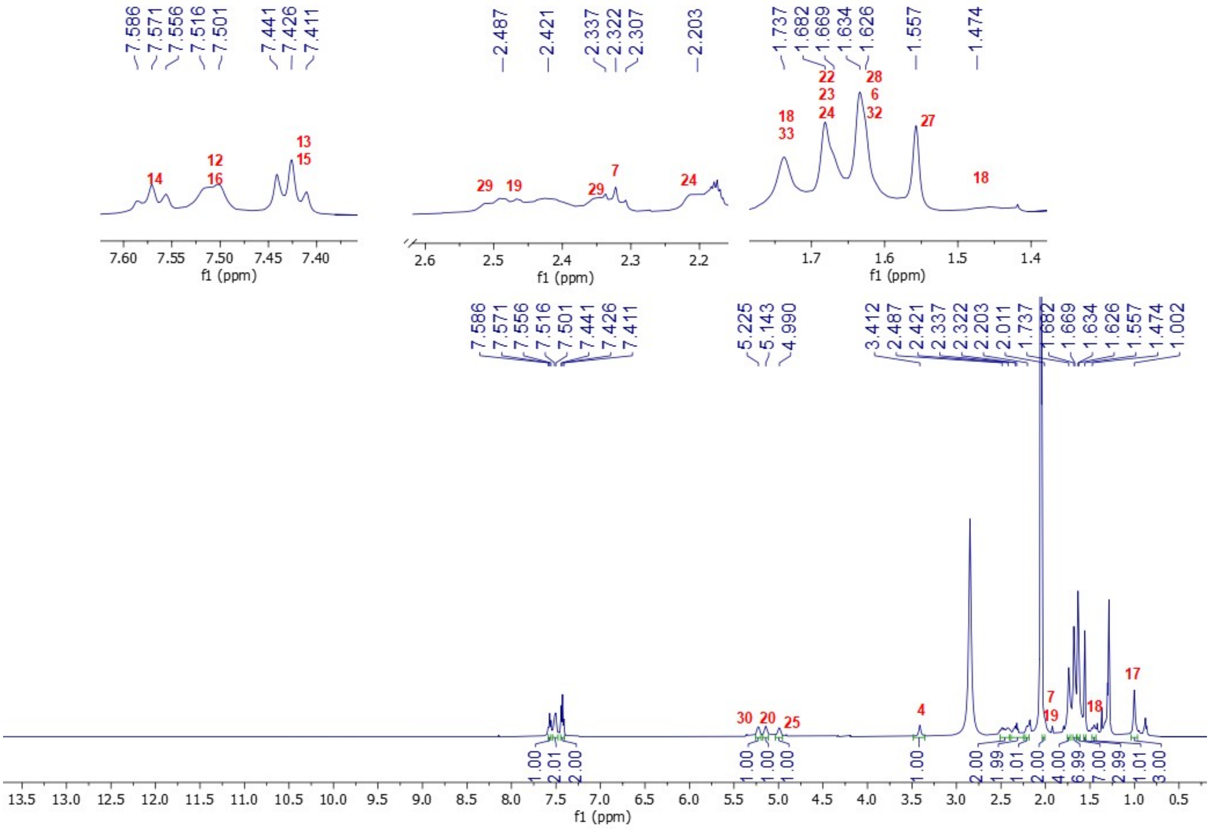

Figure S1C. The ^{1}H NMR spectrum of **1** in acetone-d_6.

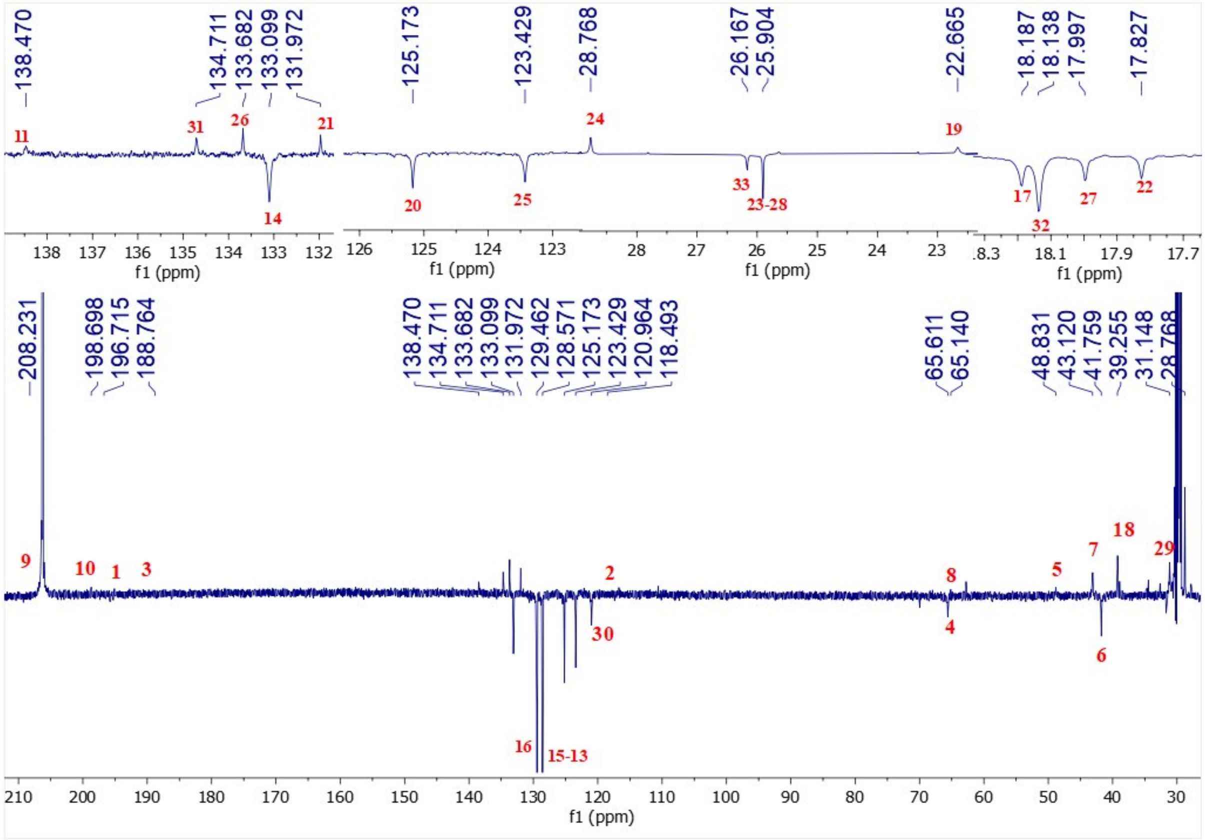

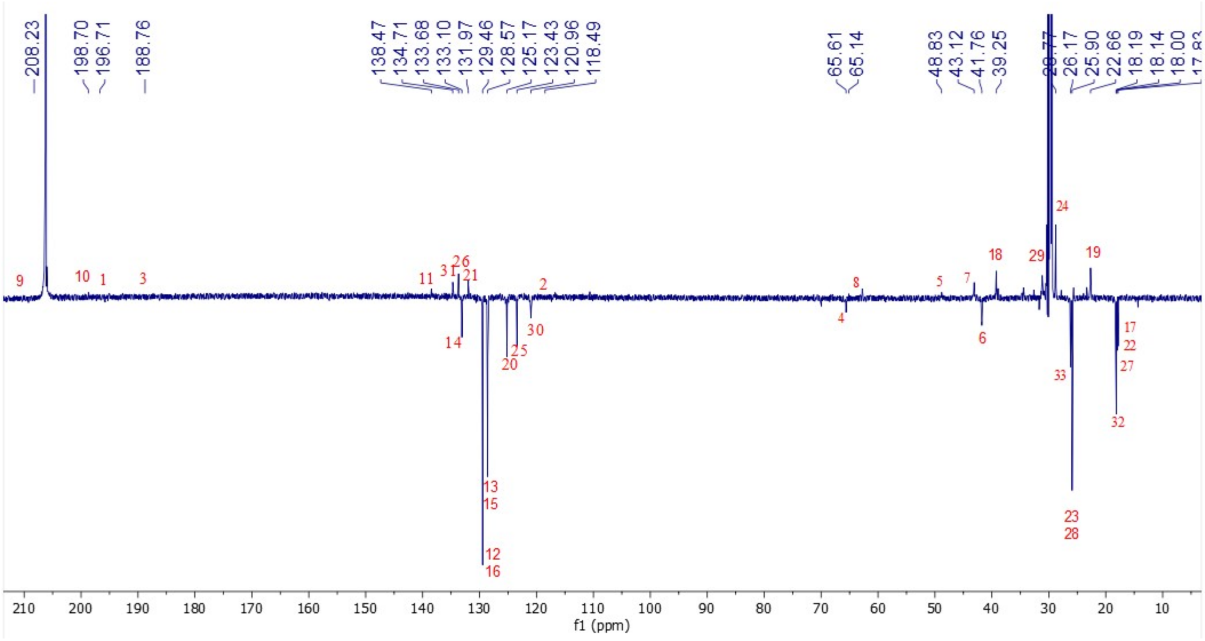

Figure S1D. The ^{13}C NMR spectrum of **1** in acetone-d_6.

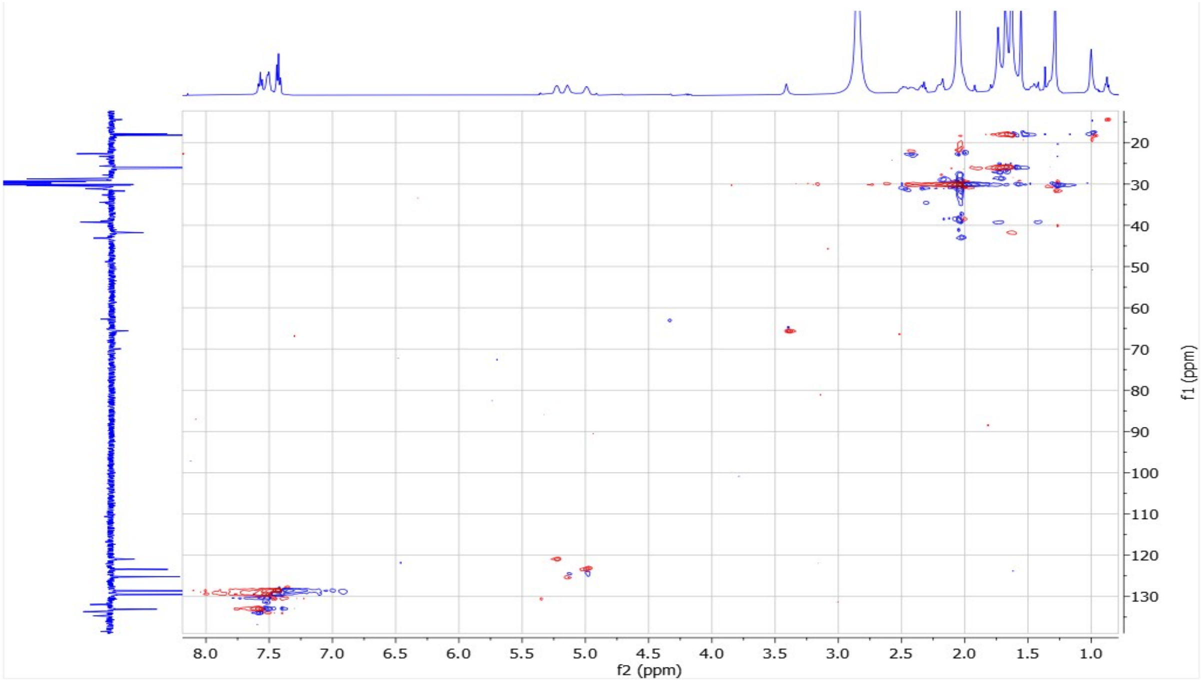

Figure S1E. The HSQC spectrum of **1** in acetone-d_6.

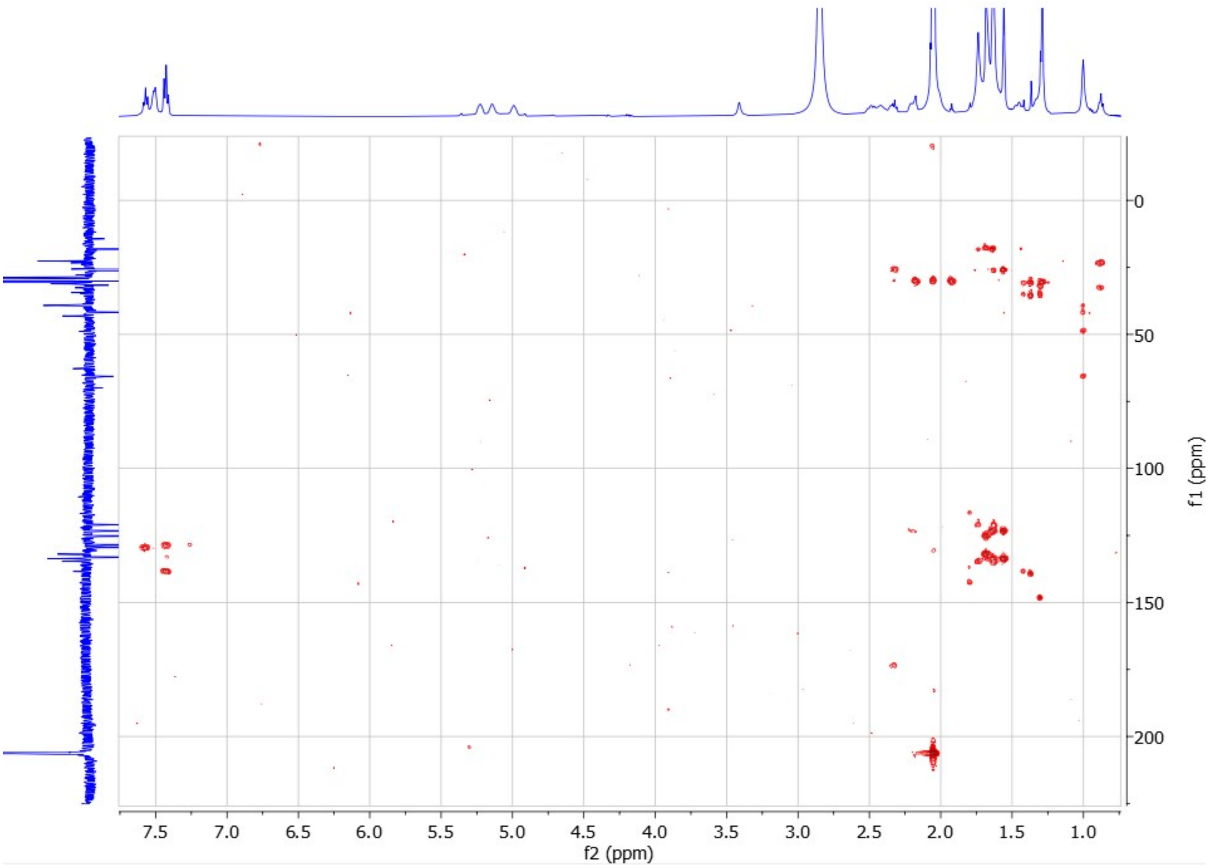

Figure S1F. The HMBC spectrum of **1** in acetone-d_6.

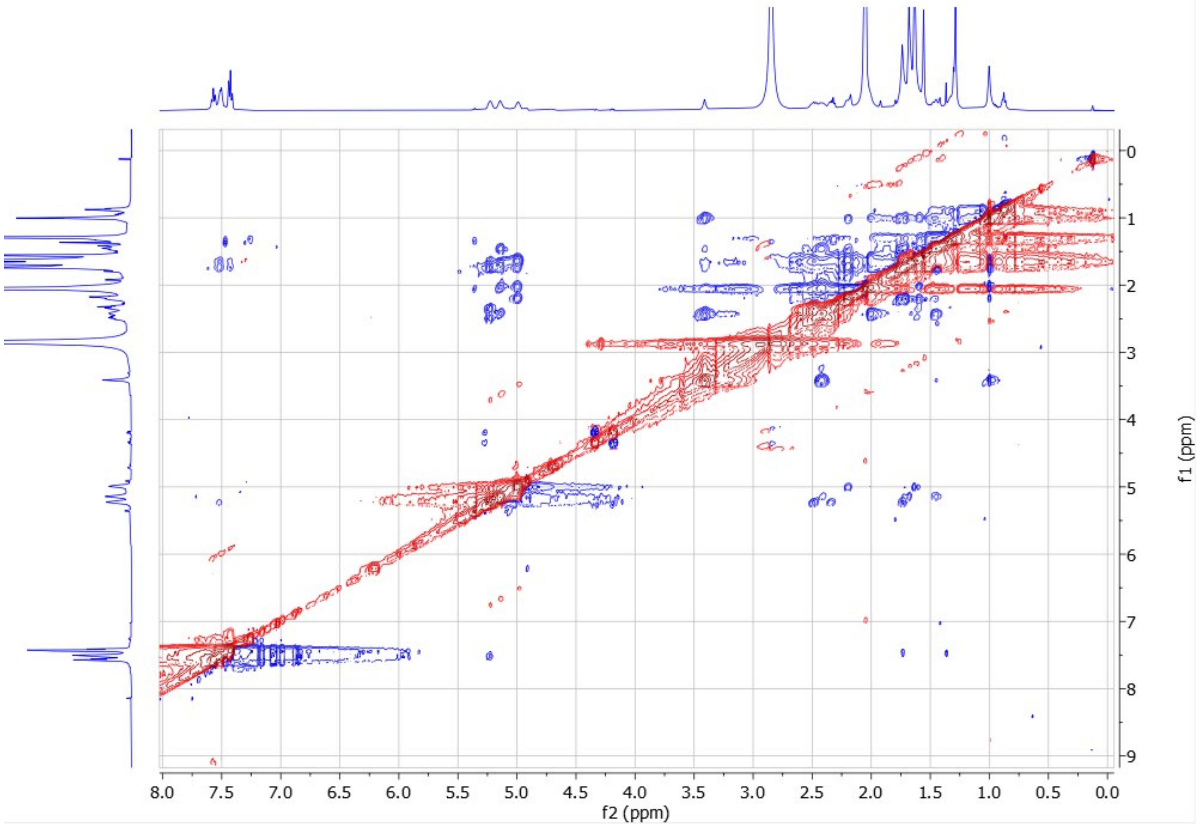

Figure S1G. The NOESY spectrum of **1** in acetone-d_6.

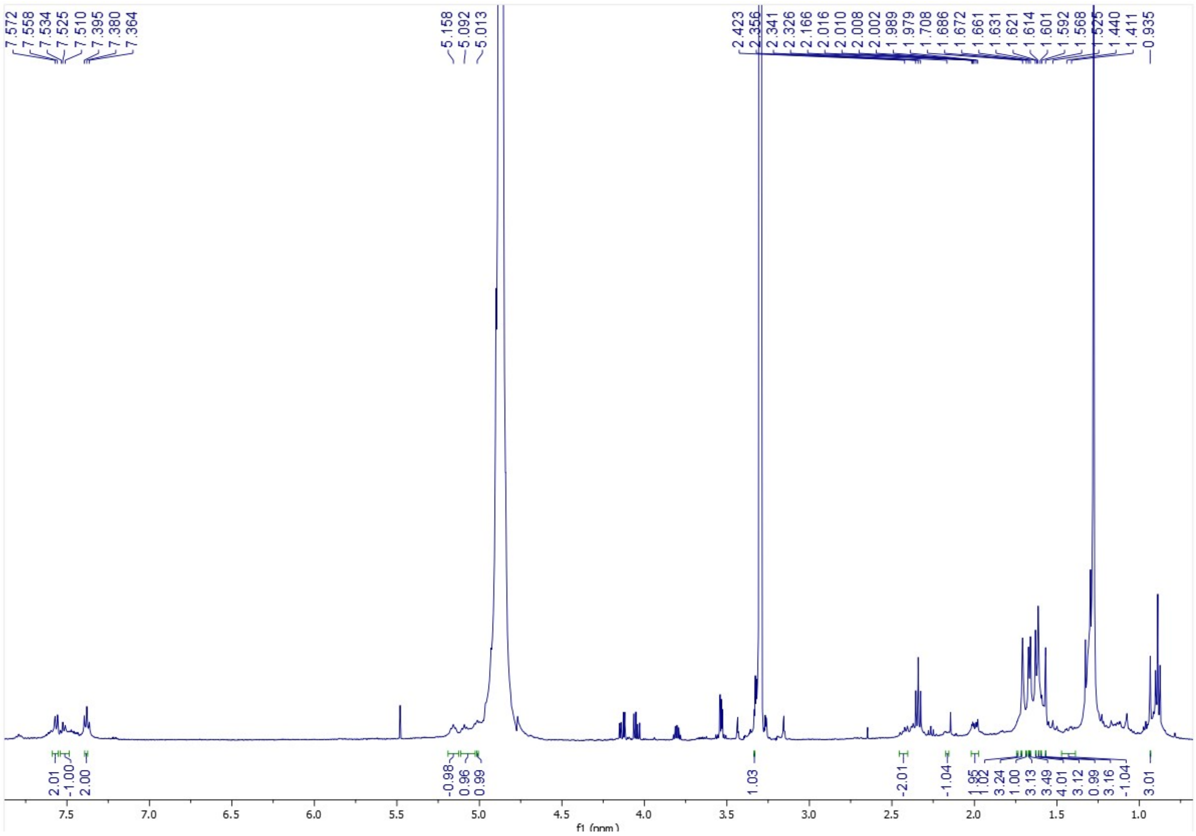

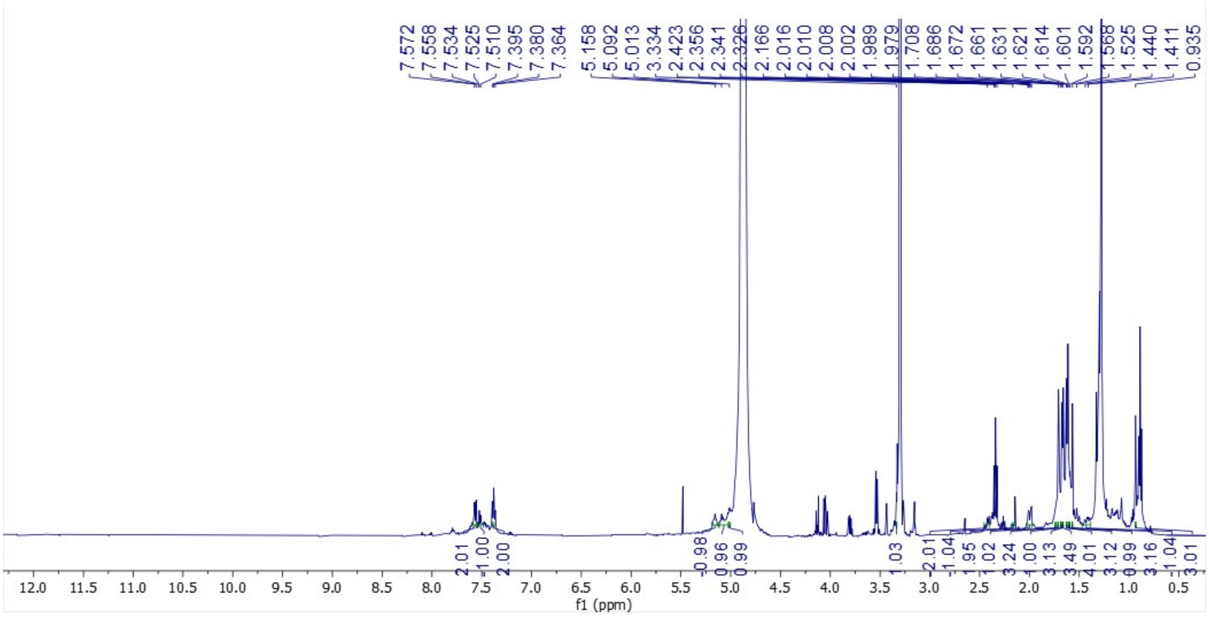

Figure S1H. The ^{1}H NMR spectrum of **1** in methanol-d_4.

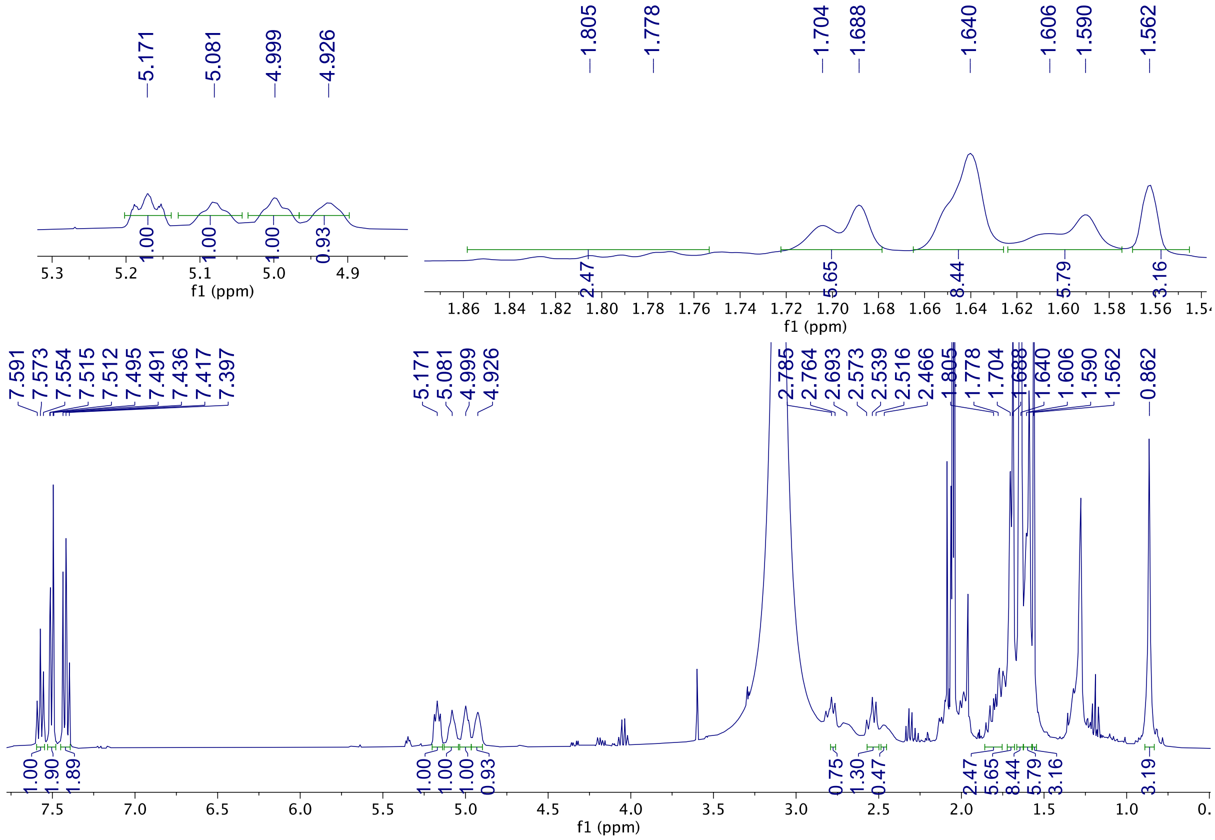

Figure S2A. The ^{1}H NMR spectrum of **2** in acetone-d_6.

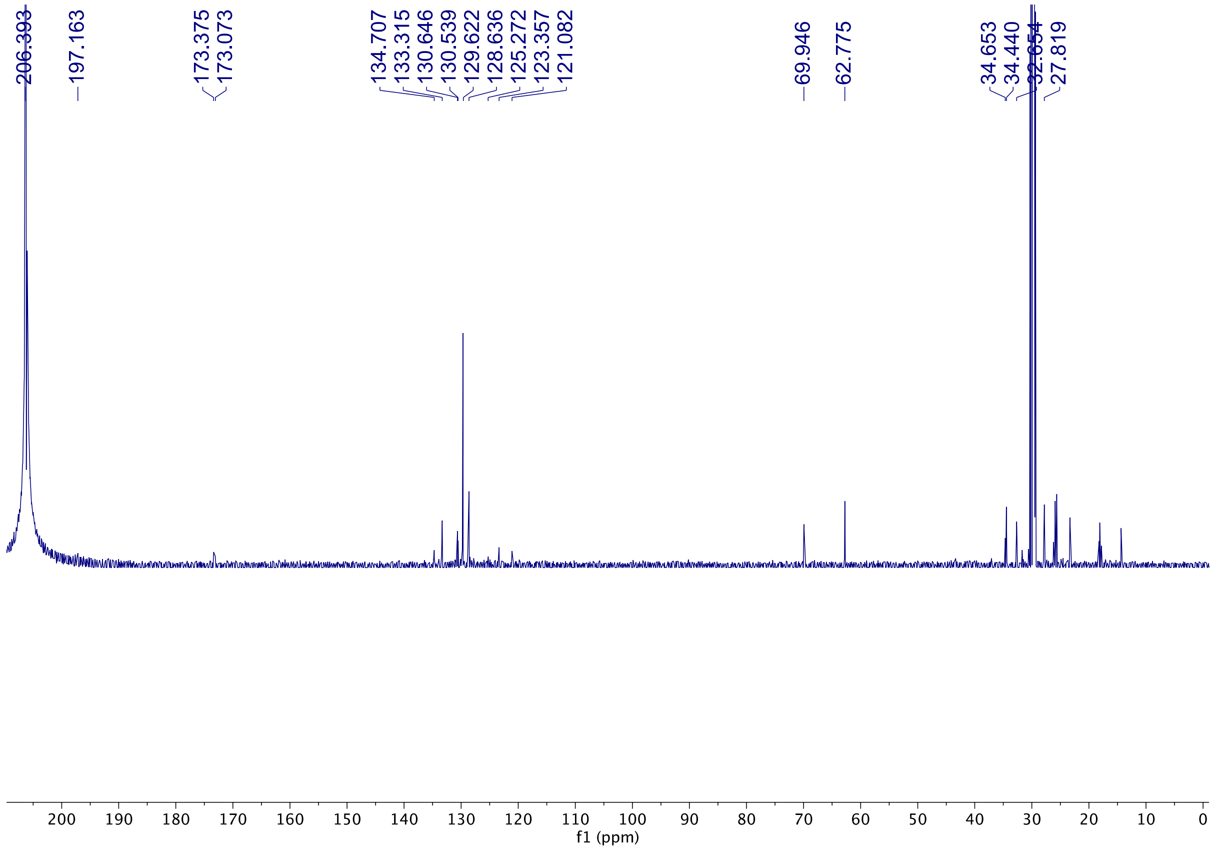

Figure S2B. The ^{13}C NMR spectrum of **2** in acetone-d_6.

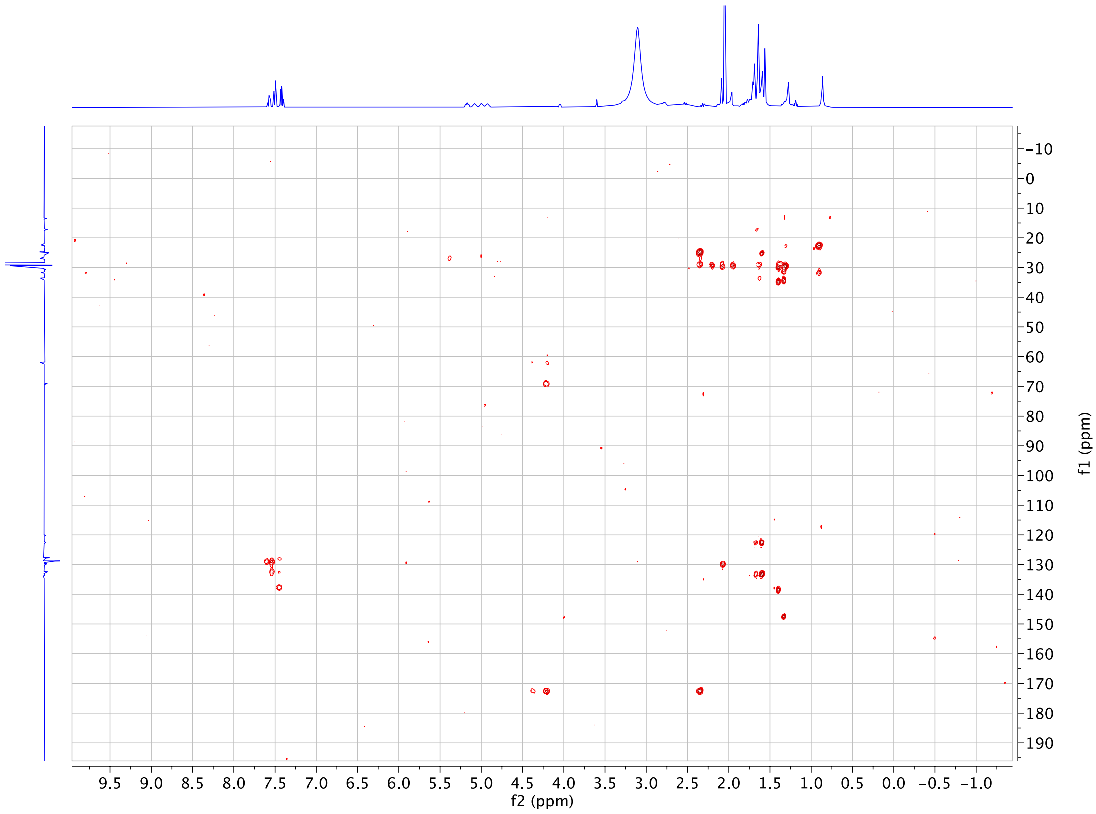

Figure S2C. The HMBC spectrum of **2** in acetone-d_6.

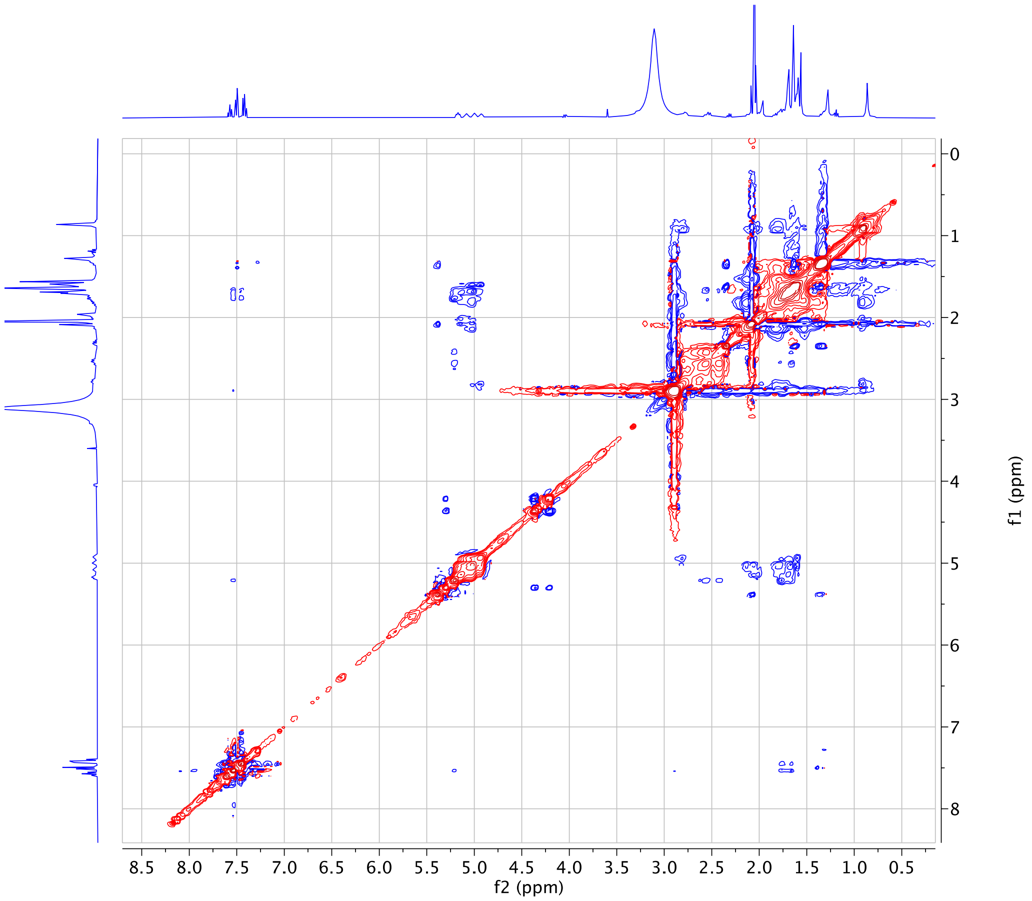

Figure S2D. The NOESY spectrum of **2** in acetone-d_6.

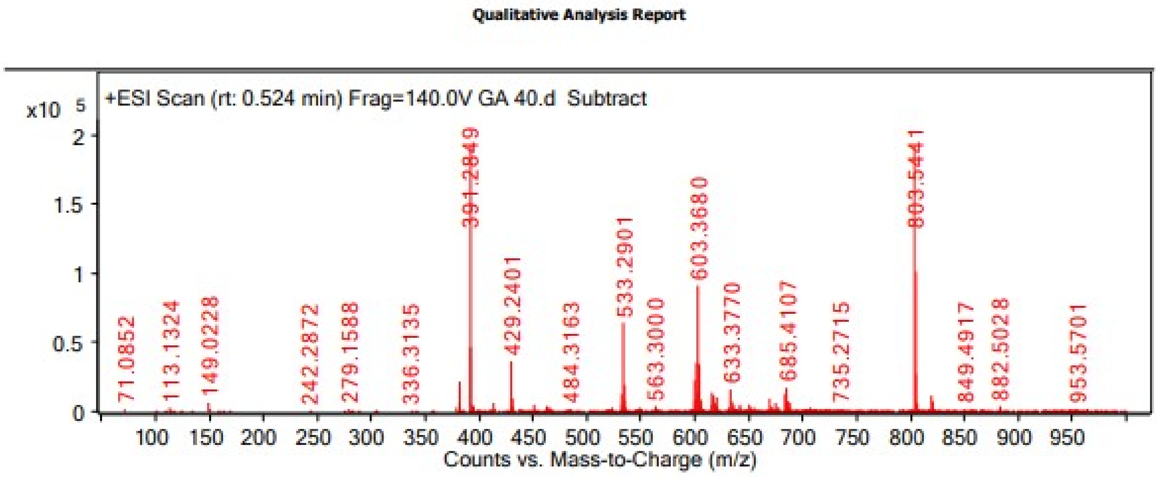

Figure S3A. The HRESIMS spectrum of **3.**

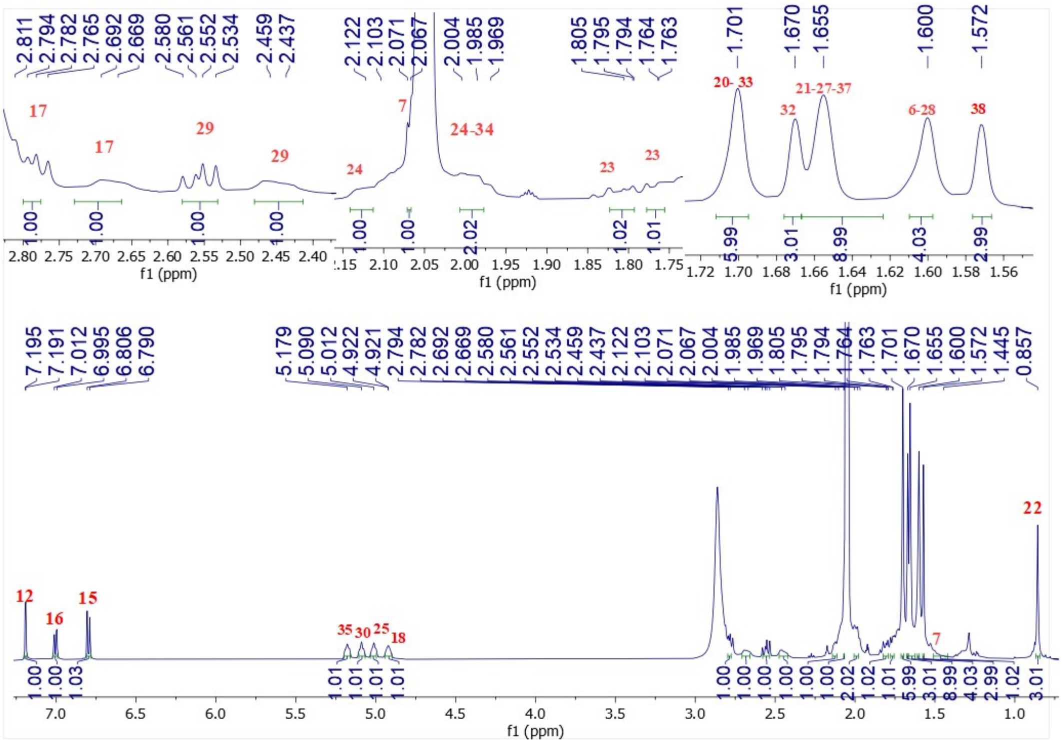

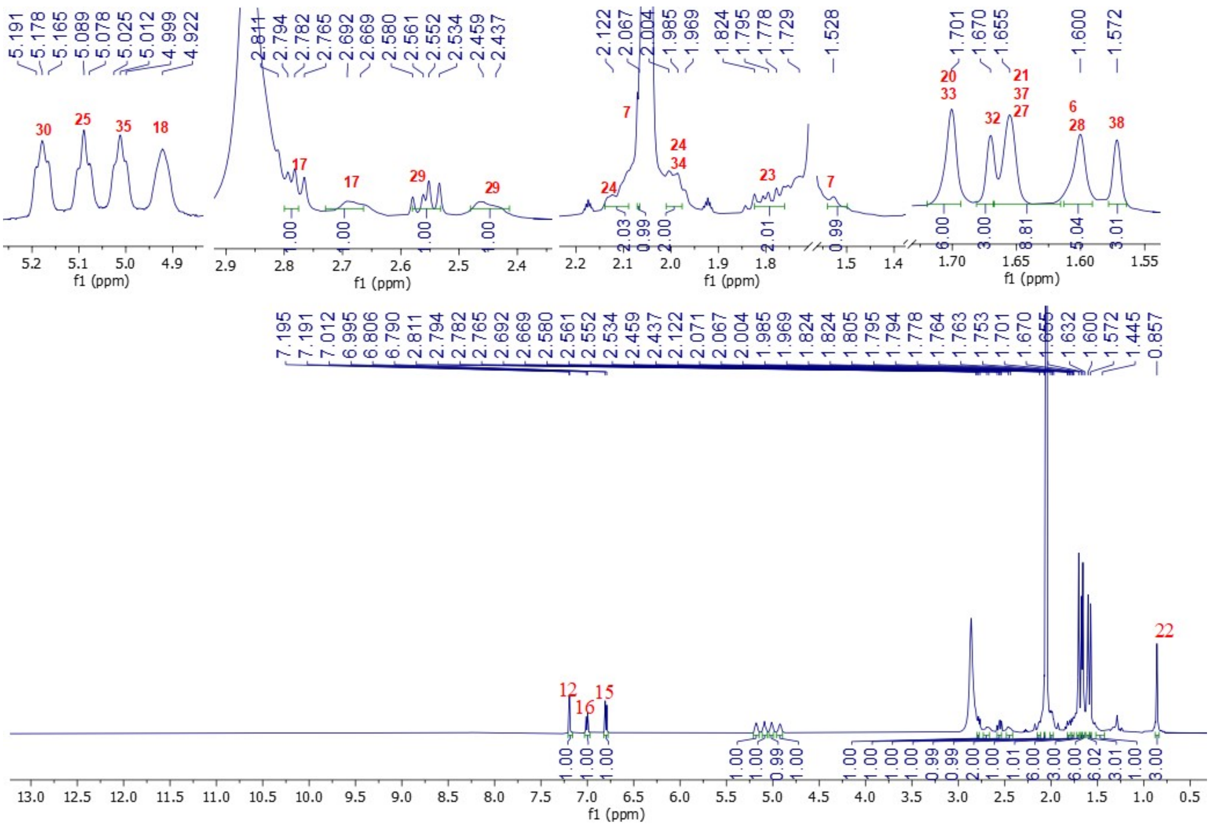

Figure S3B. The ^{1}H NMR spectrum of **3** in acetone-d_6.

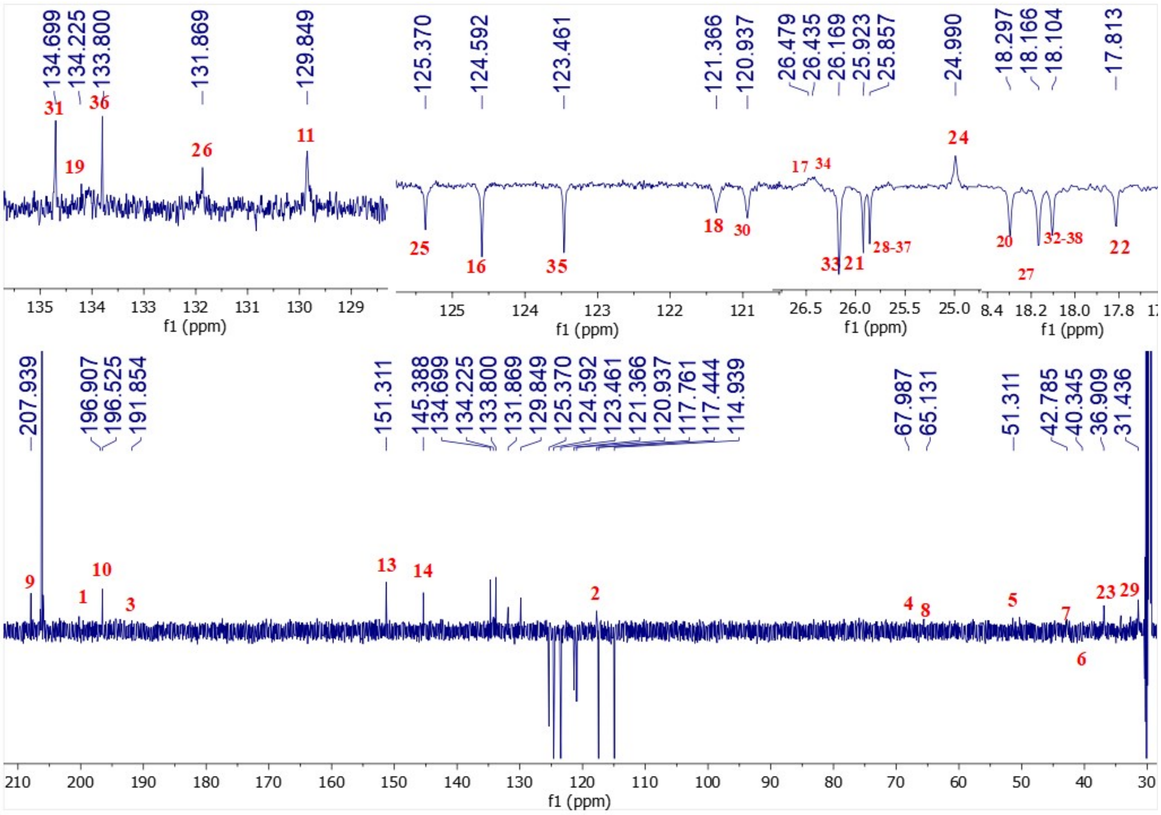

Figure S3C. The ^{13}C NMR spectrum of **3** in acetone-d_6.

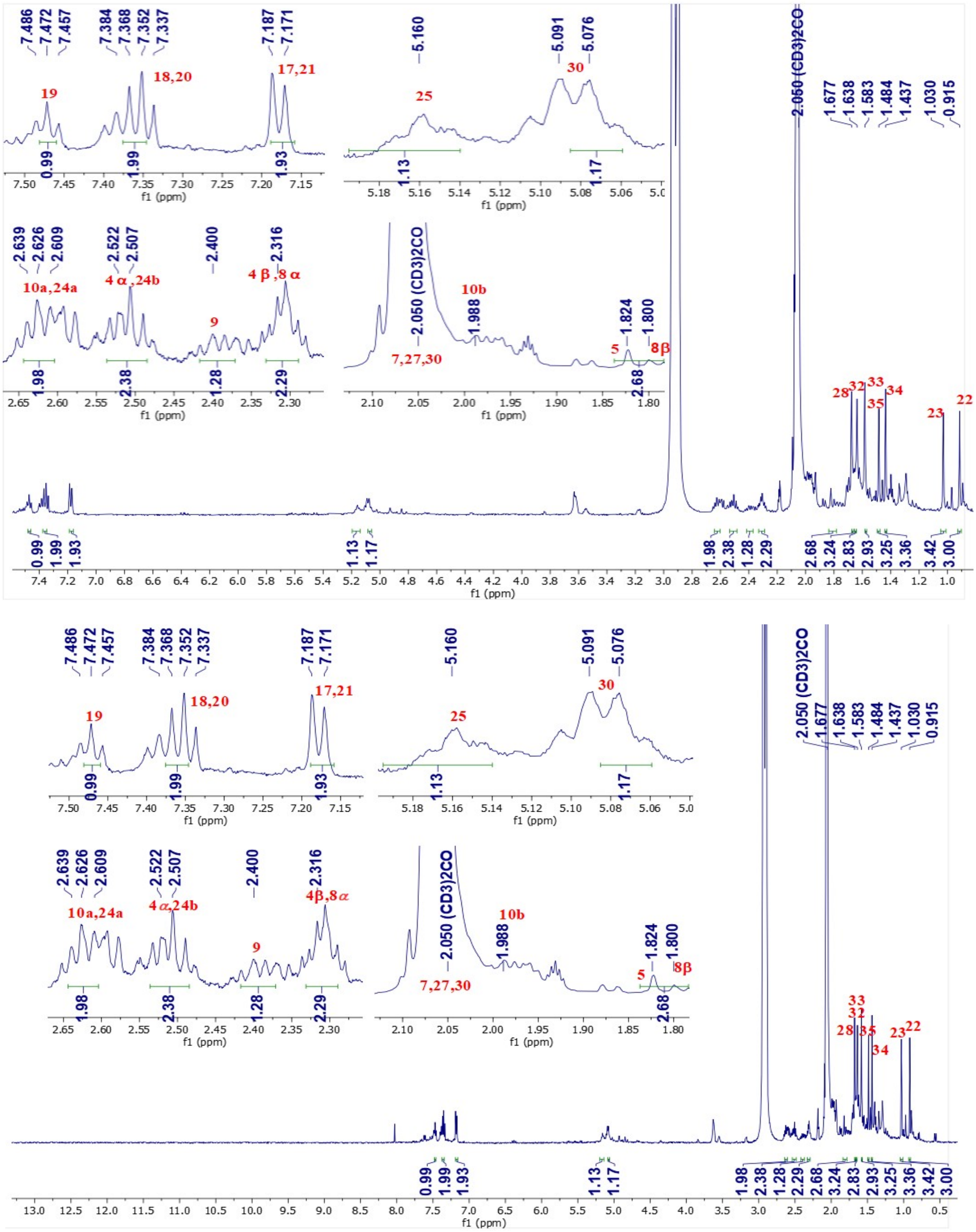

Figure S4A. The ^{1}H NMR spectrum of **4** in acetone-d_6.

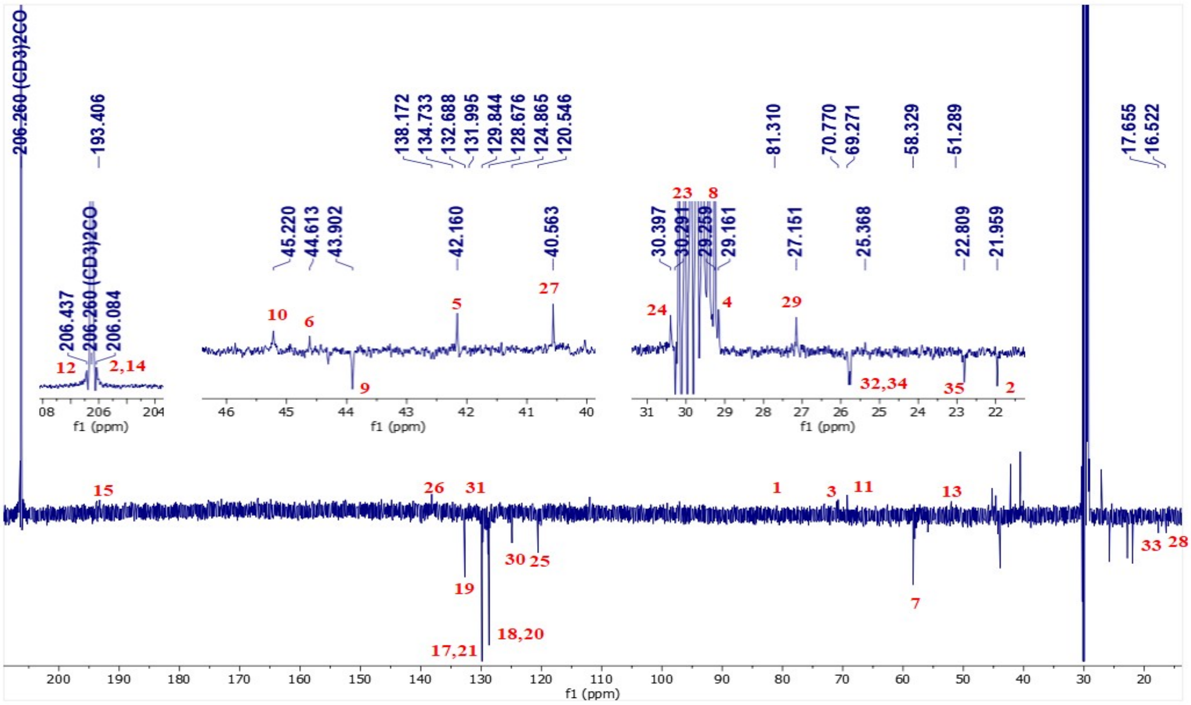

Figure S4B. The ^{13}C NMR spectrum of **4** in acetone-d_6.

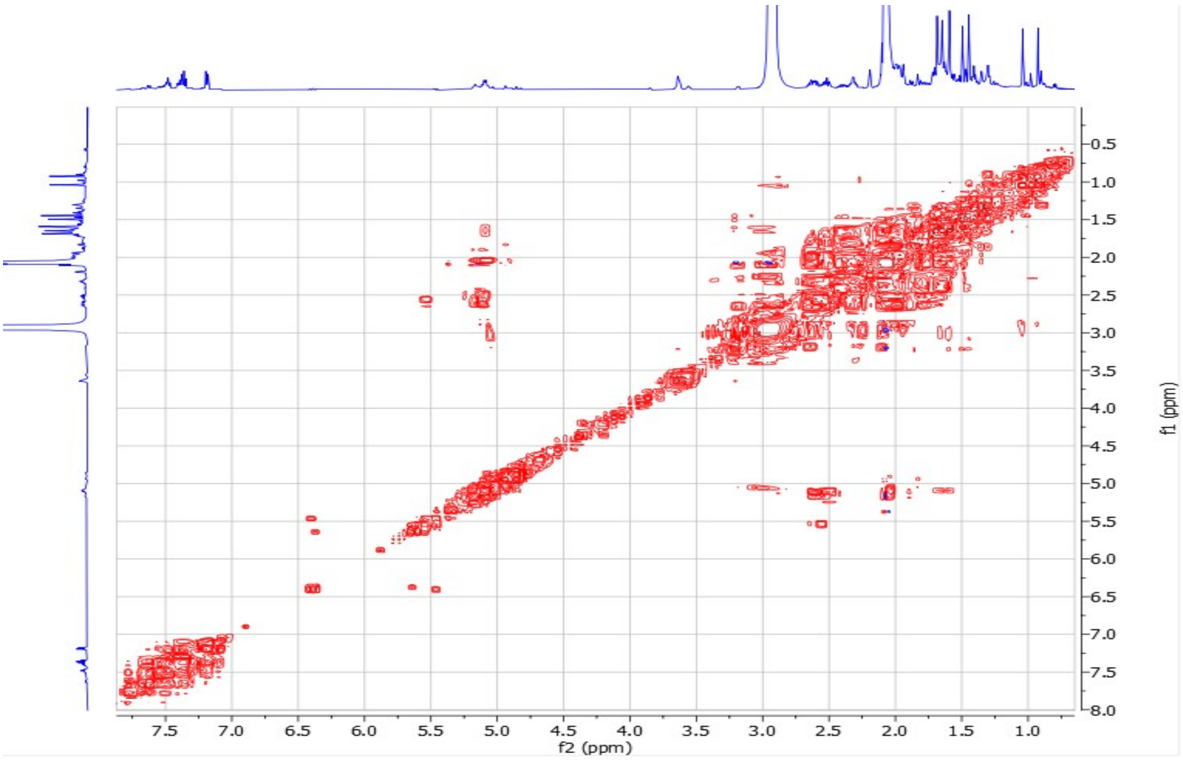

Figure S4C. The COSY spectrum of **4** in acetone-d_6.

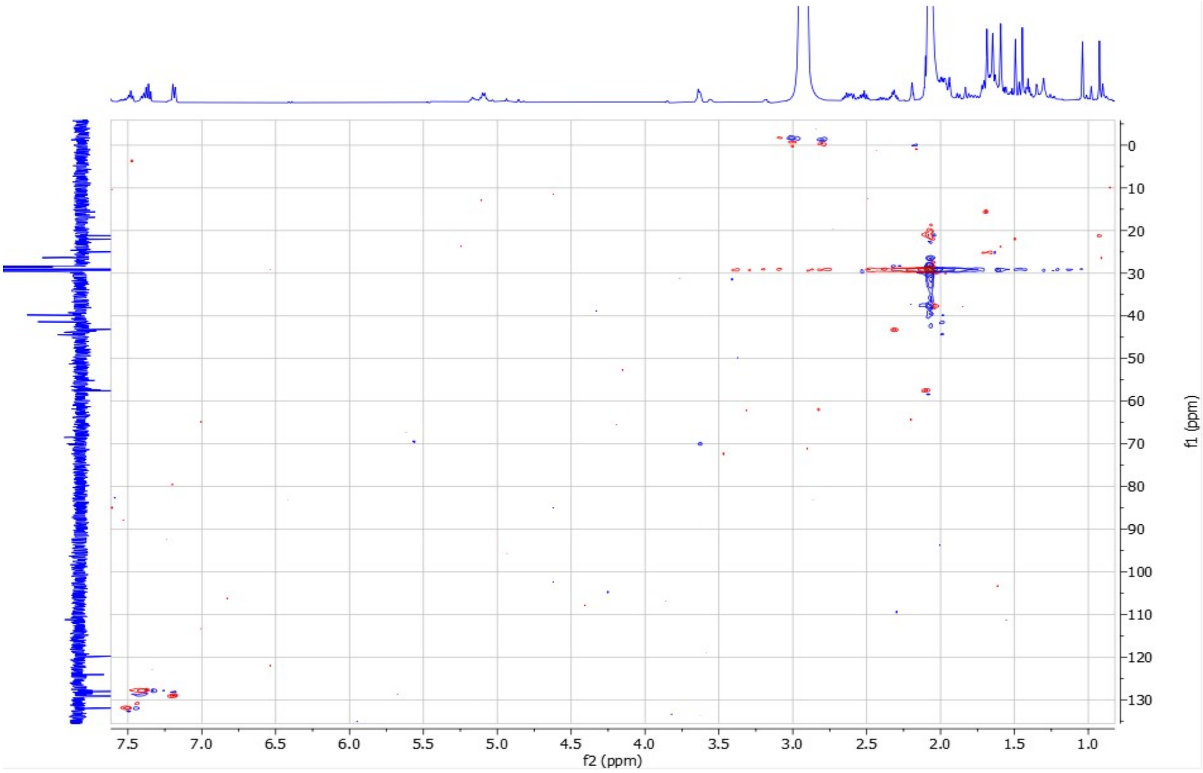

Figure S4D. The HSQC spectrum of **4** in acetone-d_6.

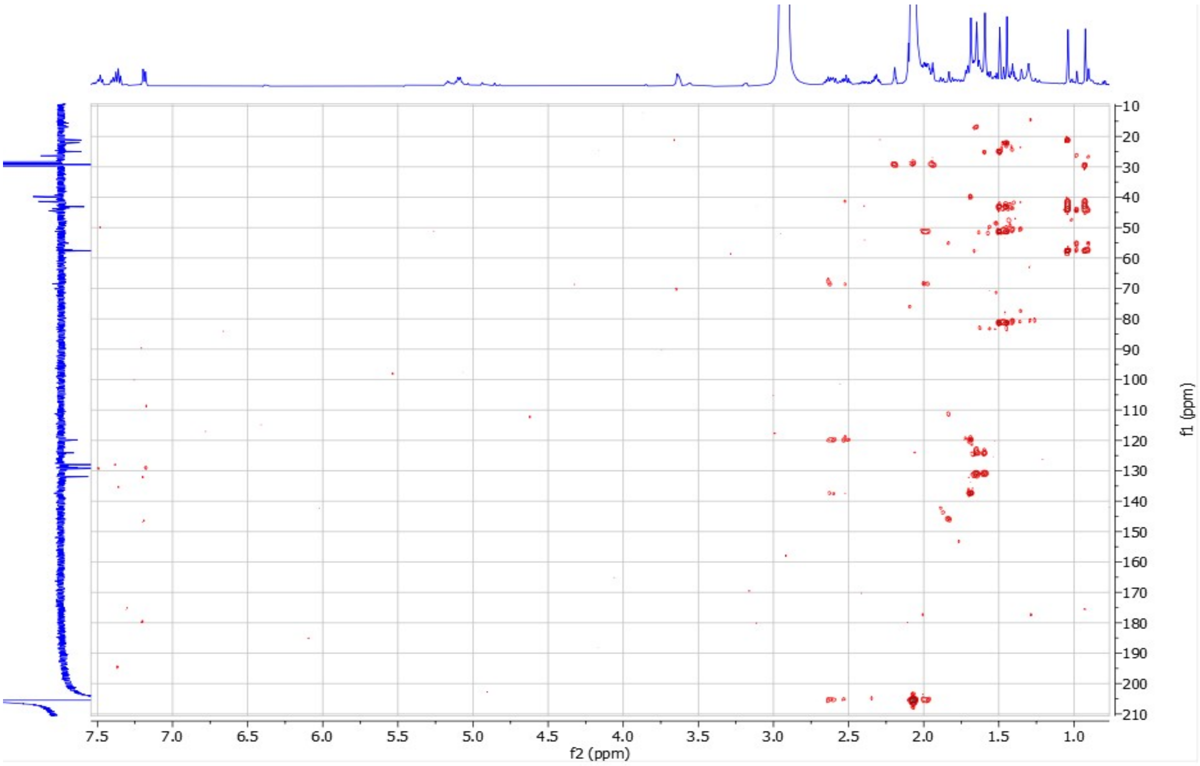

Figure S4E. The HMBC spectrum of **4** in acetone-d_6.

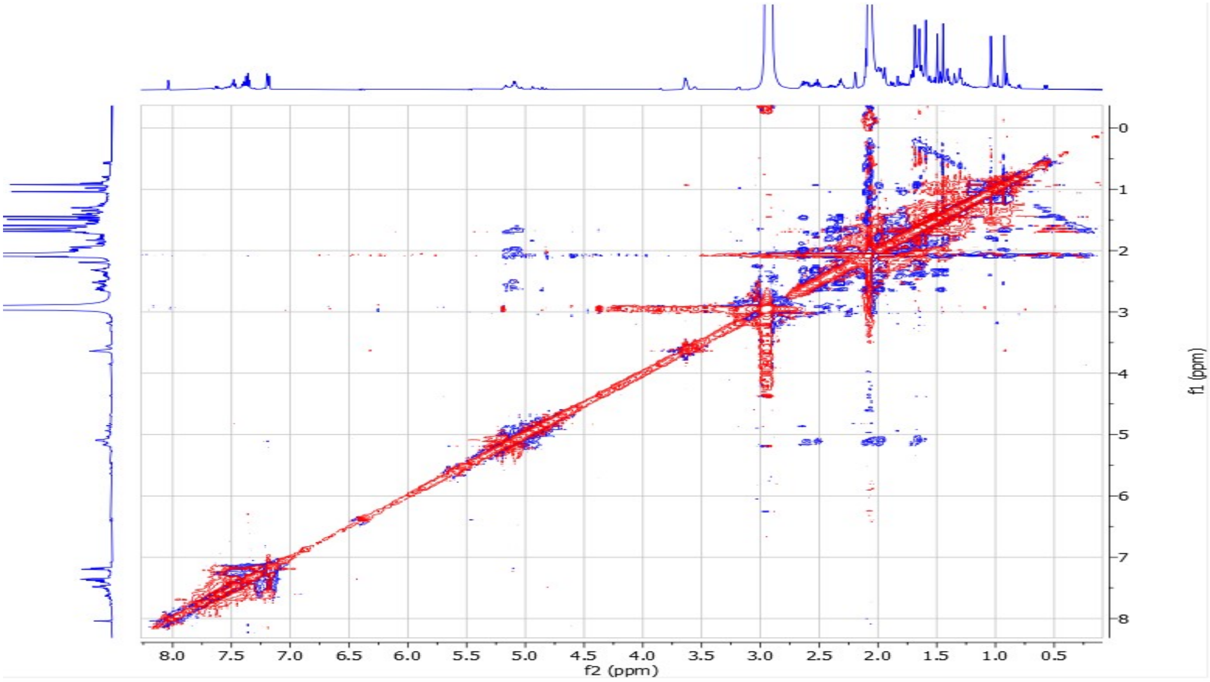

Figure S4F. The NOESY spectrum of **4** in acetone-d_6.

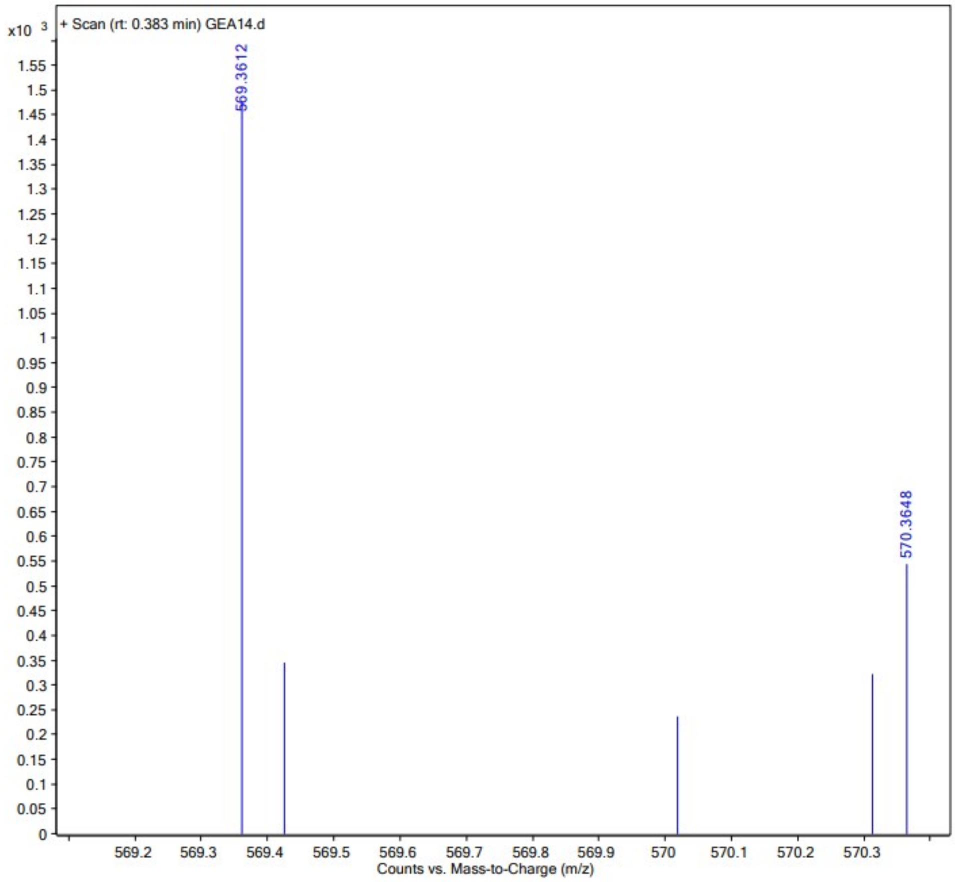

Figure S5A. The HRESIMS spectrum of **5.**

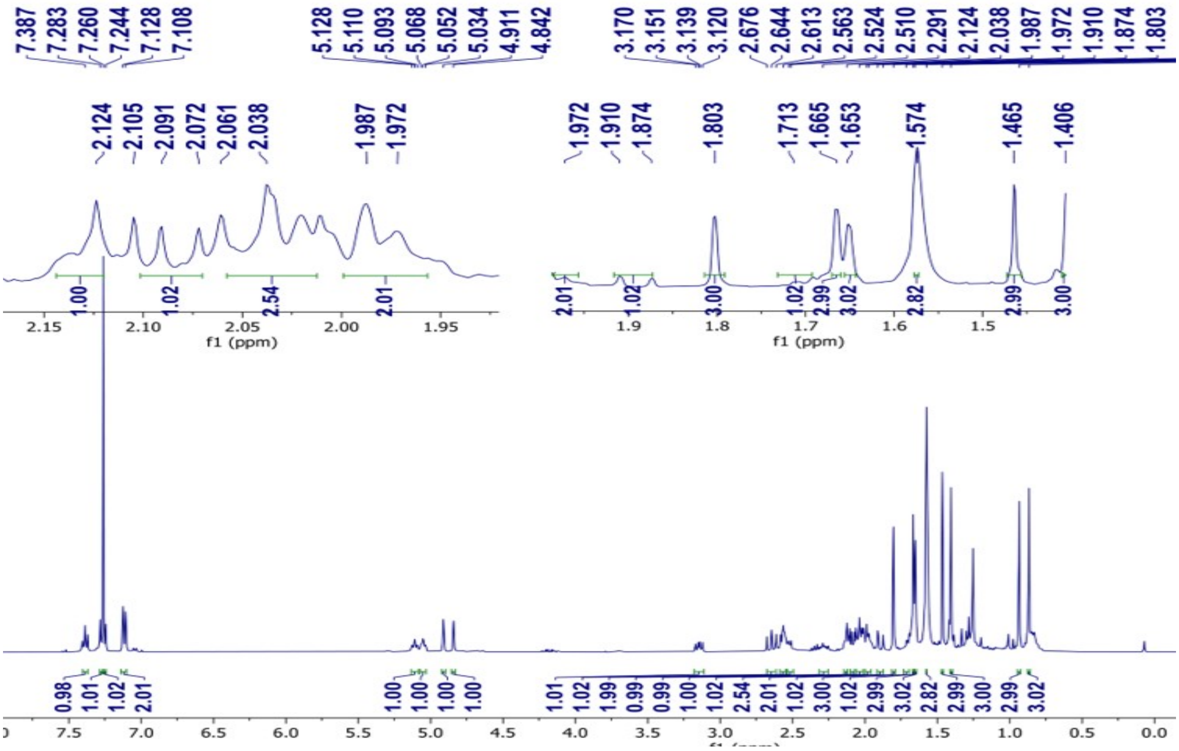

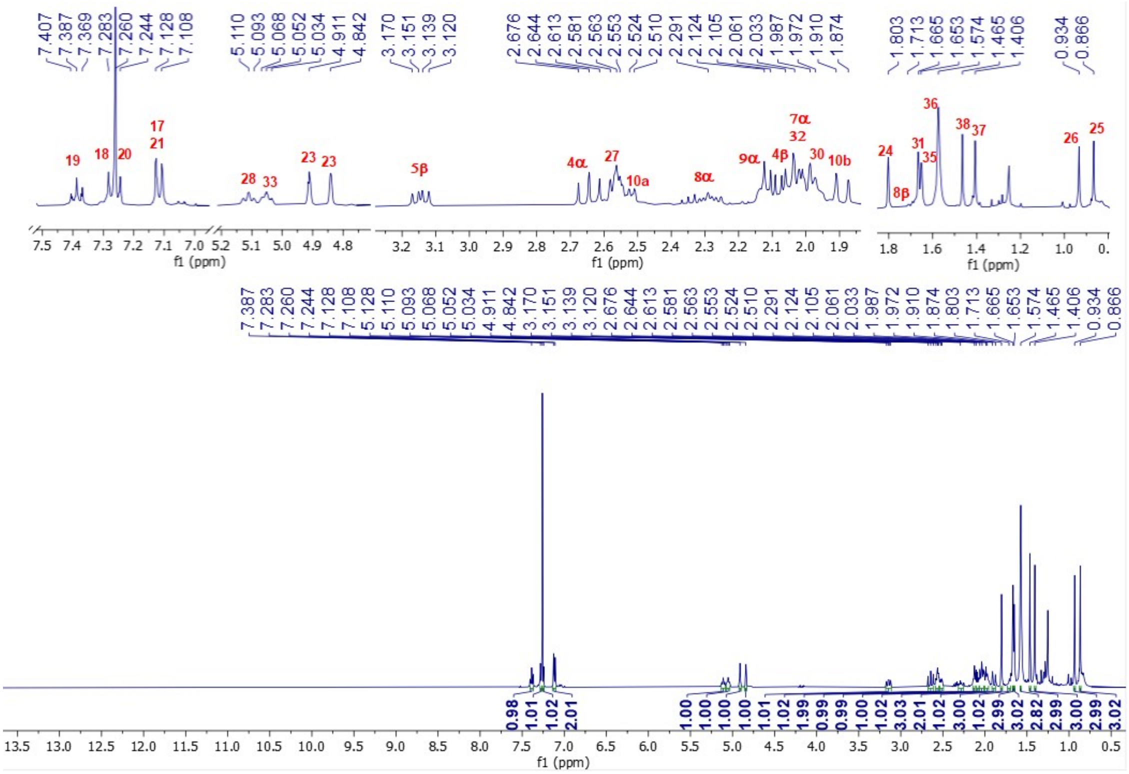

Figure S5B. The ^{1}H NMR spectrum of **5** in $CDCl_3$.

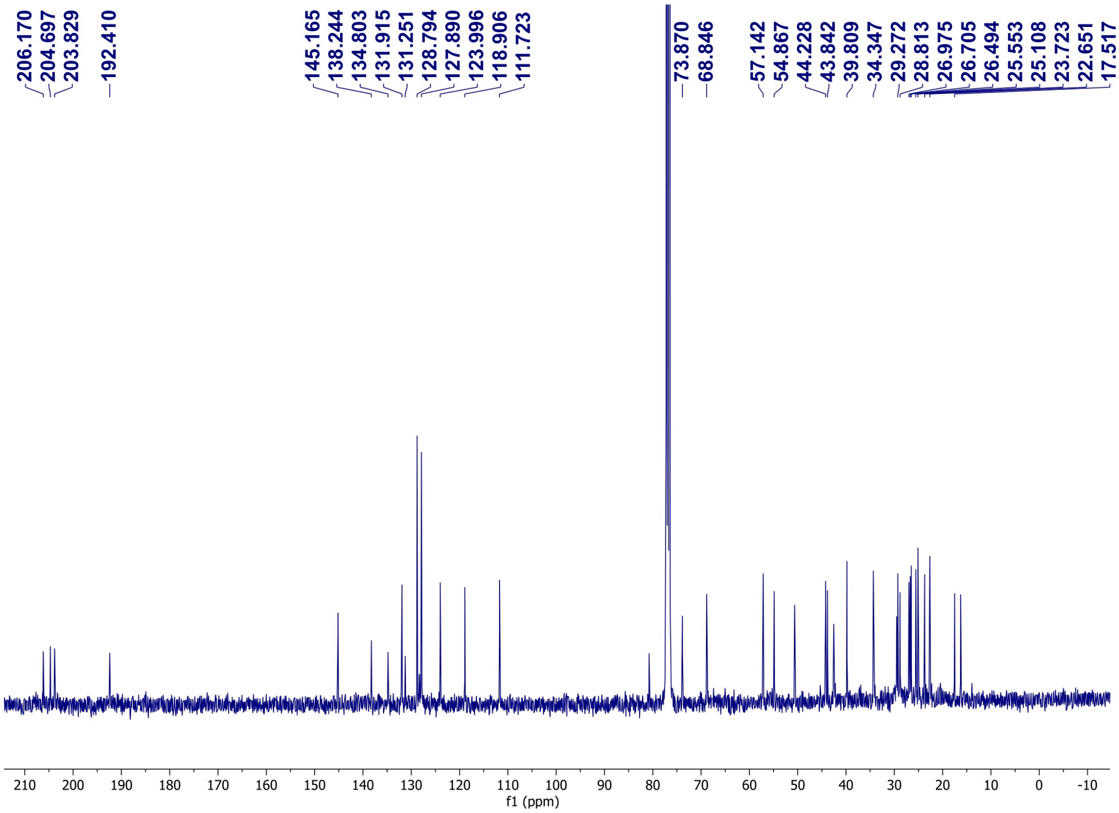

Figure S5C. The ^{13}C NMR spectrum of **5** in $CDCl_3$.

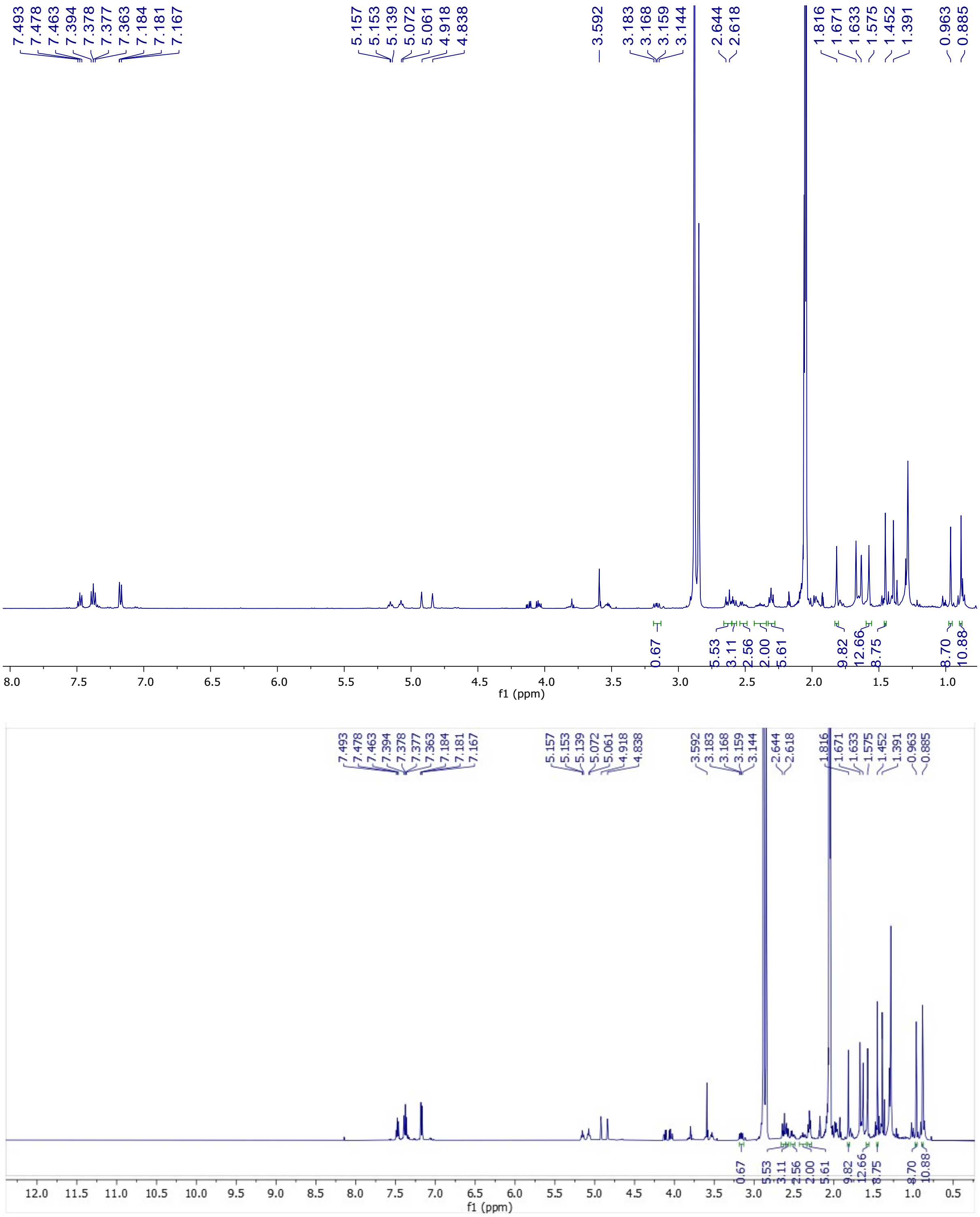

Figure S5D. The ^{1}H NMR spectrum of **5** in acetone-d_6.

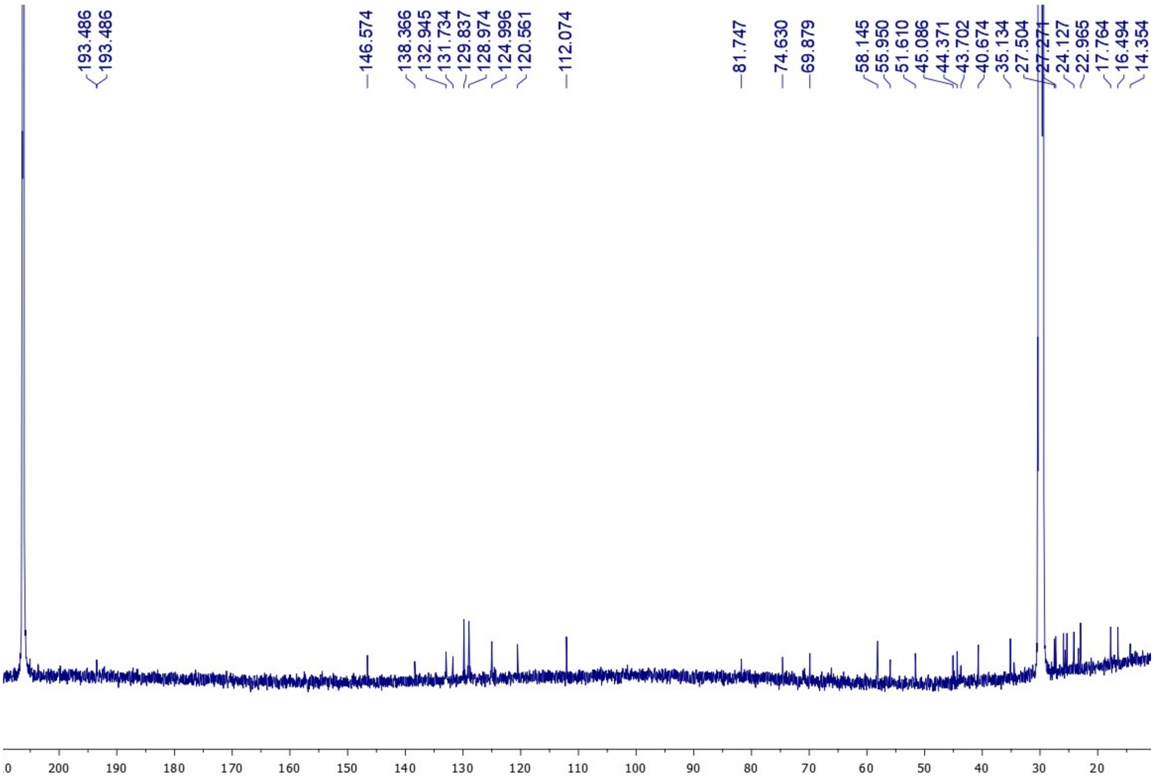

Figure S5E. The ^{13}C NMR spectrum of **5** in acetone-d_6.

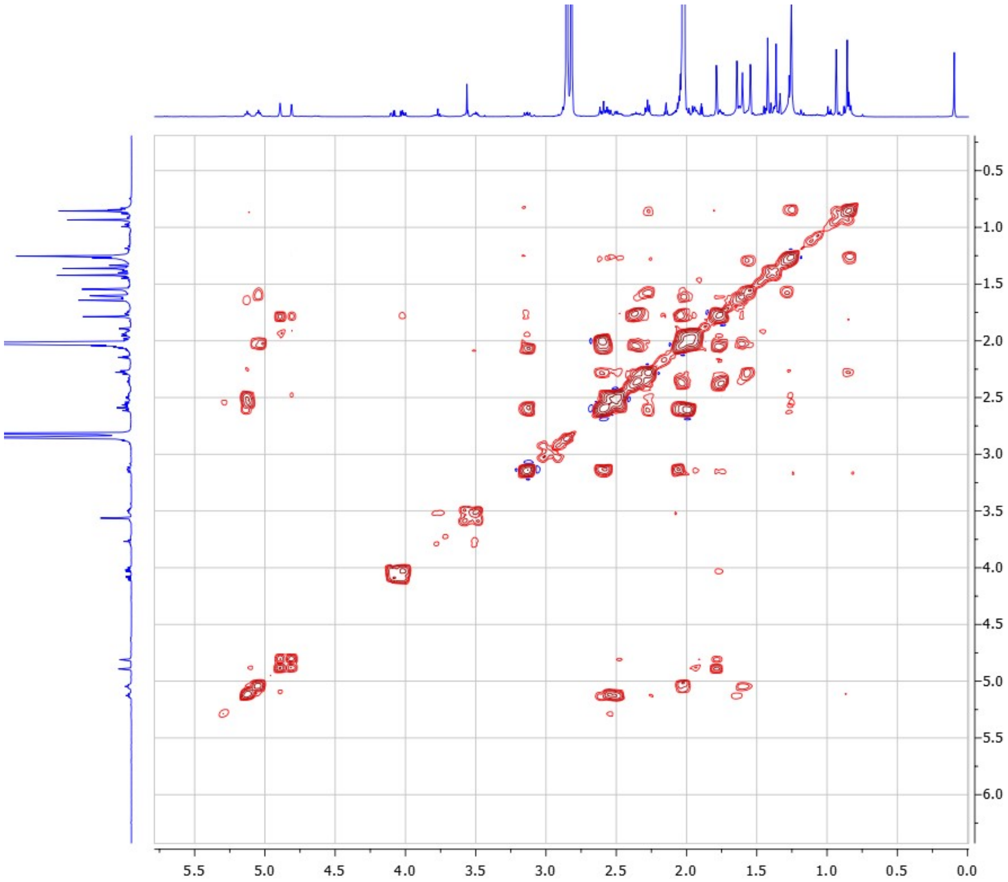

Figure S5F. The COSY spectrum of **5** in acetone-d_6.

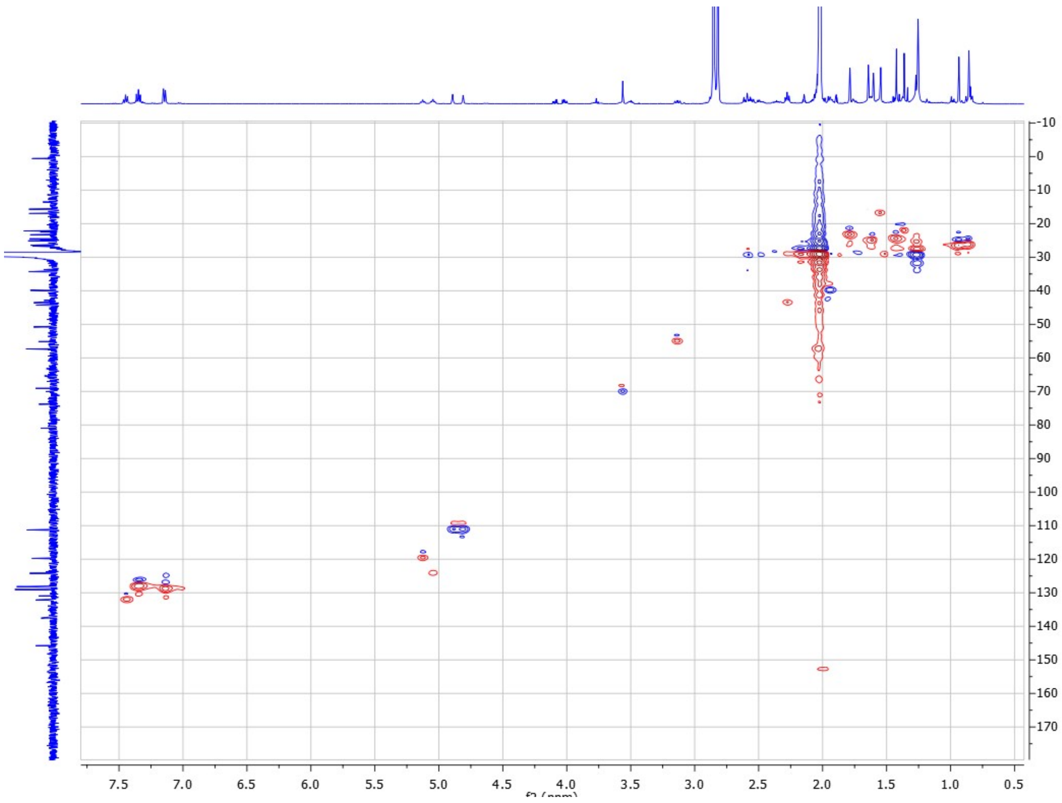

Figure S5G. The HSQC spectrum of **5** in acetone-d_6.

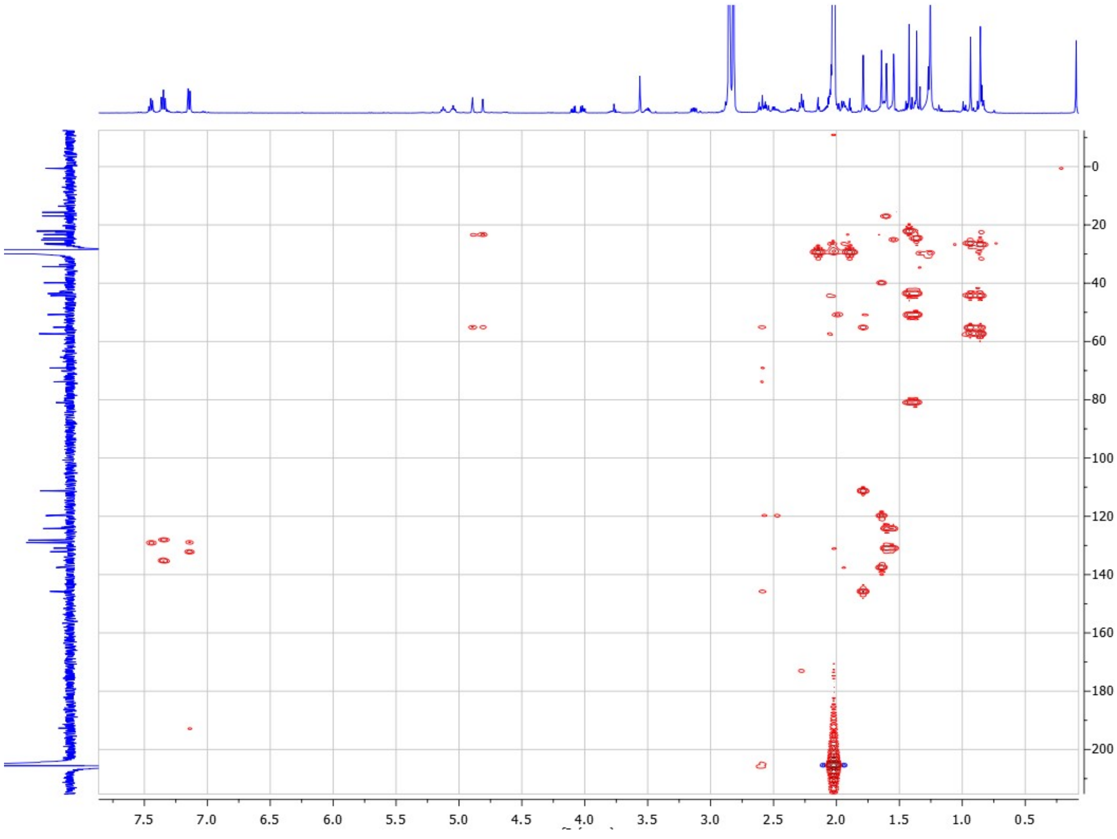

Figure S5H. The HMBC spectrum of **5** in acetone-d_6.

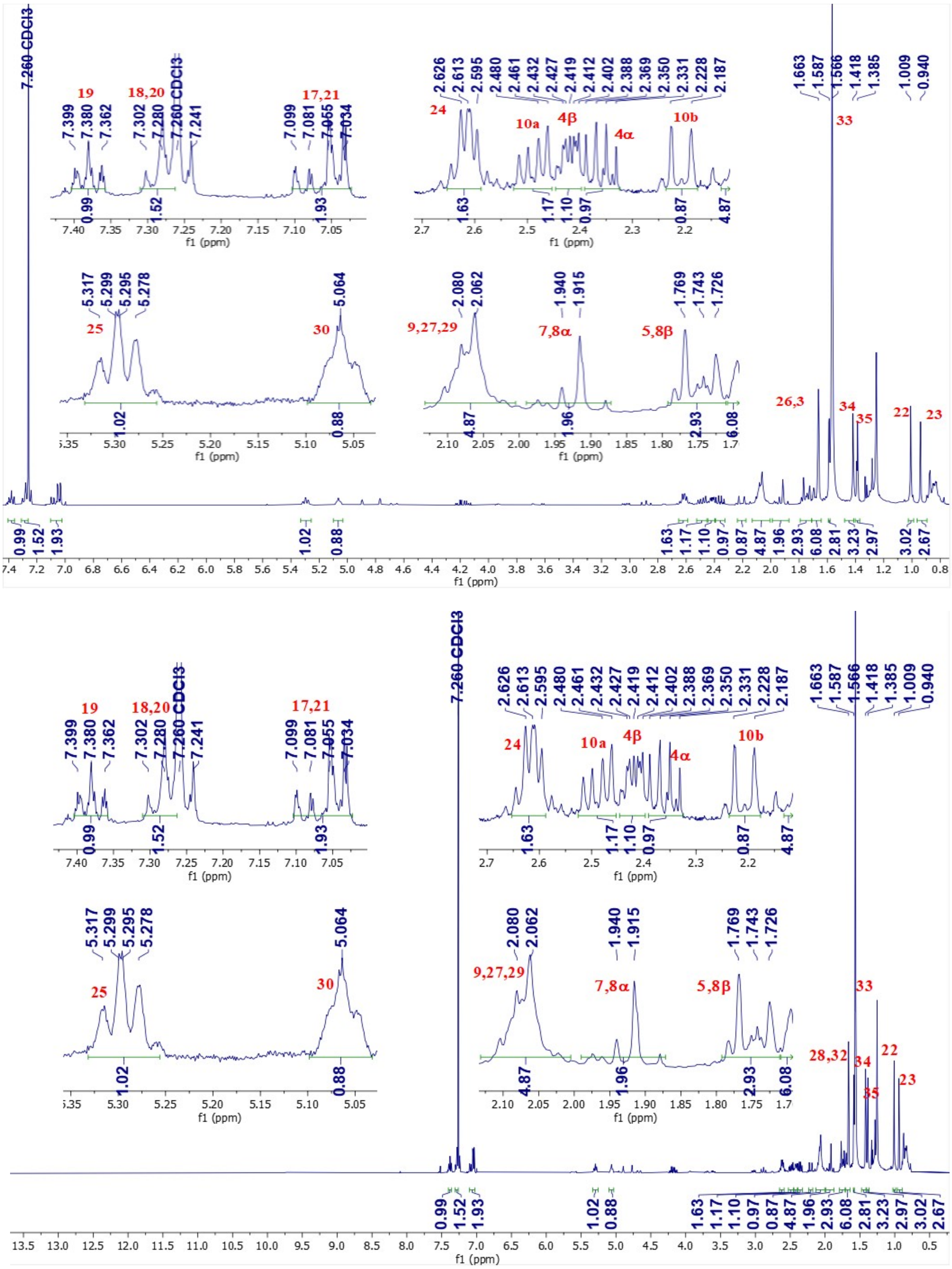

Figure S6A. The ^{1}H NMR spectrum of **6** in $CDCl_3$.

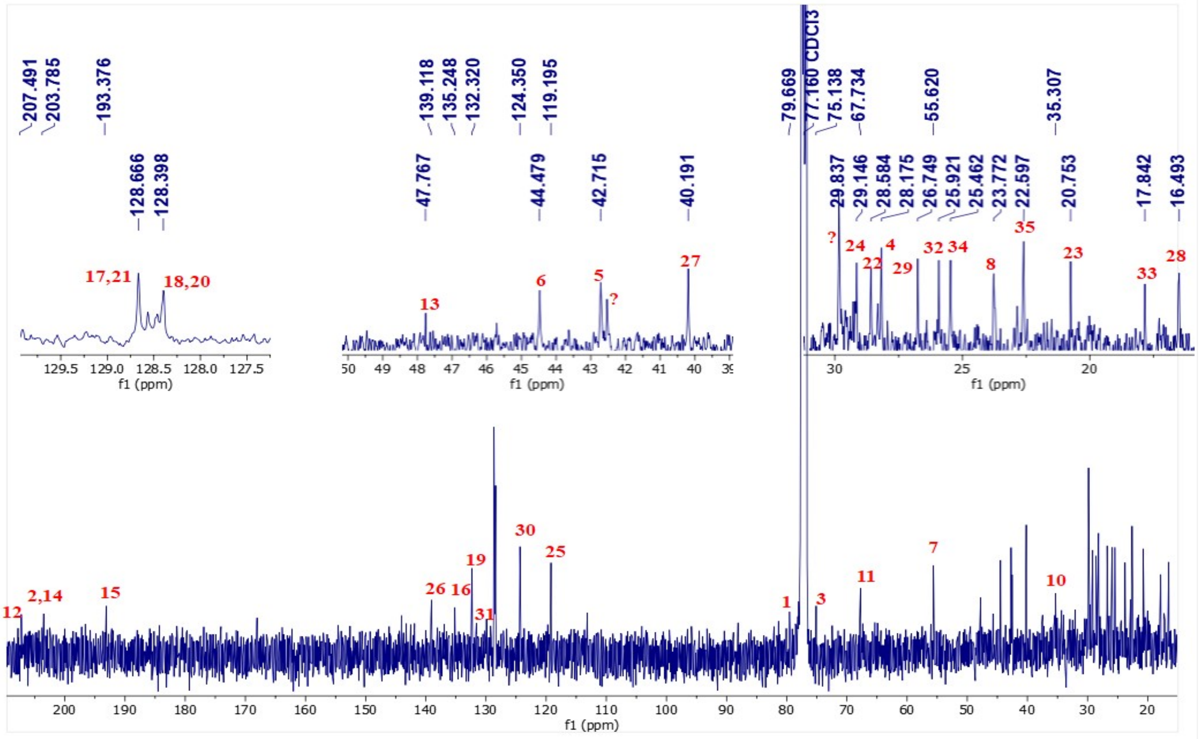

Figure S6B. The ^{13}C NMR spectrum of **6** in $CDCl_3$.

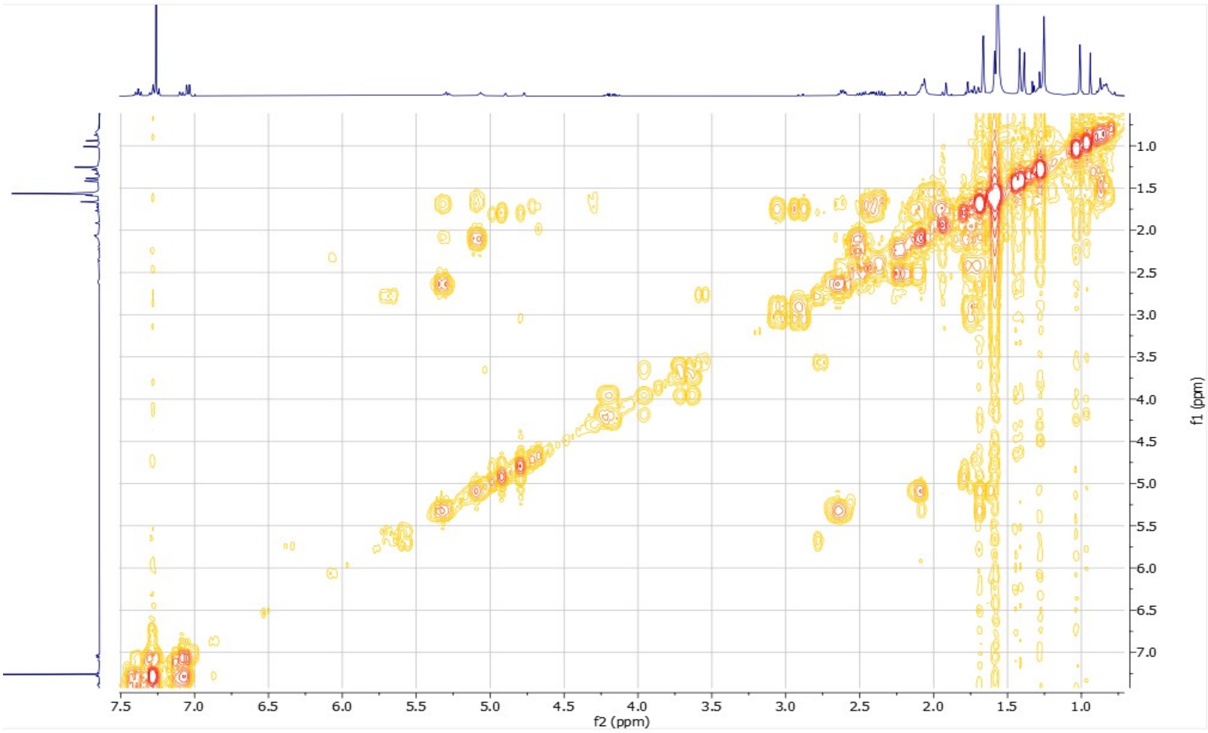

Figure S6C. The COSY spectrum of **6** in $CDCl_3$.

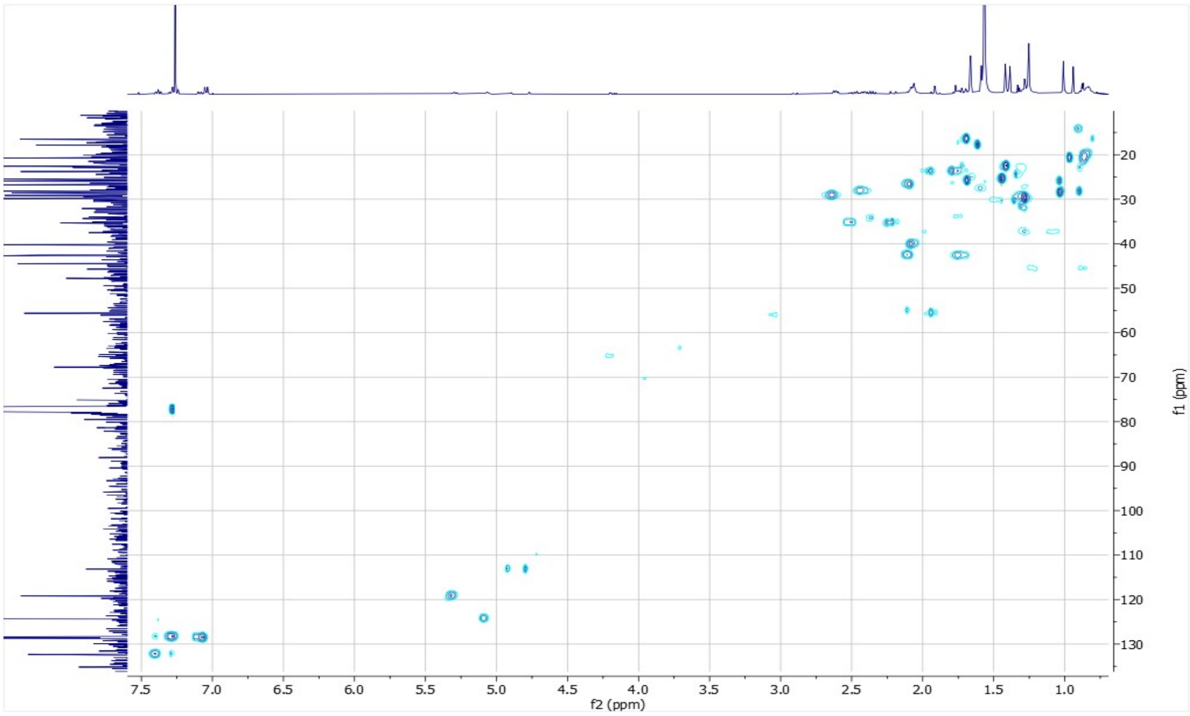

Figure S6D. The HSQC spectrum of **6** in $CDCl_3$.

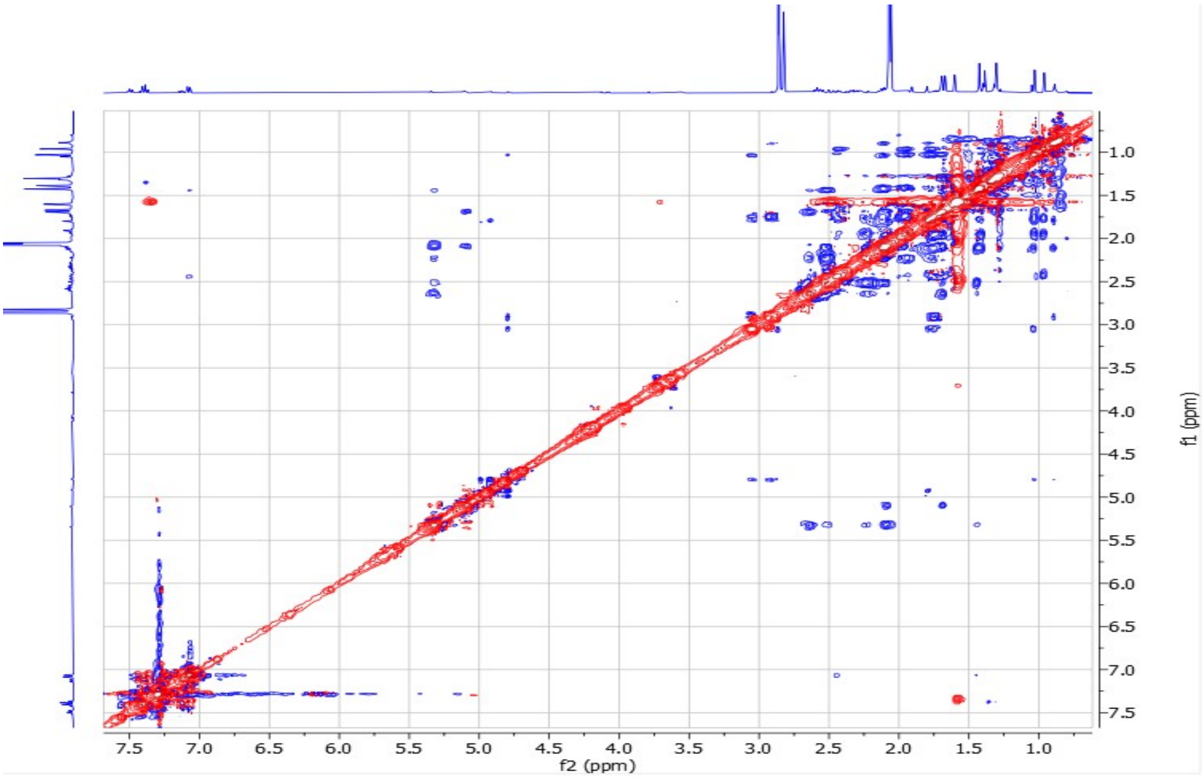

Figure S6E. The NOESY spectrum of **6** in $CDCl_3$.

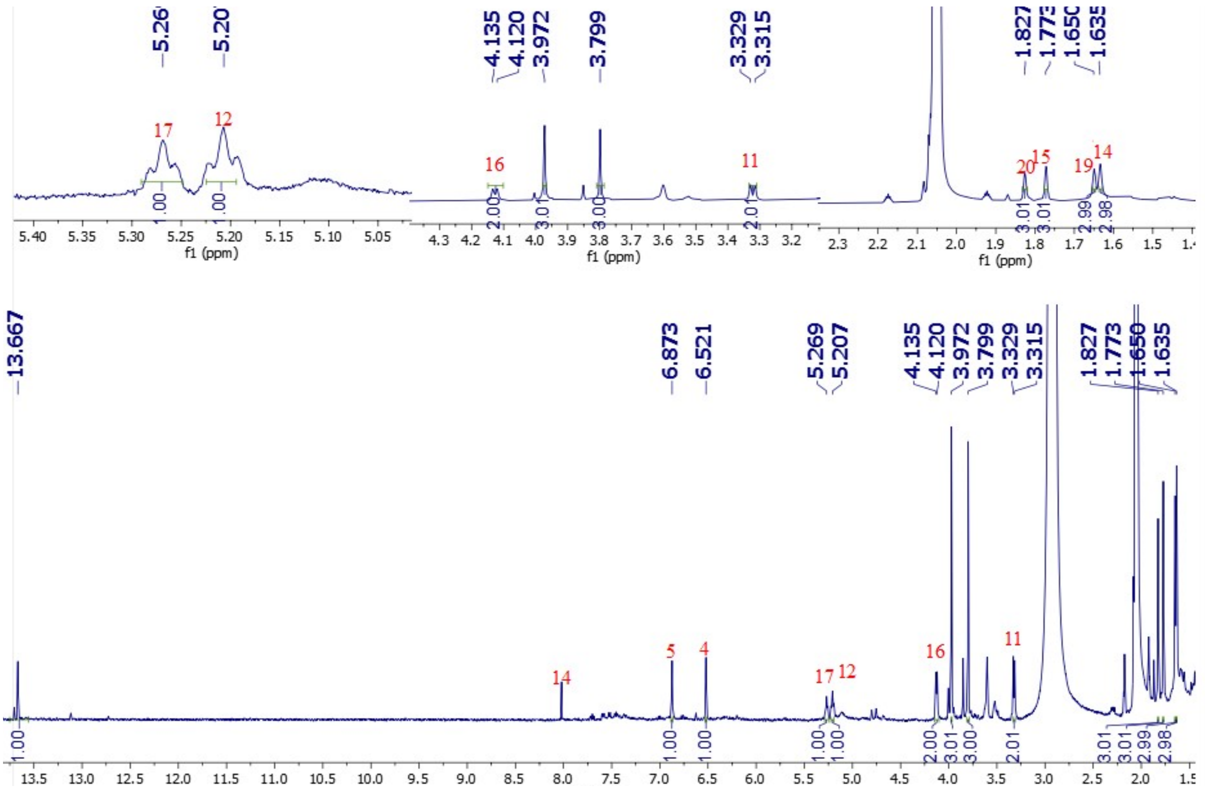

Figure S7A. The ^{1}H NMR spectrum of **7** in acetone-d_6.

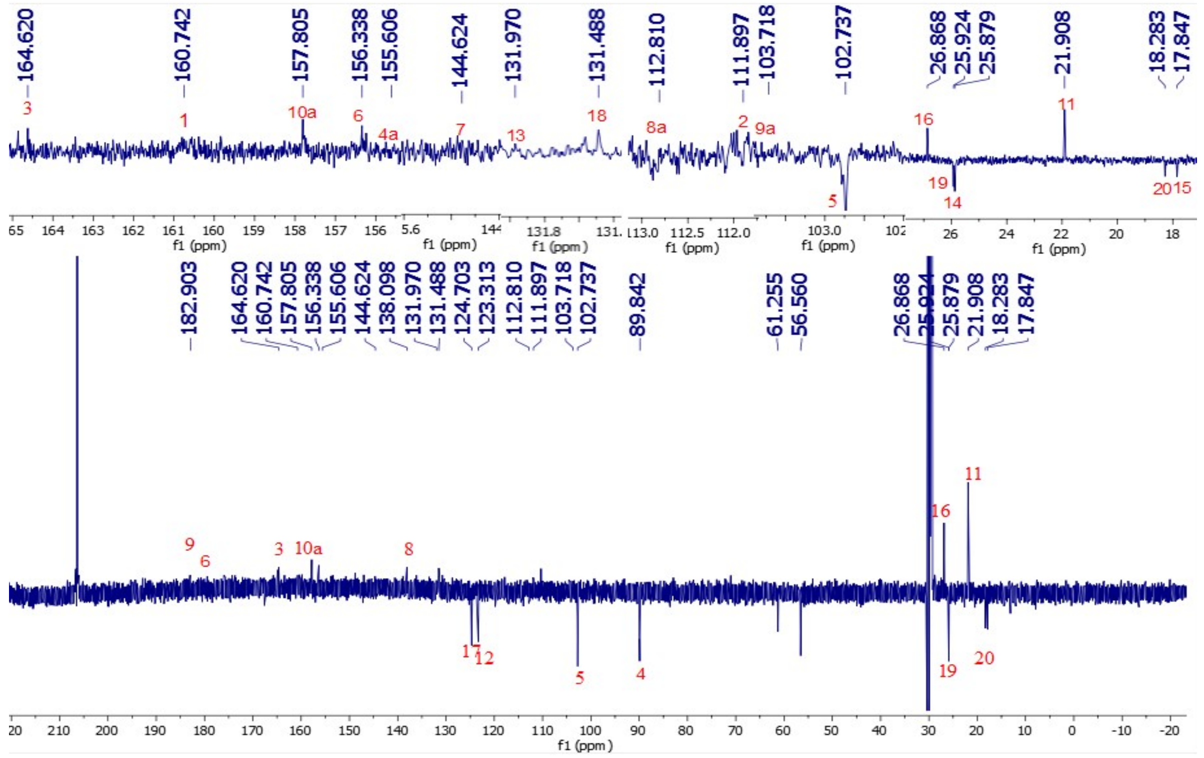

Figure S7B. The ^{13}C NMR spectrum of **7** in acetone-d_6.

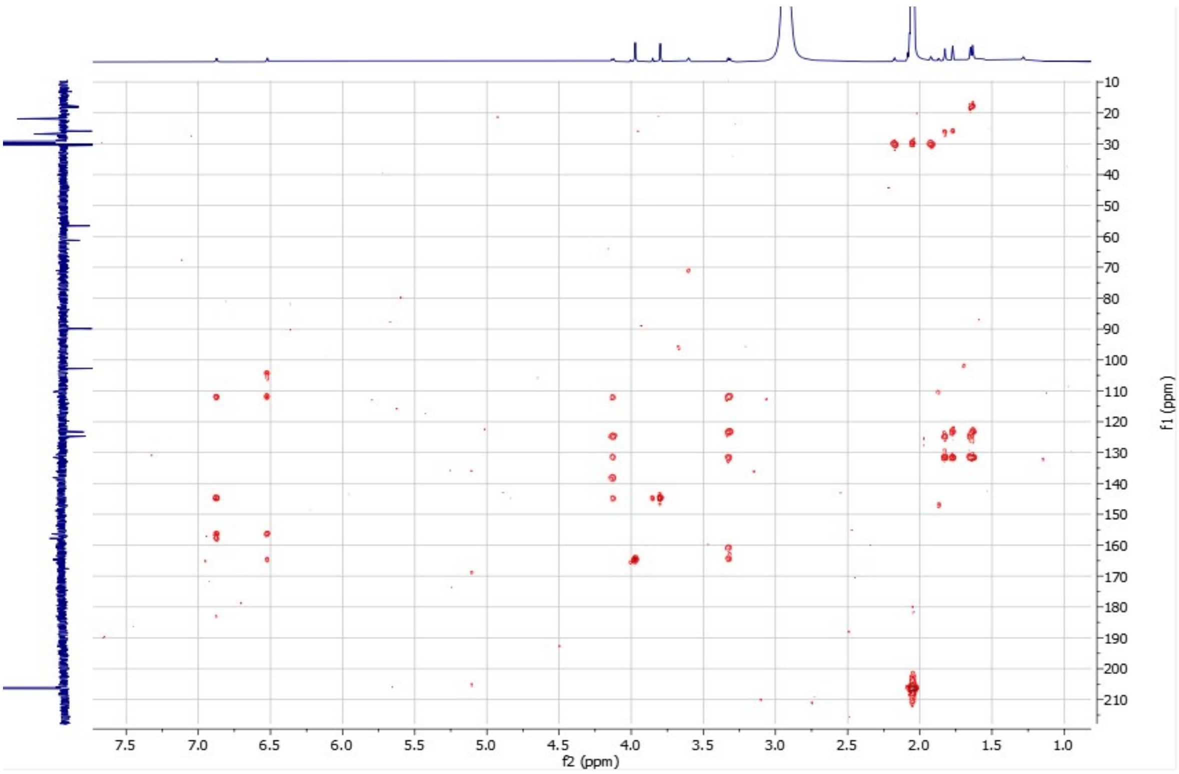

Figure S7C. The HMBC spectrum of **7** in acetone-d_6.

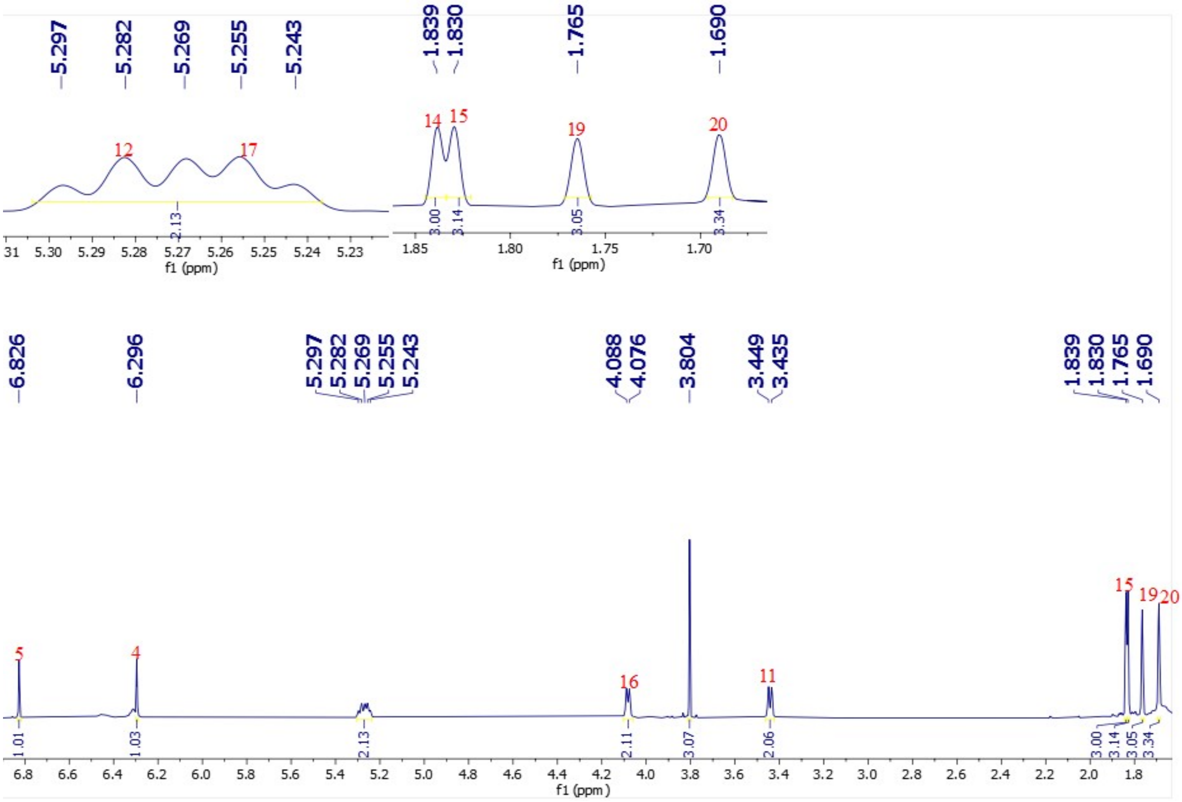

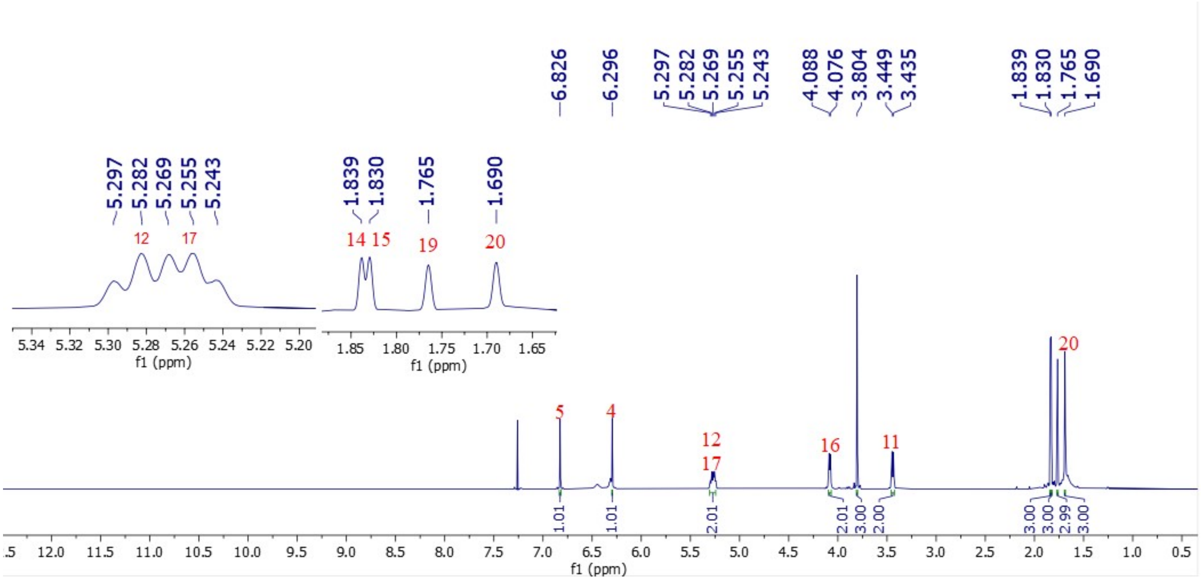

Figure S8A. The ^{1}H NMR spectrum of **8** in $CDCl_3$.

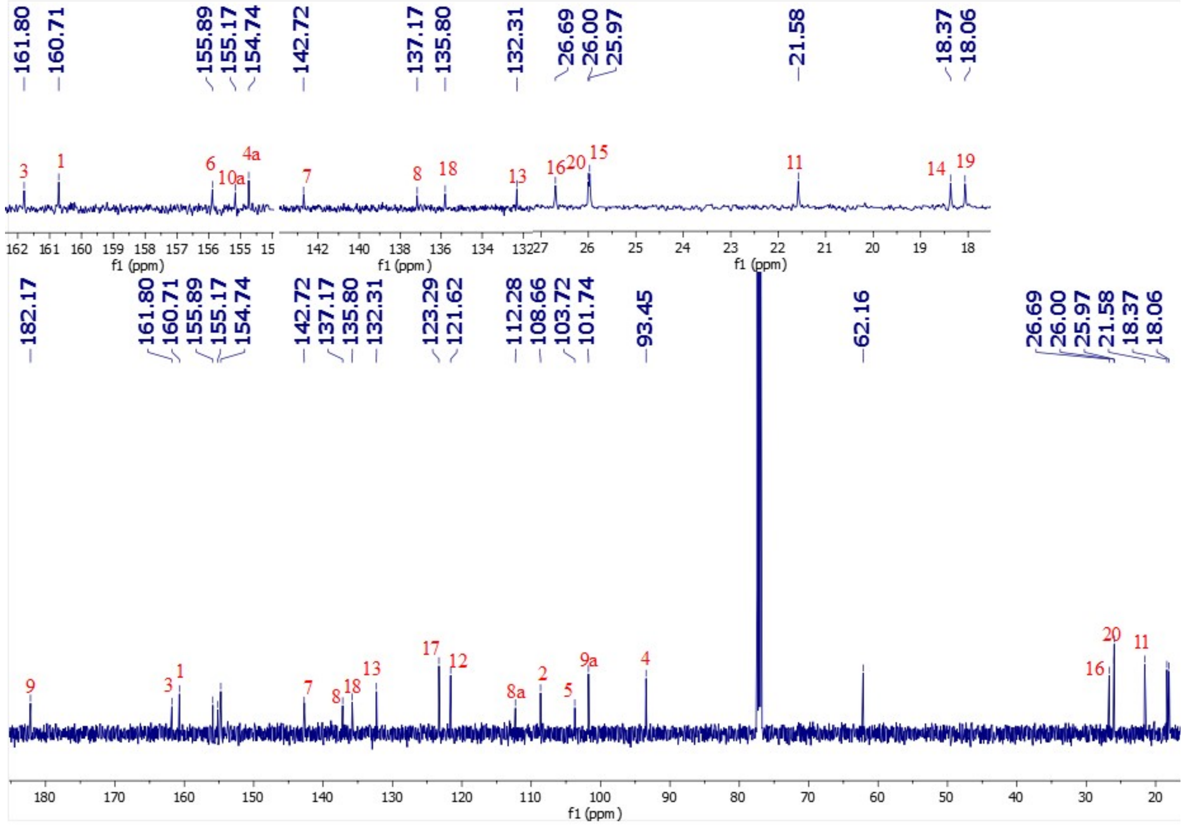

Figure S8B. The ^{13}C NMR spectrum of **8** in $CDCl_3$.

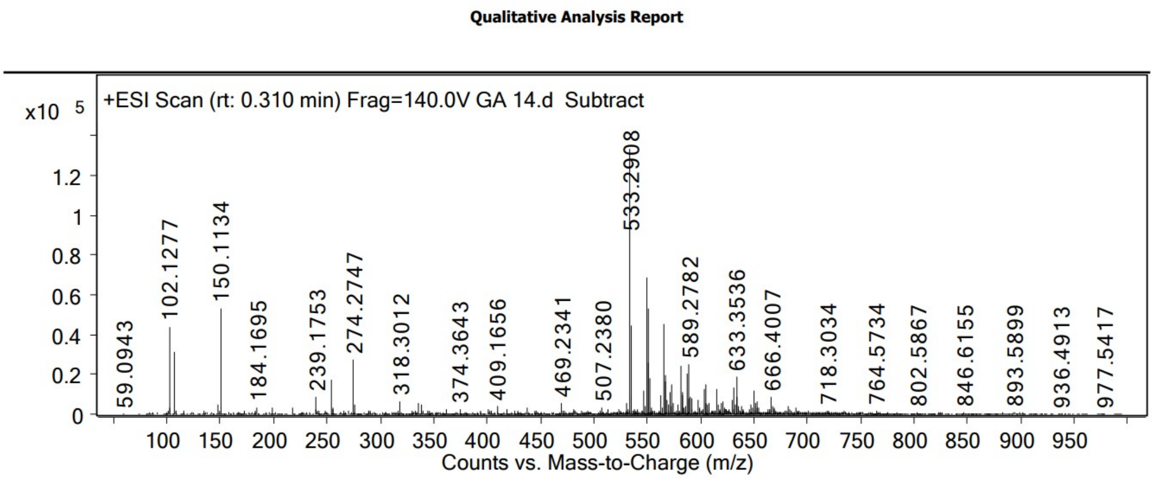

Figure S9A. The HRESIMS spectrum of **9.**

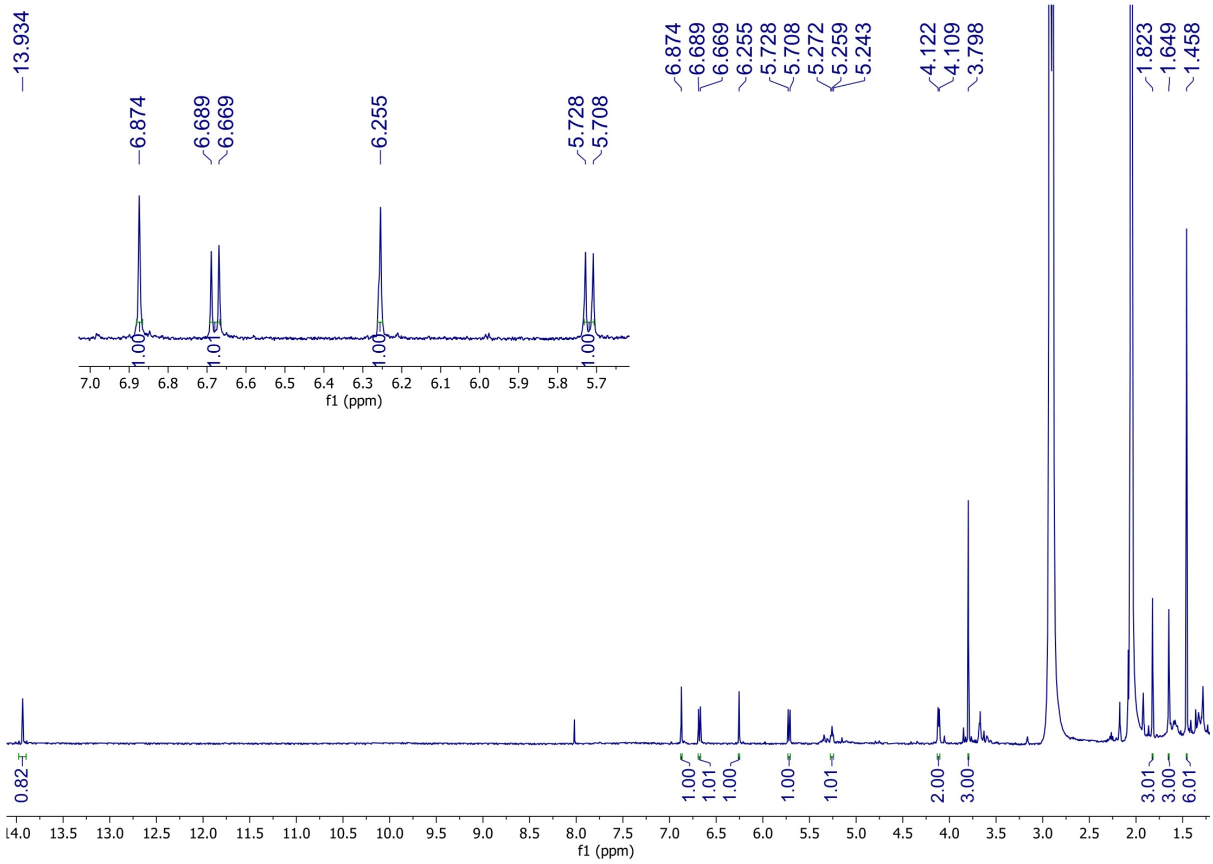

Figure S9B. The ^{1}H NMR spectrum of **9** in acetone-d_6.

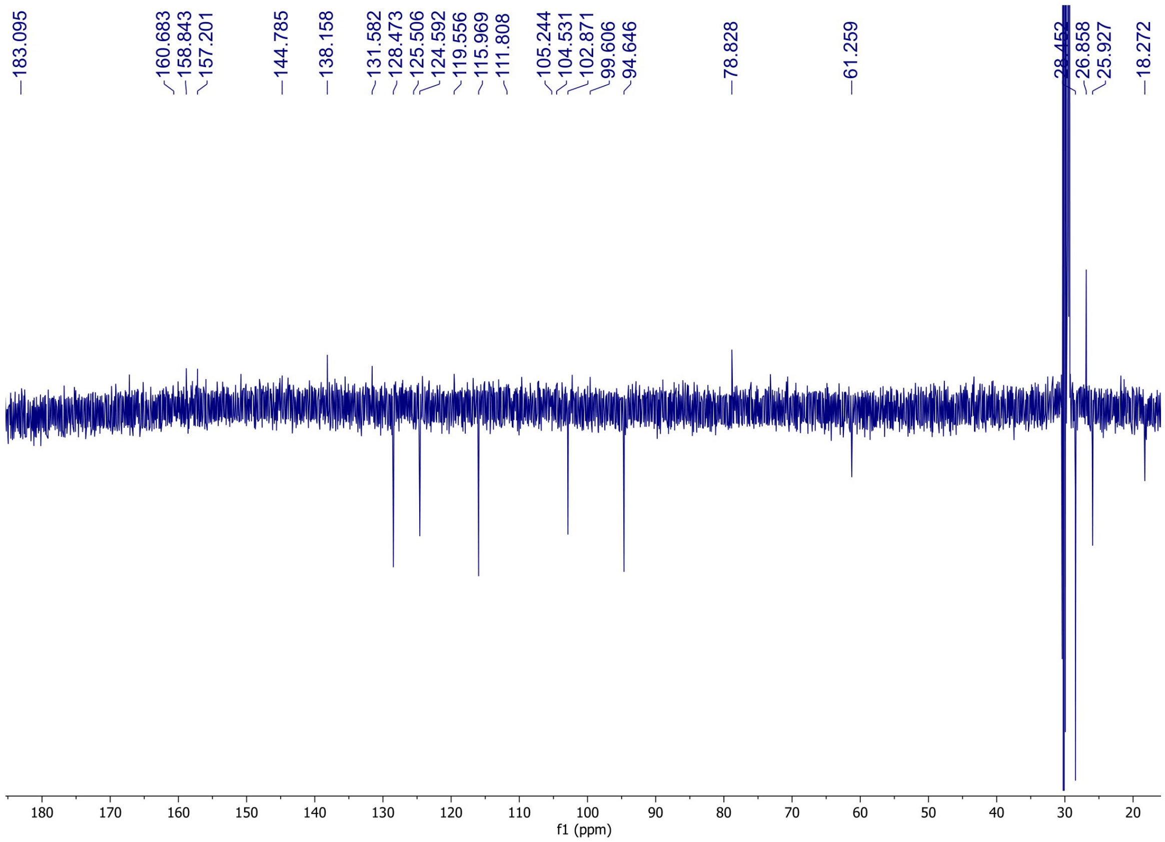

Figure S9C. The JMOD spectrum of **9** in acetone-d_6.

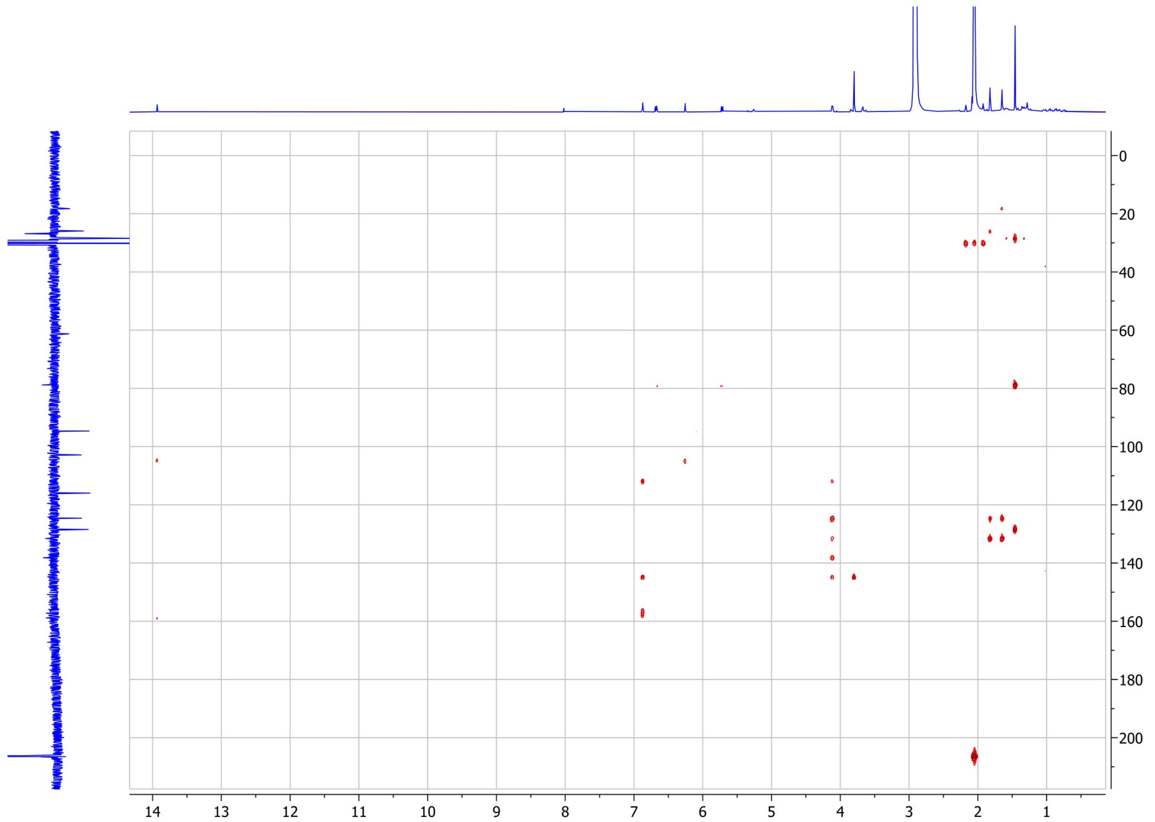

Figure S9D. The HMBC spectrum of **9** in acetone-d_6.

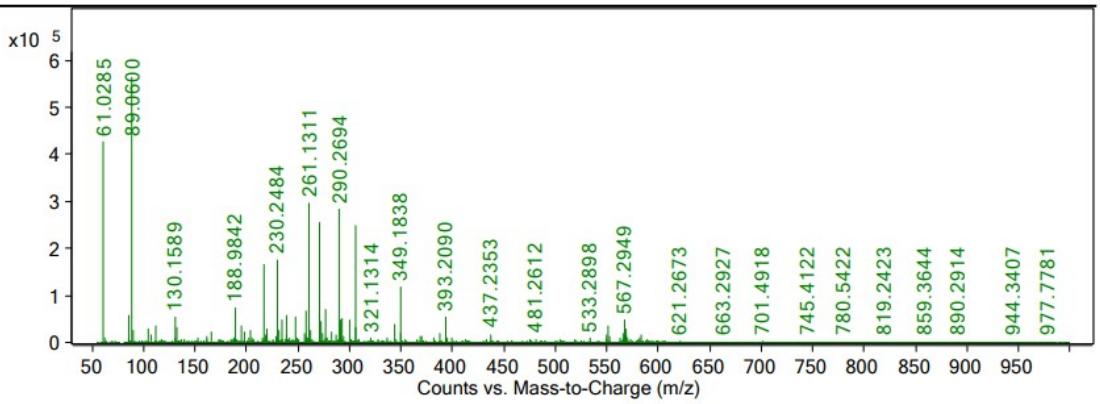

494.3509		355.37
494.564		205.74
494.9647		281.74
495.055		371.22
495.1592		580.25
495.1965		994.42
495.2352	1	1494.97
495.2965		602.13
495.4555	2	303.1
495.4863		332.25
495.5369		216.25
495.7124		232.95
495.8749		202.86
495.9645	2	236.01
496.1195		200.75
496.1633		541.26
496.2064		784.31
496.2439	1	759.11
496.307		429.56
496.3364	2	297.27
496.4195		242.72
496.4933		340.88
496.5826		232.44

Figure S10A. The HRESIMS spectrum of **10.**

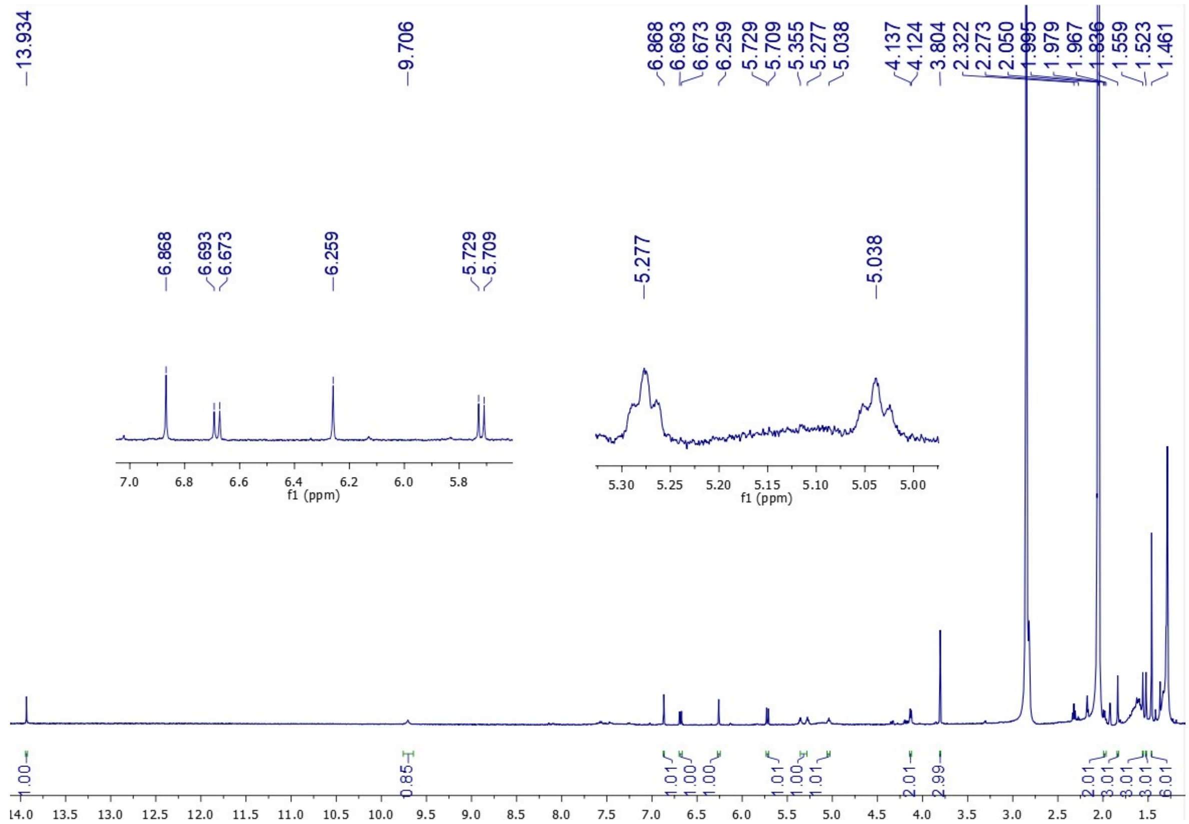

Figure S10B. The ^{1}H NMR spectrum of **10** in acetone-d_6.

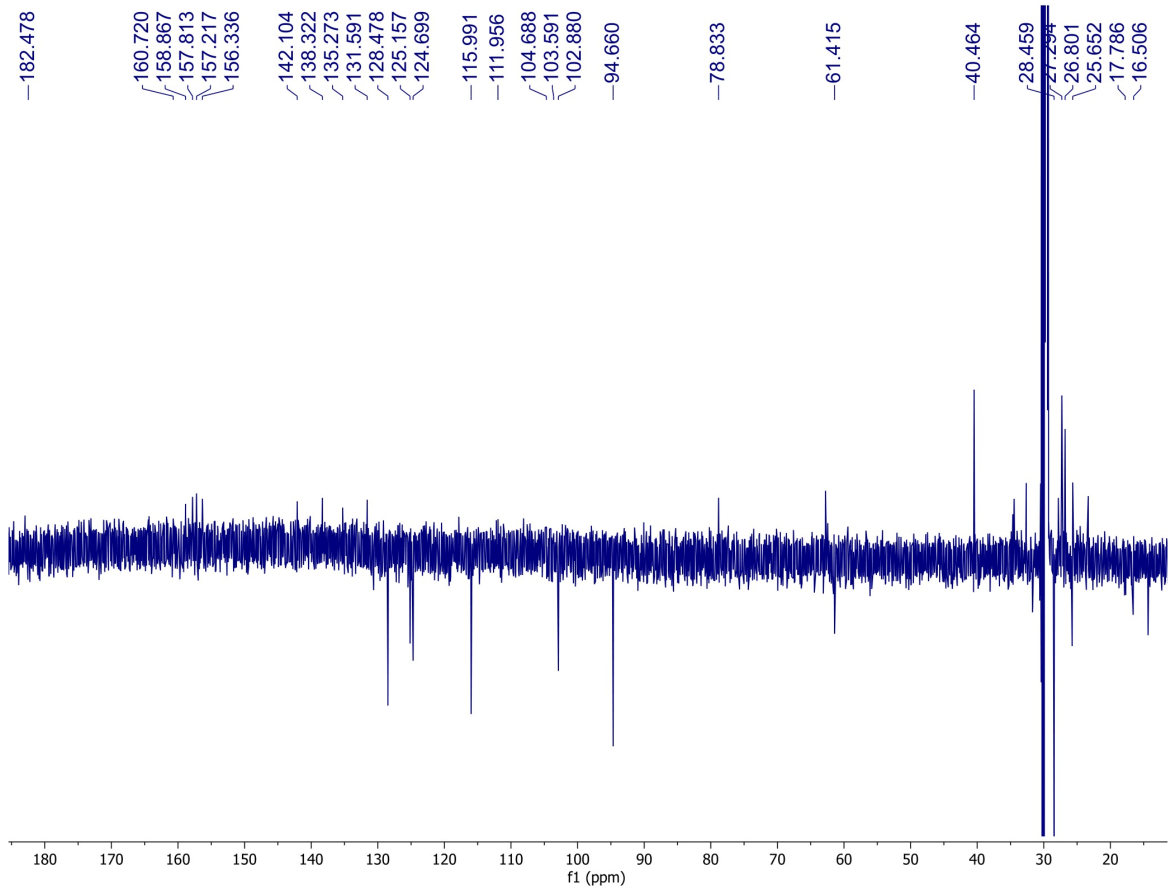

Figure S10C. The JMOD spectrum of **10** in acetone-d_6.

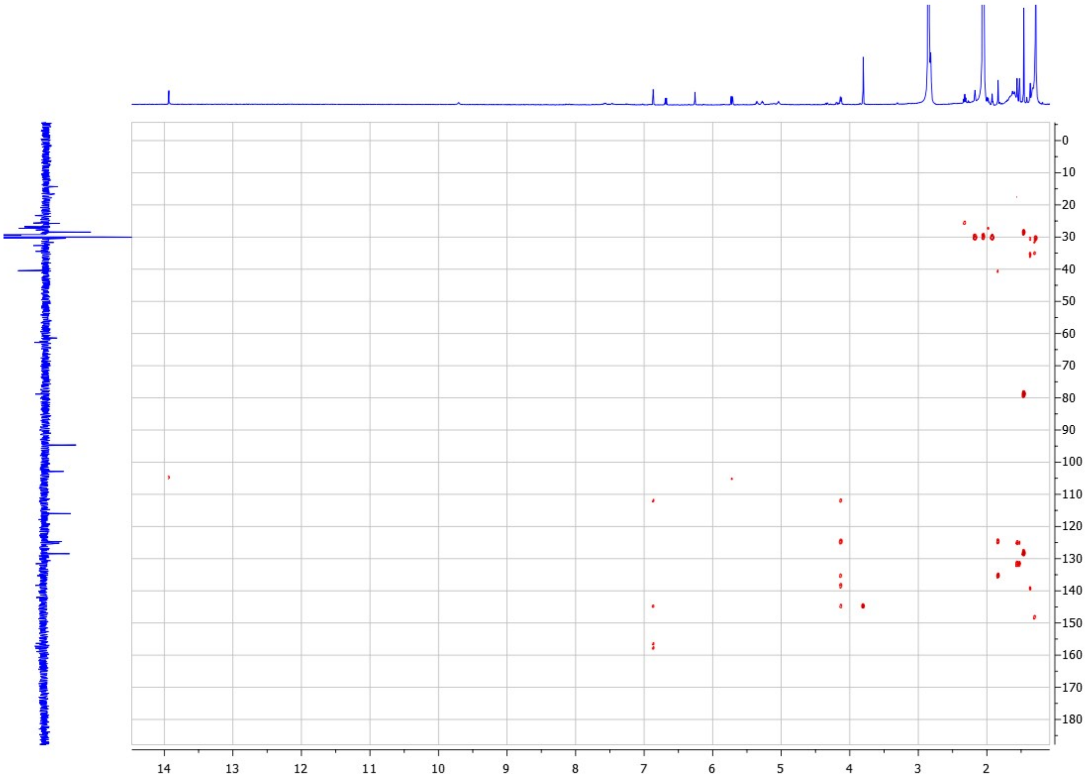

Figure S10D. The HMBC spectrum of **10** in acetone-d_6.

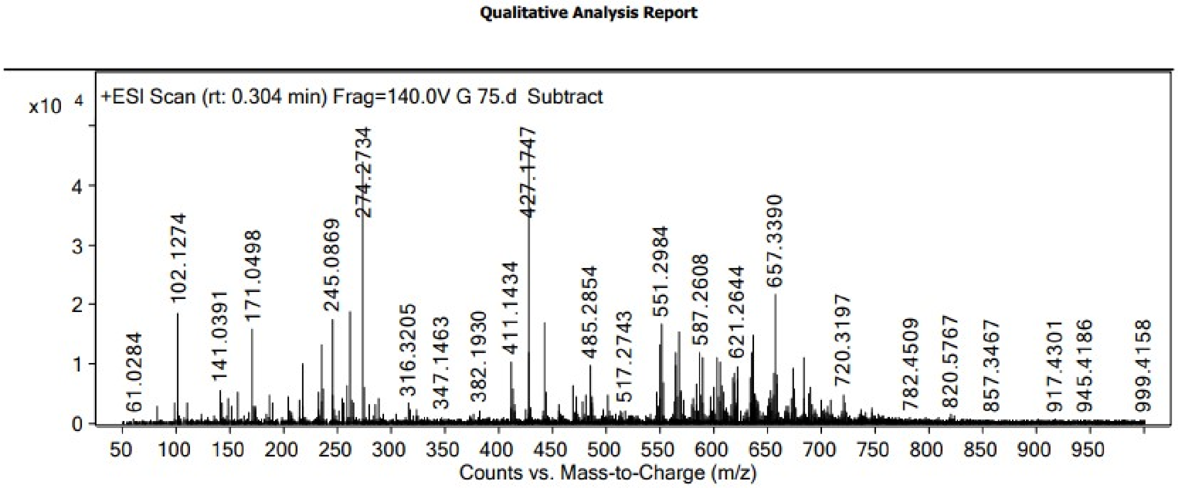

Figure S11A. The HRESIMS spectrum of **11.**

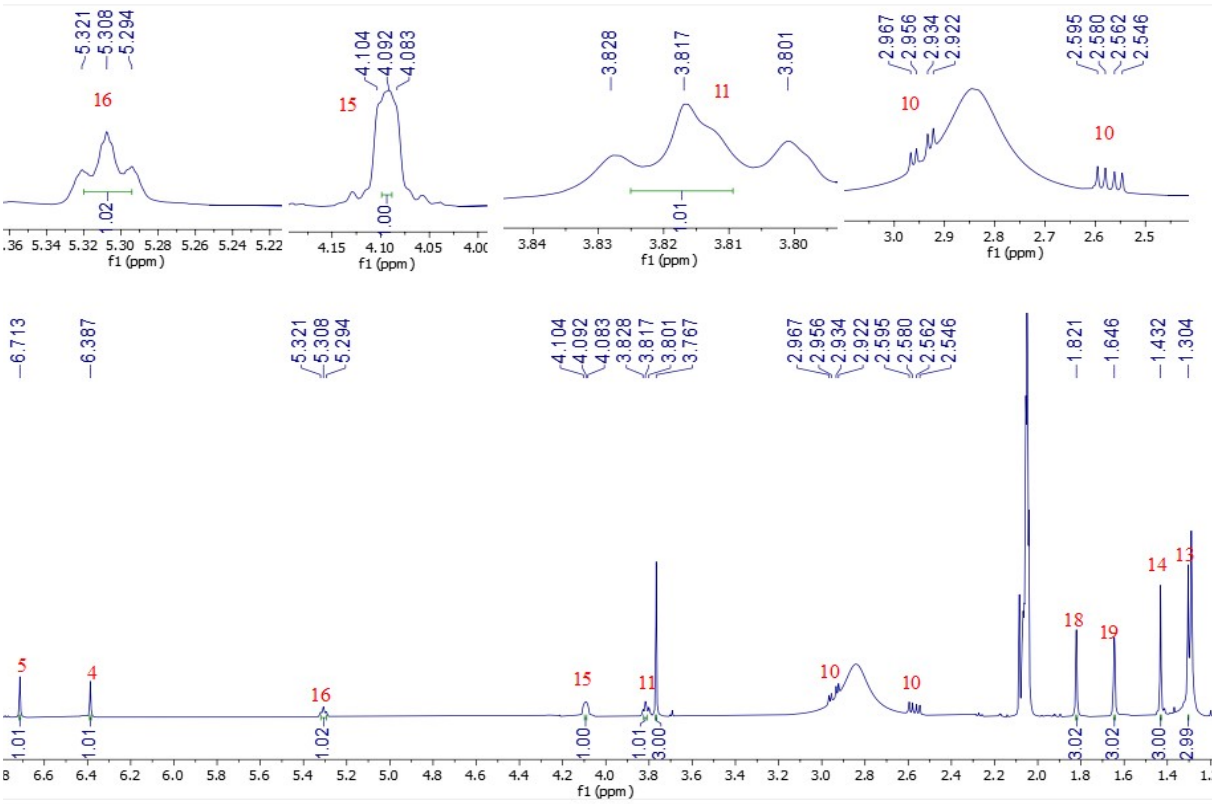

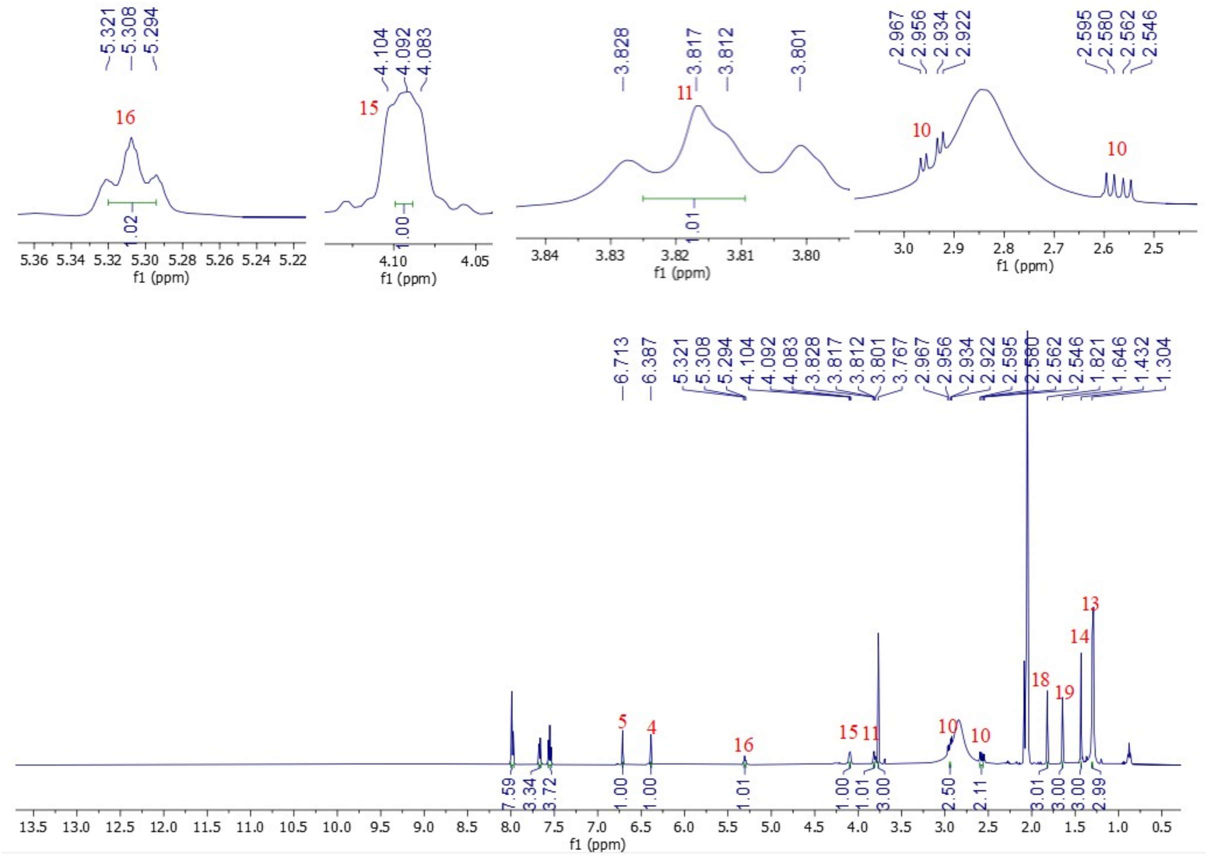

Figure S11B. The ^{1}H NMR spectrum of **11** in acetone-d_6.

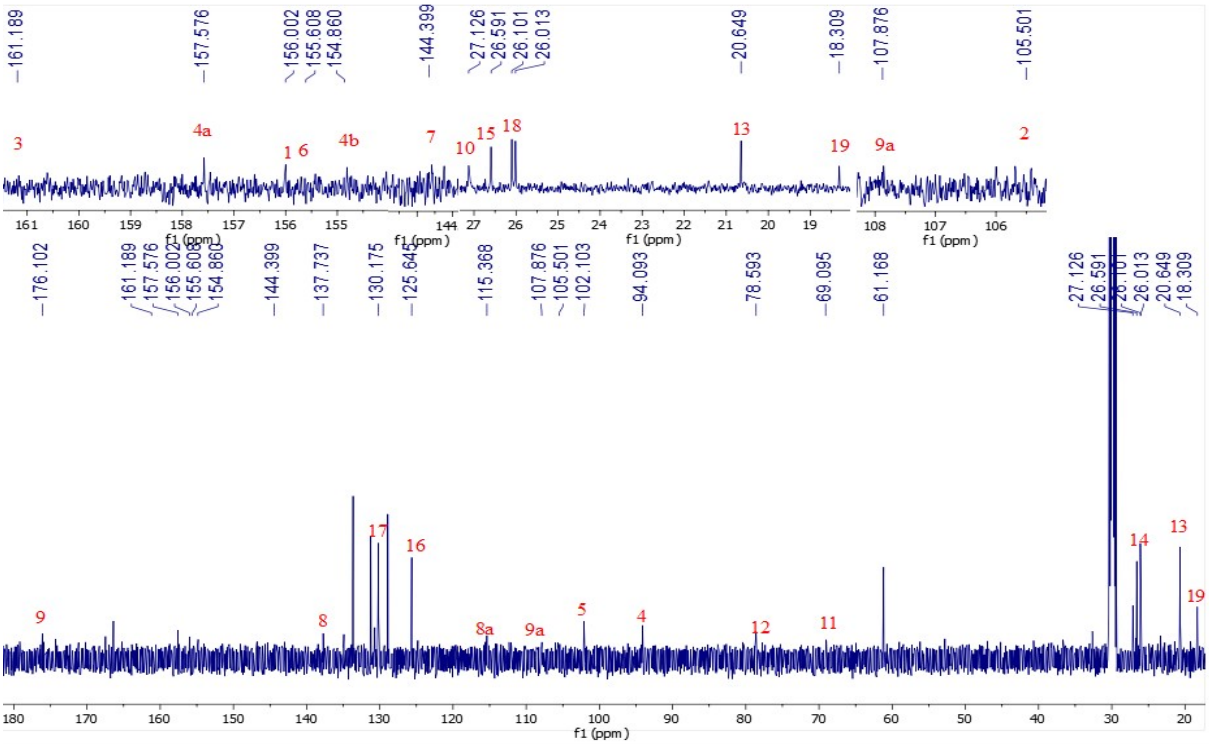

Figure S11C. The ^{13}C NMR spectrum of **11** in acetone-d_6.

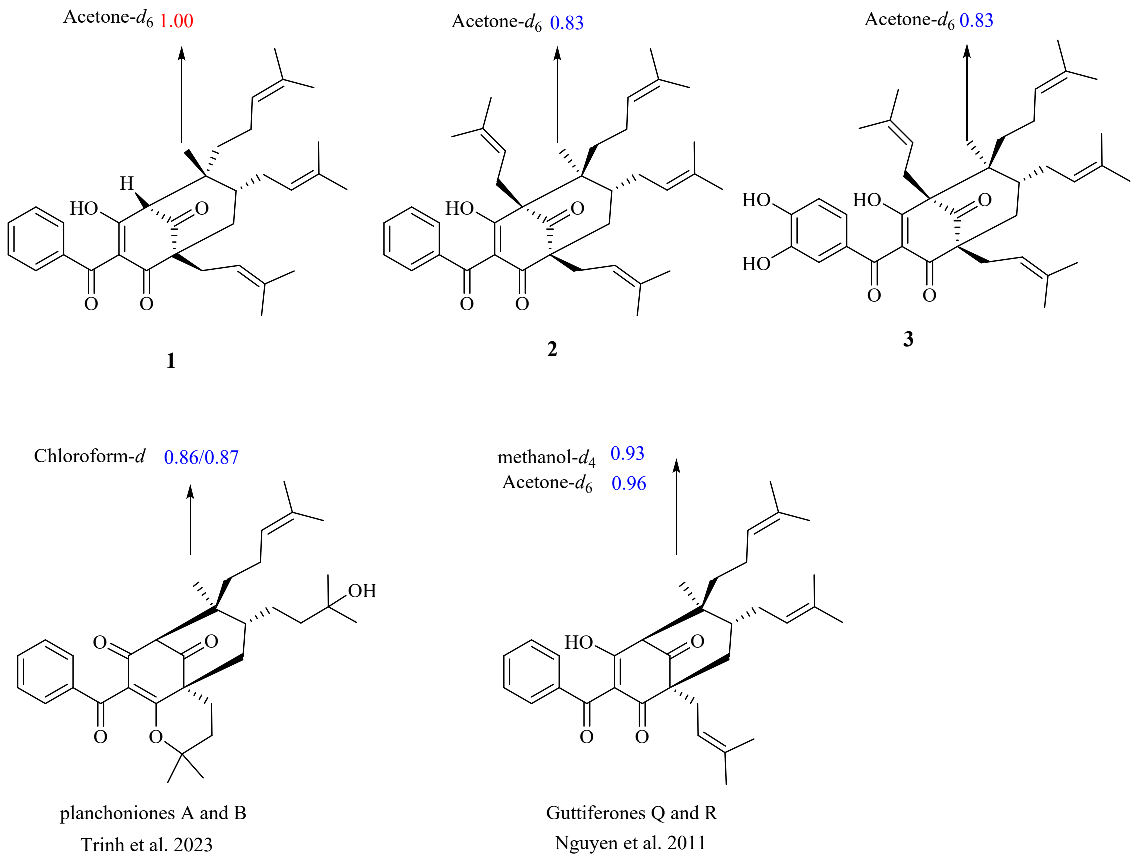

Figure S12. The chemical shifts of the characteristic methyl group of benzoylphloroglucinols **1-3**, planchoniones A and B, guttiferones Q and R.

Refs:

X.-T. Yan, Z. An, Y. Huangfu, et al. Polycyclic polyprenylated acylphloroglucinol and phenolic metabolites from the aerial parts of Hypericum elatoides and their neuroprotective and anti-neuroinflammatory activities. *Phytochemistry* 2019, 159, 65–74.

H.D. Nguyen, B.T.D. Trinh, L.-H.D. Nguyen. Guttiferones Q-S, cytotoxic polyisoprenylated benzophenones from the pericarp of Garcinia cochinchinensis. *Phytochemistry Letters* 2011, 4 (2), 129–133.

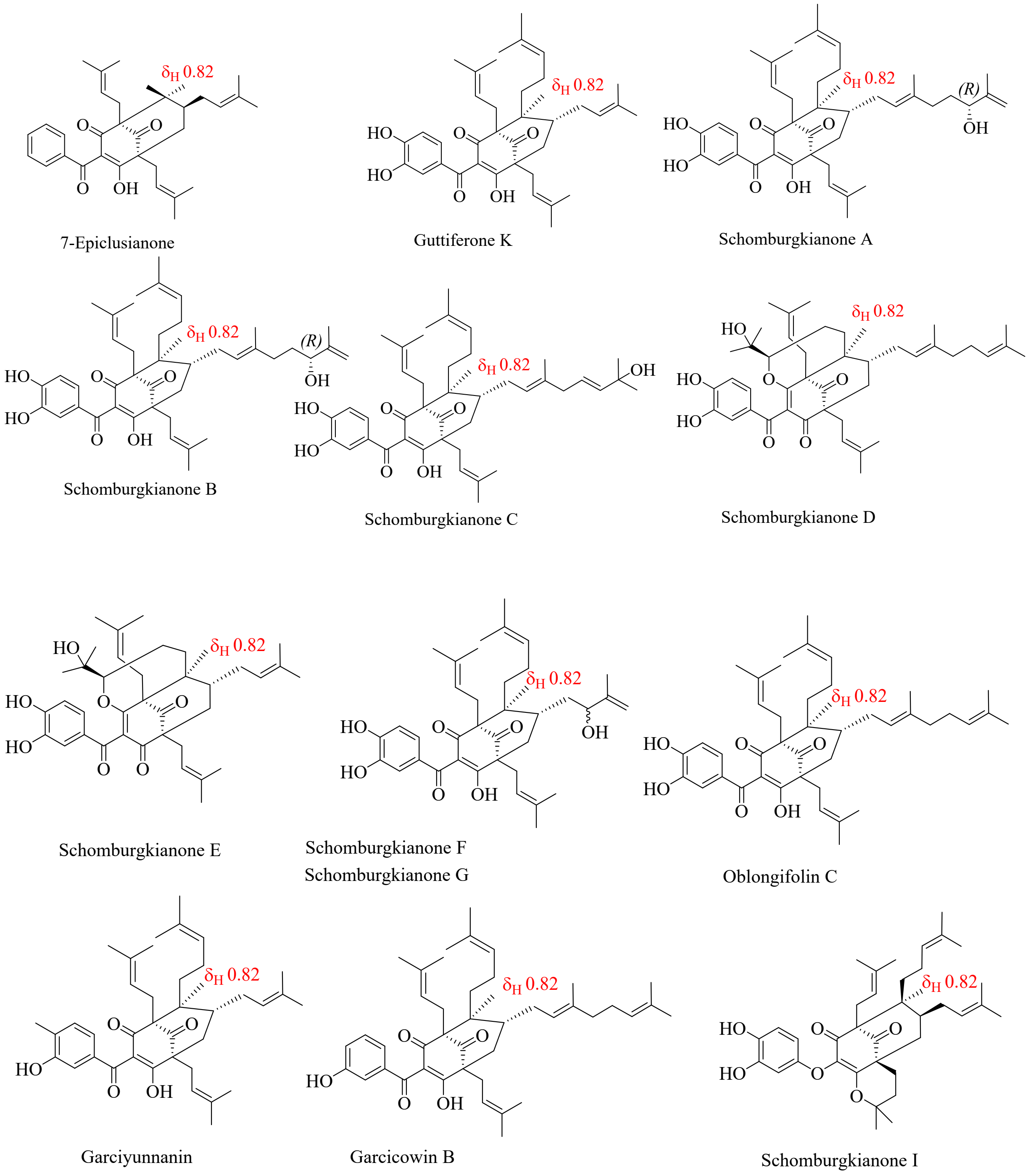

Figure S13. The chemical shifts of the characteristic methyl group of benzoylphloroglucinols from Vietnamese *G. schomburgkiana* fruits.

D.H. Le, K. Nishimura, Y. Takenaka, Y. Mizushina, T. Tanahashi. Polyprenylated Benzoylphloroglucinols with DNA Polymerase Inhibitory Activity from the Fruits of *Garcinia schomburgkiana*. *J. Nat. Prod.* 2016, 79 (7), 1798–1807.

H.T. Nguyen, T.-T. Nguyen, T.-H. Duong, et al. α-Glucosidase Inhibitory and Antimicrobial Benzoylphloroglucinols from Garcinia schomburgakiana Fruits: In Vitro and In Silico Studies. *Molecules* 2022, 27 (8), 2574.

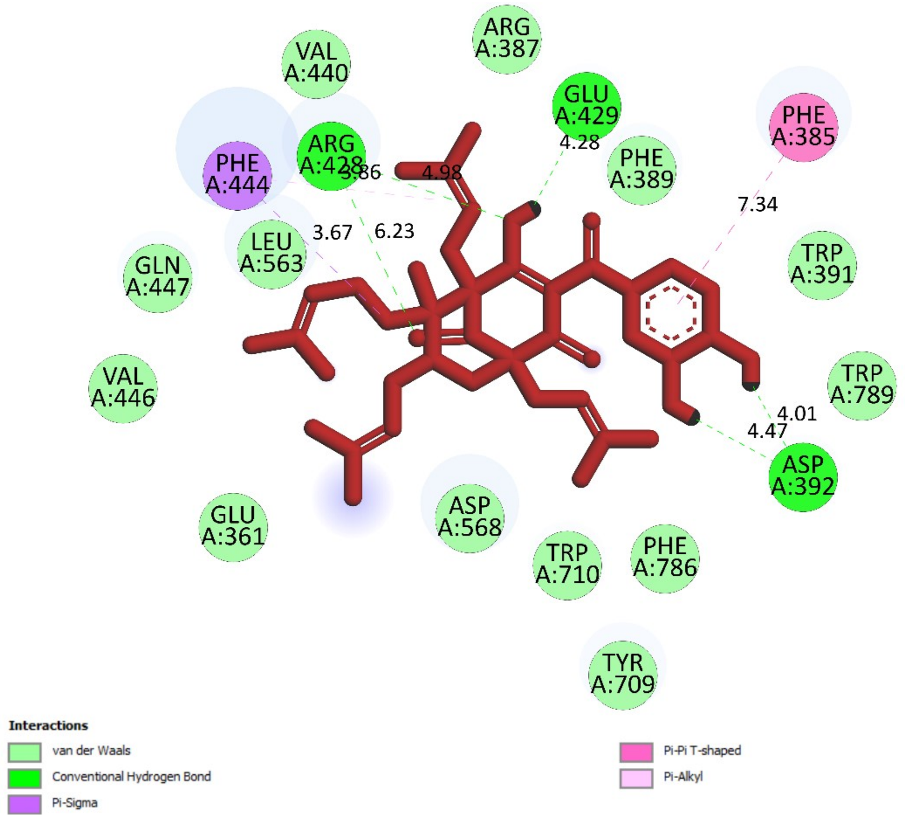

Figure S14. one 2D diagram indicated the significant ligand interactions between pose 82/ compound 3 and residual amino acid on 4J5T enzyme.

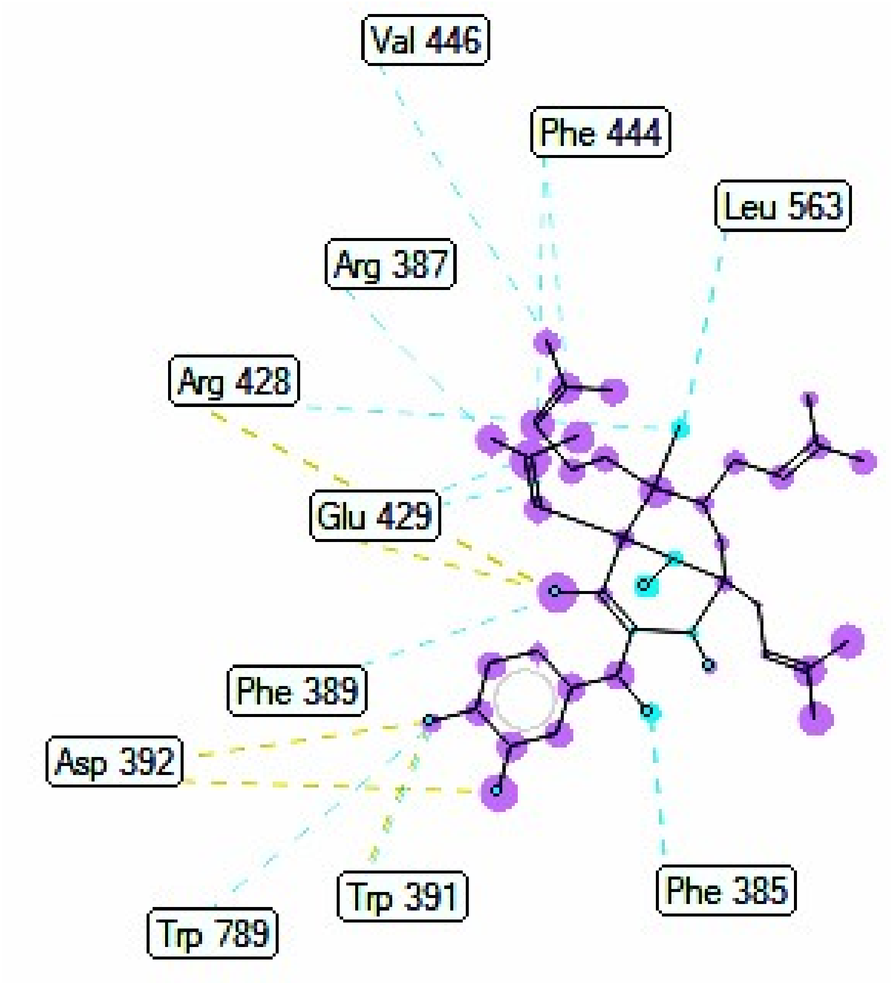

Figure S15. One ligand map indicated the secondary interactions between pose 82/compound 3 and 4J5T enzyme.

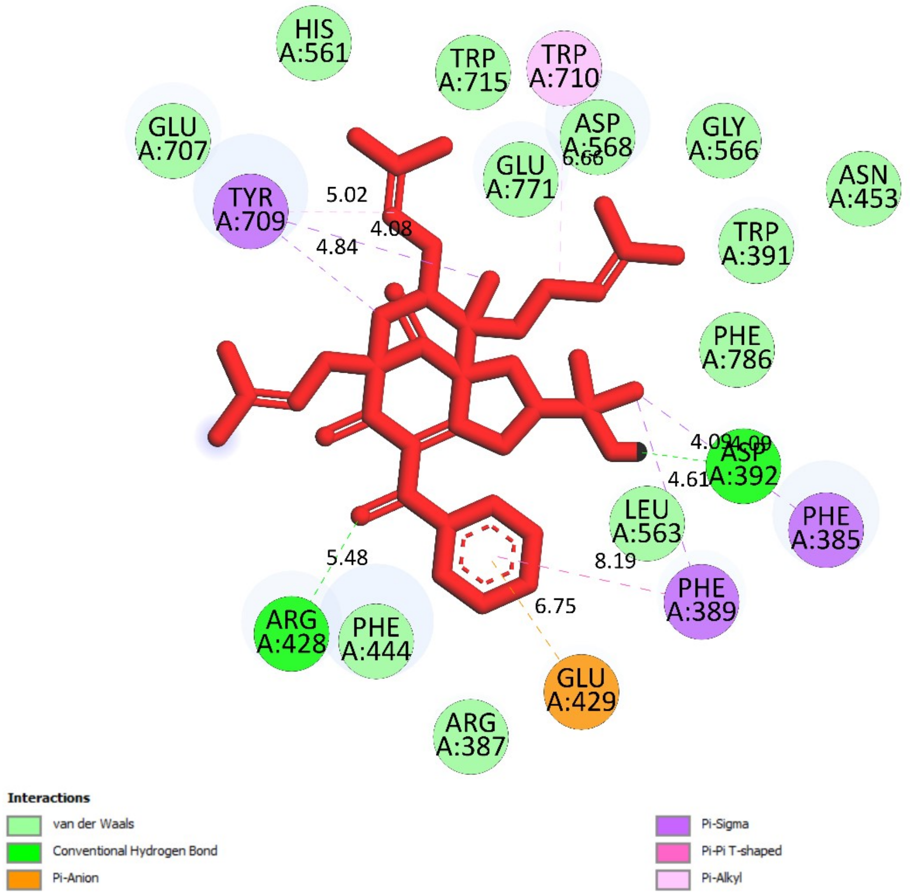

Figure S16. One diagram showed the significant ligand interactions between pose 332/compound 4 and 4J5T enzyme.

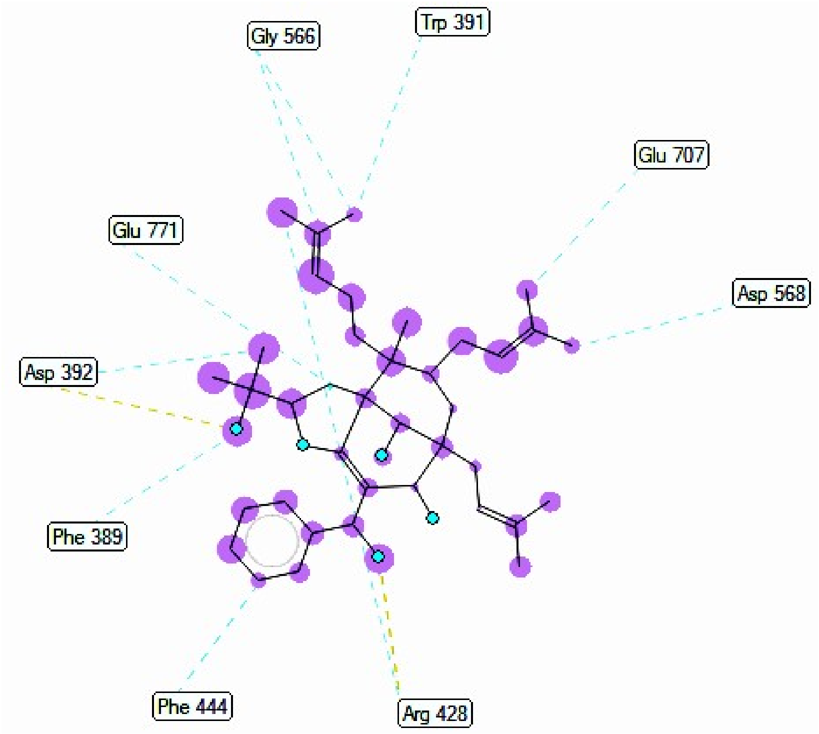

Figure S17. One ligand map showed the secondary interactions between pose 332/compound 4 and 4J5T enzyme.

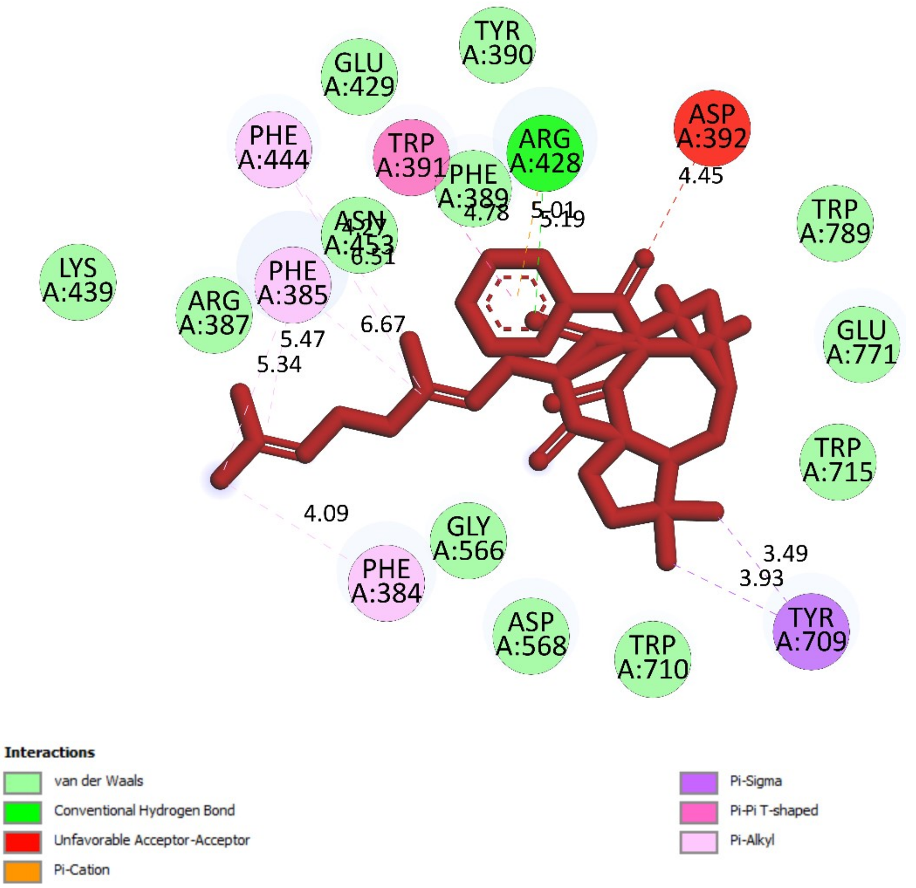

Figure S18. One diagram showed the significant ligand interactions between pose 160/compound 5 and 4J5T enzyme.

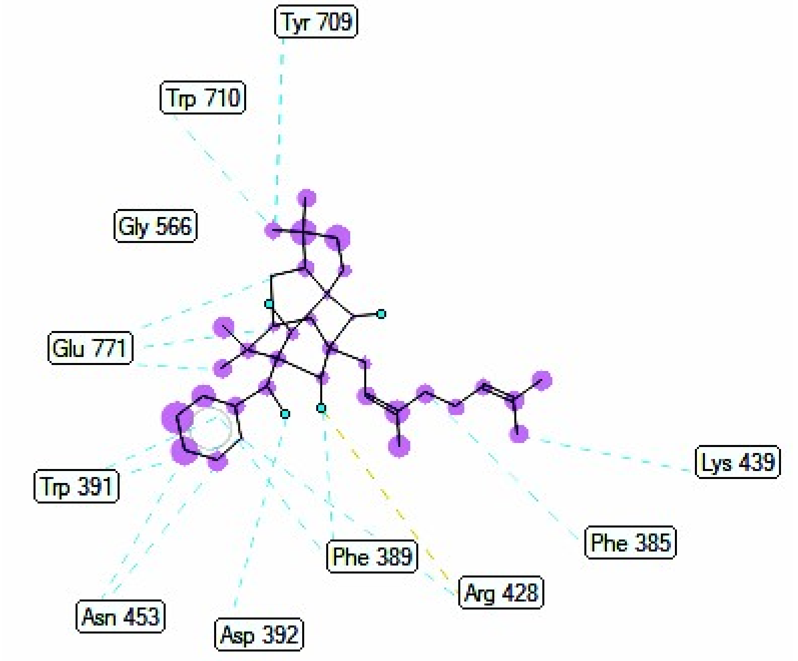

Figure S19. One ligand map showed the secondary interactions between pose 160/compound 5 and 4J5T enzyme.

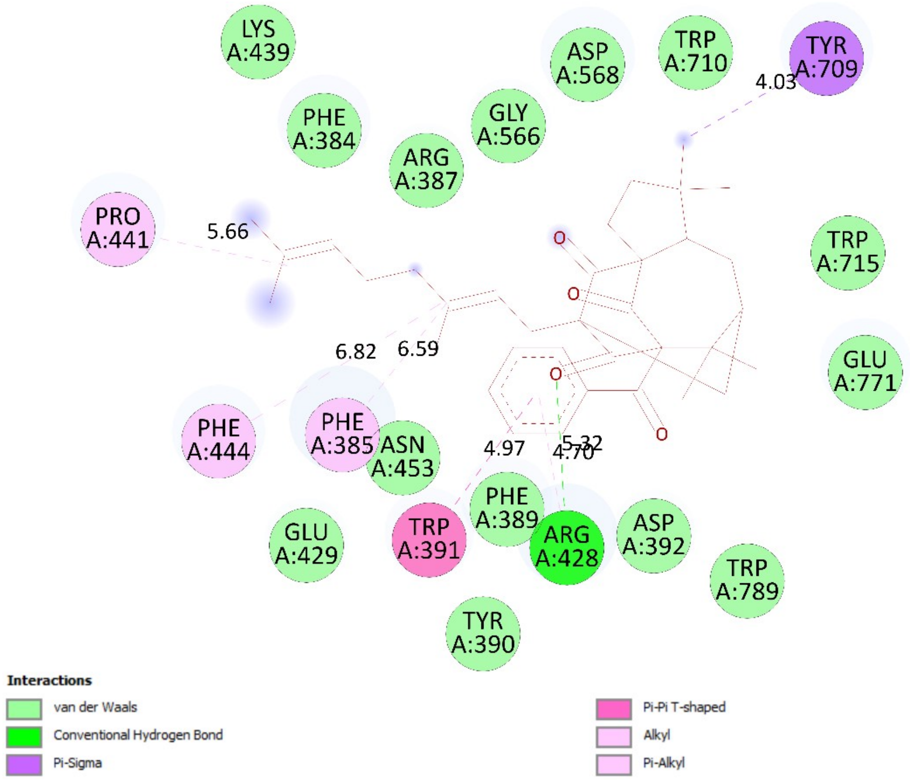

Figure S20. One diagram showed the significant ligand interactions between pose 489/compound 7 and 4J5T enzyme.

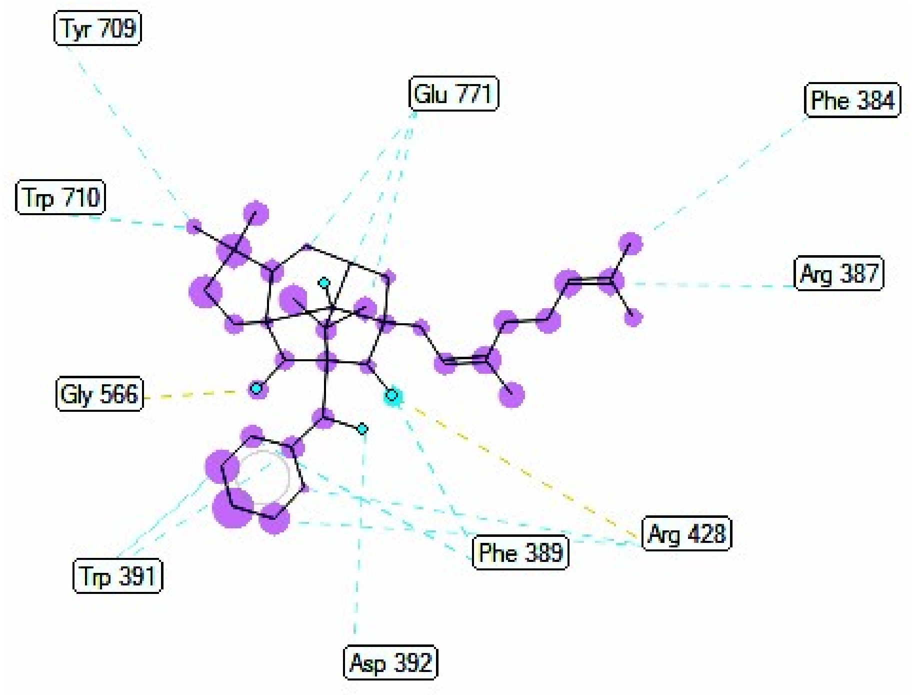

Figure S21. One ligand map showed the secondary interactions between pose 489/compound 7 and 4J5T enzyme.
